# Inorganic phosphate rapidly switches the stability of Arp2/3-induced actin branches

**DOI:** 10.1083/jcb.202605018

**Published:** 2026-07-09

**Authors:** Jiu Xiao, Foad Ghasemi, Adrien Schahl, Miroslav Mladenov, Rebecca Pagès, Matthieu Chavent, Michael Way, Luyan Cao, Guillaume Romet-Lemonne, Antoine Jégou

**Affiliations:** 1 Université Paris-Cité, CNRS, Institut Jacques Monod, Paris, France; 2 Cellular Signalling and Cytoskeletal Function Laboratory, The Francis Crick Institute, London, UK; 3 Department of Infectious Disease, Imperial College, London, UK; 4 Laboratoire de Microbiologie et Génétique Moléculaires (LMGM), Centre de Biologie Intégrative (CBI), Université Toulouse, CNRS, Toulouse, France; 5 SciLifeLab, Department of Applied Physics, KTH Royal Institute of Technology, Solna, Sweden

## Abstract

Actin filaments often appear as branches, nucleated by the Arp2/3 complex. Arp2 and Arp3 are ATPases, which adopt different nucleotide-dependent conformations. We investigated how the nucleotide state of mammalian Arp2/3 complexes affects branch stability, by applying mechanical load. Branch junctions are 30-fold more stable when Arp2/3 is in the ADP–inorganic phosphate (Pi) rather than the ADP state. Pi is in rapid equilibrium with the ADP-Arp2/3 complex at the branch junction (release rate 0.2 s^−1^). Upon branch dissociation, Arp2/3 complexes remaining attached to the mother filament in the ADP-Pi state are 100-fold more stable, release their phosphate slowly (0.05 s^−1^), and can regrow branches without reloading ATP. Glia maturation factor (GMF) accelerates the dissociation of surviving ADP-Arp2/3 complexes, but does not prevent branch regrowth at physiological ATP concentration. Cortactin stabilizes branches and enhances renucleation. Neither GMF nor cortactin affects branch stability and renucleation of ADP-Pi-Arp2/3. Overall, these results identify Pi in the Arp2/3 complex as a critical regulator of branched actin network stability.

## Teaser

Branched actin networks are exposed to mechanical stress. Xiao et al. show that branch junctions whose Arp2/3 complex contains inorganic phosphate are mechanically more stable and are protected from GMF-induced destabilization, but are not stabilized further by cortactin.

## Graphical Abstract

**Figure:**
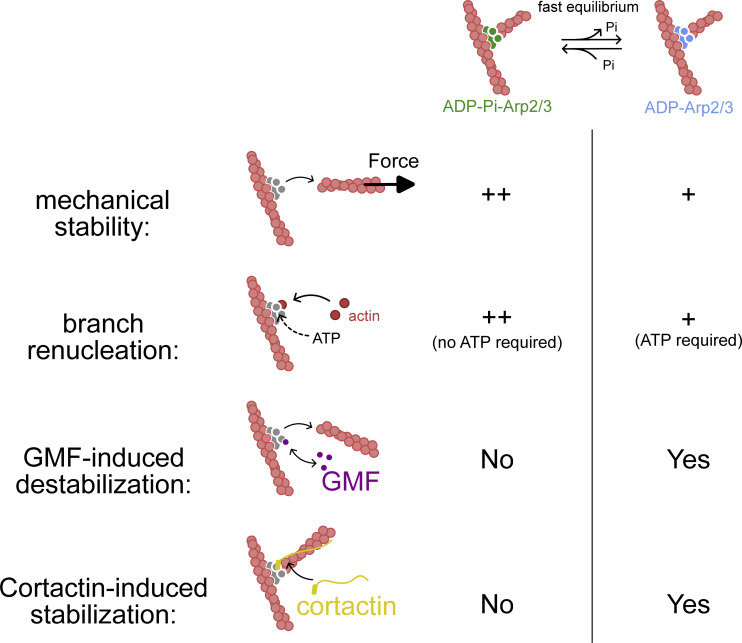

## Introduction

Cells assemble a diverse array of actin filament networks to execute spatially and timely controlled tasks throughout the life cycle, including cell motility, cytokinesis, or the transport of vesicles within the cell. The assembly and disassembly of actin filaments are tightly regulated by tens of regulatory proteins. The Arp2/3 complex, composed of seven subunits, including two actin-related proteins, Arp2 and Arp3, stands out as an essential nucleator of actin filaments, uniquely capable of generating branched structures (reviewed in Gautreau et al. [2022]; Stradal et al. [2025]).

The nucleation of actin filament branches by the Arp2/3 complex is a multistage process. First, two WASP-family nucleation-promoting factors (NPFs) interact with the Arp2/3 complex through their central and acidic domains, bind two actin monomers via their WH2 domains, and bring them into contact with Arp2 and Arp3 (Gautreau et al., 2022; Saks et al., 2025; Shaaban et al., 2020; van Eeuwen et al., 2023). This first step induces allosteric changes leading to the positioning of Arp2 and Arp3 in a side-by-side conformation that resembles the short-pitch actin filament conformation. For its final full activation, the NPF–Arp2/3–actin complex must then bind to the side of a preexisting actin filament (hereafter referred to as the “mother filament”) and the two NPFs must detach from the Arp2/3–actin complex. This final step allows the growth of a filament (hereafter referred to as the “daughter filament” or “branch”) by the addition of actin subunits to the newly created barbed end of the Arp2/3–actin complex. This activation of the Arp2/3 complex is accompanied by a flattening of Arp2 and Arp3 subunits (Gautreau et al., 2022; Shaaban et al., 2020), analogous to the transition of actin from its globular (G-actin) to filamentous (F-actin) conformations upon incorporation at the barbed end of a filament (Oda et al., 2009).

To nucleate a branch and be activated by NPFs, Arp2 and Arp3 need to have ATP bound to their nucleotide pocket (Dayel et al., 2001). For bovine or Acanthamoeba Arp2/3 complexes, bulk biochemical studies using radiolabeled ATP showed that ATP hydrolysis within Arp2 alone accompanies branch formation (Dayel et al., 2001; Dayel and Mullins, 2004; Le Clainche et al., 2003). In contrast, yeast or *Drosophila* Arp2/3 complexes with either an Arp2 or an Arp3 mutant defective for ATP hydrolysis are still competent for branch formation, but not when both mutations are present (Ingerman et al., 2013; Martin et al., 2006). This points to an intricate relationship between the nucleotide state of the Arp2/3 complex and its activation. In addition, these ATP-defective Arp2 and Arp3 point mutations also decrease the stability of nucleated branches, further highlighting the importance of the nucleotide state of the Arp2/3 complex. We have recently shown that when a branch detaches, the mammalian Arp2/3 complex remains mostly bound to the mother filament, where it quickly releases its ADP and reloads ATP in order to nucleate a new branch without interacting with an NPF (Ghasemi et al., 2024).

The structure of the Arp2/3 complex at a branch junction has been extensively characterized using cryo-electron microscopy (cryo-EM) (Chou et al., 2022; Ding et al., 2022; Fäßler et al., 2020; Liu et al., 2024). For the bovine Arp2/3 complex, cryo-EM maps of mature branches indicate that both Arp2 and Arp3 have ADP in their nucleotide-binding pocket (Ding et al., 2022). The Arp2/3 complex thus transitions from the ADP–inorganic phosphate (Pi) state toward the ADP state by releasing Pi. ADP-Arp2/3 complex branch junctions have been reported to be less stable than their ADP-Pi counterparts, although this difference was only apparent in the presence of a pulling force (Dayel and Mullins, 2004; Le Clainche et al., 2003; Pandit et al., 2020). Moreover, how fast Pi is released from the Arp2/3 complex has remained challenging, with debated rates, depending on the Arp2/3 complex species and the experimental methods employed: it varies from seconds for Acanthamoeba Arp2/3 complexes (Dayel et al., 2001), to minutes for bovine (Le Clainche et al., 2003) or *Schizosaccharomyces pombe* yeast (Pandit et al., 2020) Arp2/3 complexes. Within actin subunits, the nucleotide that is present tunes the kinetic rates of assembly/disassembly at filament ends, as well as the interaction of the filament with essential regulatory proteins (Lappalainen et al., 2022).

Glia maturation factor (GMF) is a member of the ADF-homology protein family (reviewed in Goode et al. [2018]). Mammals express two isoforms in a tissue-dependent manner, GMFβ and GMFγ, sharing 82% sequence identity and strong structural similarities. GMF plays an important role in regulating lamellipodium dynamics and controlling cell motility (Poukkula et al., 2014). Although structurally very similar to other members of the family such as cofilin, GMF does not bind to actin but only interacts with the Arp2/3 complex. When interacting with the Arp2/3 complex in the inactive splayed conformation, in solution, GMF binds to Arp2 and ArpC1 (Luan and Nolen, 2013). Moreover, GMF preferentially binds soluble Arp2/3 complexes in the ADP state (Boczkowska et al., 2013), reminiscent of the nucleotide-dependent affinity of cofilin for actin (Blanchoin and Pollard, 1999). Cortactin is a multidomain protein that regulates cell migration (see Schnoor et al. [2018] for a review). Cortactin is known to stimulate Arp2/3 complex activation in synergy with NPFs (Helgeson et al., 2014; Helgeson and Nolen, 2013), by stabilizing the short-pitch active conformation of the Arp2/3 complex (Fregoso et al., 2023). Notably, cortactin directly targets branch junctions by binding simultaneously to the Arp3 subunit via its NtA domain and to the first actin subunits of the daughter filament via its 6.5 actin-binding repeats (Liu et al., 2024). This binding mode has been proposed to stabilize branch junctions by preventing the dissociation of the daughter filament from the Arp2/3 complex (Liu et al., 2024; McGuirk et al., 2024). The cryo-EM structure from Liu and colleagues shows cortactin bound to an ADP-Arp2/3 complex branch junction (Liu et al., 2024), but how cortactin-bound branches respond to mechanical forces, and whether cortactin is sensitive to the nucleotide state of the Arp2/3 complex at the branch junction remain open questions.

Given this, one may expect that the nucleotide state of the Arp2/3 complex at the branch junction should influence the mechanical stability of branches and how proteins such as GMF, cortactin, or coronin (reviewed in Cao and Way [2024]; Stradal et al. [2025]) bind to Arp2 or Arp3 subunits at the branch junction. These effects would have important consequences for the overall architecture and dynamics of branched actin networks in cells. To investigate this, we need to take into account the impact of mechanics on branch stability, the ability of Arp2/3 complexes to renucleate a branch, and that branch renucleation from an Arp2/3 complex in the ADP state requires reloading a fresh ATP (Ghasemi et al., 2024).

In this study, we investigate the mechanical stability of mammalian Arp2/3 complex branch junctions as a function of their nucleotide state, both in the presence and in the absence of regulatory proteins, human GMFγ or mouse cortactin. We find that Pi is in rapid equilibrium with the Arp2/3 complex at the branch junction, and significantly enhances branch stability. Following branch dissociation, the departure of the surviving Arp2/3 complex in the ADP-Pi state from the mother filament is more than two orders of magnitude slower than that in the ADP state, leading to an improved branch renucleation rate. Furthermore, the ADP-Pi state of the Arp2/3 complex prevents both GMF-induced debranching and cortactin-induced protection. We propose that Pi acts as a rapid molecular switch that controls the turnover of branched actin networks within cells.

## Results

### Branch dissociation rate depends on the Arp2/3 nucleotide state

**Figure 1.**
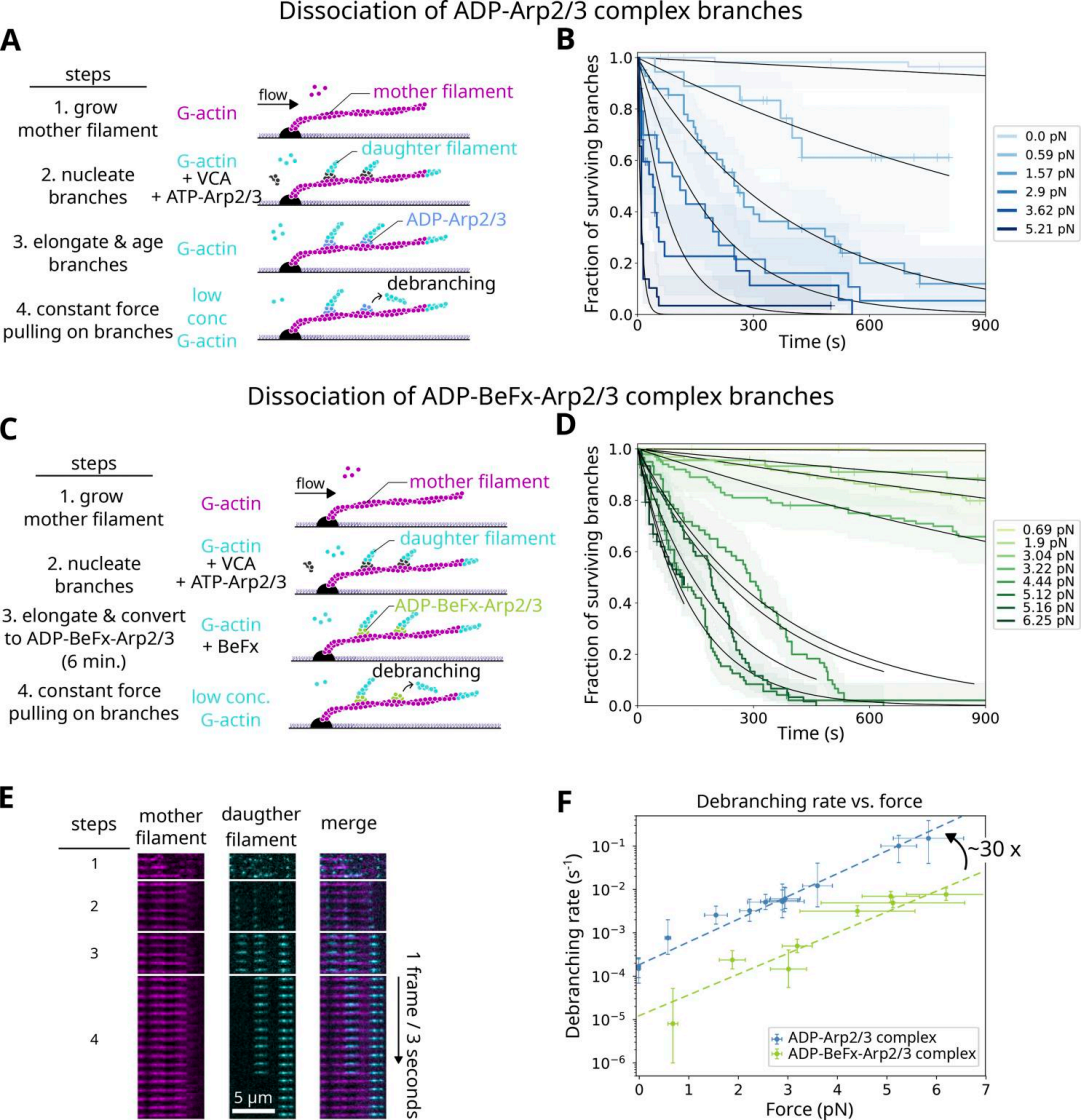
**Stability of an Arp2/3 complex branch junction depends on its bound nucleotide. (A)** Schematic of the debranching experiment using microfluidics to pull on ADP-Arp2/3 complex branches. Mother filaments are elongated from surface-anchored spectrin–actin seeds by flowing in 0.7 µM Alexa Fluor 488 (10%)–G-ATP-actin (magenta) for 5 min. Branches are nucleated by flowing in 0.4 µM Alexa Fluor 568 (10%)–G-ATP-actin (cyan), with VCA domains from N-WASP, and sheep Arp2/3 complex. They are then elongated and aged for 30 min by flowing in 0.2 µM Alexa Fluor 568 (10%)–G-ATP-actin alone. The dissociation of branches under constant pulling force is then monitored over time by flowing in 0.15 µM Alexa Fluor 568 (10%)–G-ATP-actin at different flow rates. The flowing solution applies a pulling force to the mother filaments and the branches. **(B)** Surviving fractions of ADP-Arp2/3 complex branches over time, using different flow rates to apply different constant forces using the procedure shown in A. Black curves are single-exponential fits (number of analyzed branches = 60 [0 pN], 18 [0.59 pN], 42 [1.57 pN], 25 [2.9 pN], 25 [3.62 pN], and 29 [5.21 pN]). Shaded areas are 95% confidence intervals. Vertical tick marks on the surviving curves indicate censoring events (see Materials and methods). **(C)** Schematic of the debranching experiment for ADP-BeFx-Arp2/3 complex branches. Steps 1 & 2 are similar to the ones in A. At step 3, branches are elongated for 6 min by flowing in 0.4 µM Alexa Fluor 568 (10%)–G-ATP-actin alone in a buffer containing 6 mM BeFx. At step 4, the dissociation of branches is observed over time in a regular buffer containing only 0.15 µM Alexa Fluor 568 (10%)–G-ATP-actin at different flow rates. **(D)** Surviving fractions of ADP-BeFx-Arp2/3 complex branches over time, using different flow rates to apply different constant forces using the procedure shown in C. Black curves are single-exponential fits (number of analyzed branches = 132 [0.69 pN], 100 [1.9 pN], 45 [3.04 pN], 100 [.22 pN], 60 [4.44 pN], 100 [5.12 pN], 60 [5.16 pN], and 106 [6.25 pN]). Shaded areas are 95% confidence intervals. Vertical tick marks on the surviving curves indicate censoring events. **(E)** Time-lapse images showing the different steps described in A, for branch formation and dissociation, with the actin mother filament in magenta, and actin branches in cyan. The scale bar is 5 µm. **(F)** Debranching rates as a function of constant pulling forces (log-linear representation), for ADP (blue)- and ADP-BeFx (green)-Arp2/3 complex branch junctions. Error bars are standard deviations for the force, and confidence intervals on the single-exponential fits, obtained by fitting the upper and lower bounds of the 95% confidence intervals of the survival fractions from debranching experiments. For ADP-Arp2/3 complex branch junctions, data points at 0 pN are from experiments performed in open chambers, without any flow (2 replicates, Fig. S1, C and D). Data are obtained from experiments with at least 20 analyzed branches for each condition. Dashed lines are single-exponential fits of the experimental data points.

To investigate the mechanical stability of Arp2/3-mediated actin filament branches, we employed an *in vitro* microfluidics-based assay (Ghasemi et al., 2024; Jégou et al., 2011). Actin filaments were elongated from spectrin–actin seeds anchored to the glass surface, and branches were nucleated and grown by exposing these filaments to a solution containing the VCA domain of N-WASP, Arp2/3 complex, and actin (Fig. 1, A and B). Branches were then exposed to a flowing solution of actin, to induce elongation and apply a controlled force (viscous drag) to them (Jégou et al., 2013). Experiments were carried out using mammalian Arp2/3 complexes, with alpha-skeletal actin from rabbit skeletal muscle, in a buffer at pH 7, at 25°C (see Materials and methods).

**Figure S1.**
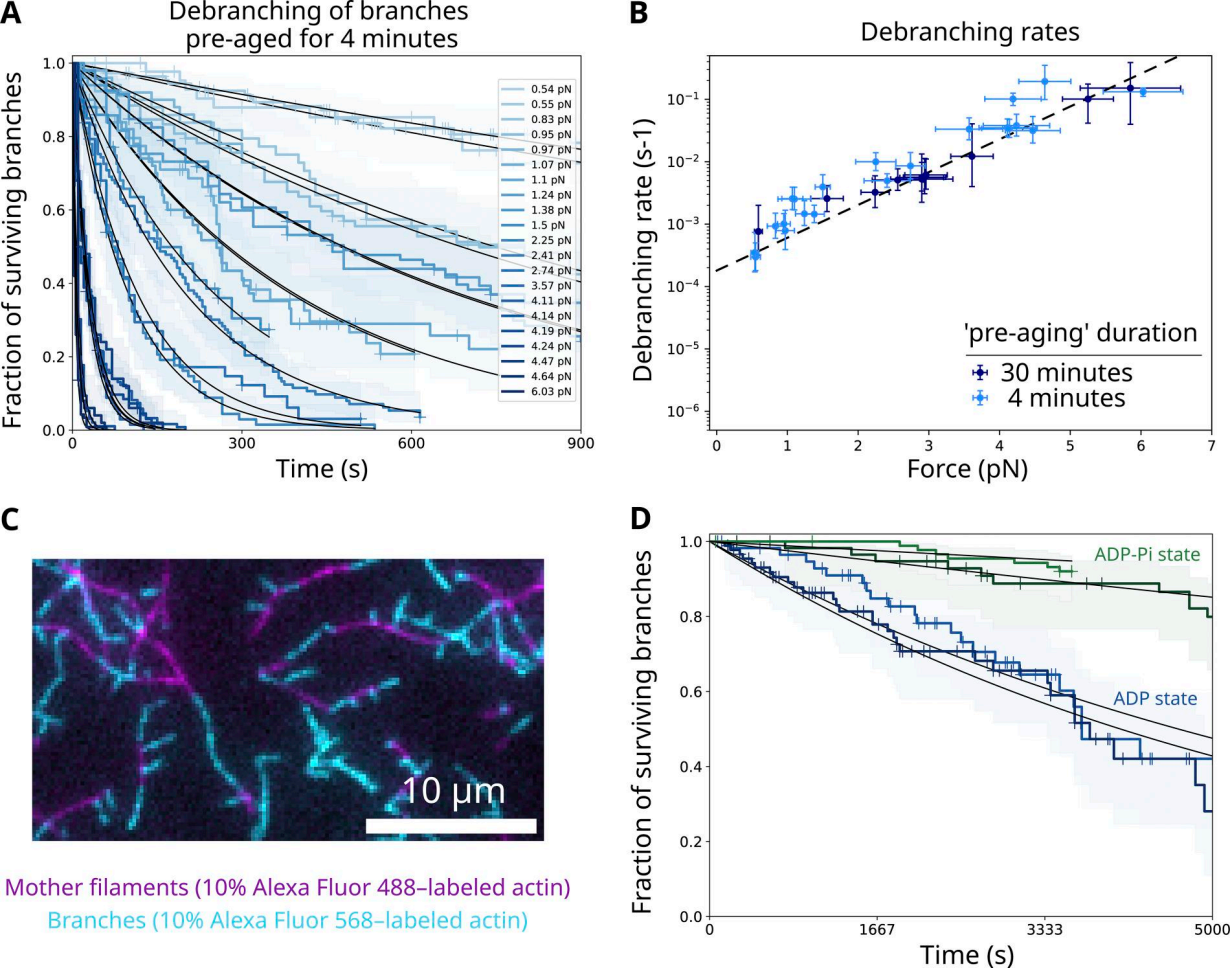
**Impact of Arp2/3 complex aging and force on the debranching rate. (A)** Surviving fractions of branches monitored over time using different flow rates to apply different constant forces, while flowing in 0.15 µM Alexa Fluor 568 (10%)–G-ATP-actin. Black curves are single-exponential fits. The average force for each experiment is indicated. Each curve is from a single experiment with at least 50 analyzed branches. Shaded areas are 95% confidence intervals. Vertical ticks on the surviving curves are times where censoring events occurred. **(B)** Debranching rates as a function of constant pulling forces, for ADP-Arp2/3 complex branch junctions, pre-aged for 4 (light blue) or 30 (blue) minutes. Error bars are standard deviations for the force, and confidence intervals on the single-exponential fits, obtained by fitting the upper and lower bounds of 95% confidence intervals of the survival fractions of debranching experiments. Data are obtained from experiments with at least 50 and 18 analyzed branches for 4- and 30-min pre-aging duration, respectively. The dashed line is a single-exponential fit of the 30-min pre-aging data. **(C)** Branches (cyan) on mother filaments (magenta) are exposed to a solution of 0.04 µM 10% Alexa Fluor 568–labeled G-actin in F-buffer, 50 mM KCl, and 0.5% methylcellulose, in the presence or absence of 50 mM phosphate. Images were acquired at a frame rate of 1 frame every 30 s in TIRF for at least 60 min. The scale bar is 10 µm. **(D)** Surviving fractions of branches monitored over time. Shaded areas are 95% confidence intervals. Vertical ticks on the surviving curves are times where censoring events occurred. Black curves are single-exponential fits with characteristic rates of 1.49 (±0.9) × 10^−4^ (*n* = 60 branches) and 1.7 (±0.7) × 10^−4^ (*n* = 90) s^−1^ for ADP-Arp2/3 complex branches; 3.2 (±1.6) × 10^−5^ (*n* = 60) and 1.48 (±1) × 10^−5^ (*n* = 90) s^−1^ for Arp2/3 complex branches in the presence of 50 mM phosphate.

First, we examined the destabilization of ADP-Arp2/3 complex branch junctions by applying constant pulling forces to them. Given the variability in the reported ATP hydrolysis and Pi release rates for Arp2/3 complexes, we chose to age branches for 30 min to ensure Arp2/3 complexes were in the ADP state before monitoring branch dissociation (Fig. 1, A and E). For each constant force experiment, the fraction of intact branches versus time (survival curve) was fitted with a single-exponential function to derive the debranching rate at that specific force (Fig. 1 B). For all forces, the survival curves exhibit a clear single-exponential behavior, indicating a constant detachment rate over time. The debranching rate seems to increase exponentially with the applied pulling force, in the range of 0–6 pN (Fig. 1 F; see more refined analysis below). This behavior of accelerated debranching with the increase of the applied force is similar to the “slip bond” concept, as predicted by the Bell–Evans model (Bell, 1978; Dudko et al., 2008) of the force-dependent lifetime of the interaction between two proteins. Similar debranching rates were obtained when branches were aged for only 4 min (Fig. S1, A and B), indicating that either all Arp2/3 complexes are already in the ADP state, or that ADP- and ADP-Pi-Arp2/3 complex branch junctions detach with the same rate.

Beryllium fluoride (BeFx) is a structural analog of Pi that has been classically used to probe actin filament assembly by mimicking the ADP–Pi– or ATP-actin state (Combeau and Carlier, 1988; Muhlrad et al., 1994; Orbán et al., 2008), and to investigate the different structural conformations of actin filament subunits based on their nucleotide state (Merino et al., 2018; Oosterheert et al., 2022). Notably, BeFx remains stably bound within the F-actin nucleotide pocket for hours (Combeau and Carlier, 1988). It was previously shown that BeFx stabilizes *S. pombe* Arp2/3 complex branch junctions (Pandit et al., 2020). Recent cryo-EM observations by Oosterheert and colleagues did not report any BeFx bound on the surface of actin filaments (Oosterheert et al., 2022). We thus assume that BeFx would bind only within the nucleotide pocket of Arp2 and/or Arp3, not on the surface of the Arp2/3 complex. We next sought to investigate the force response of Arp2/3 complex branch junctions in the ADP-BeFx state, as a tool to mimic a stable ADP-Pi-like Arp2/3 complex at the branch junction.

**Figure S2.**
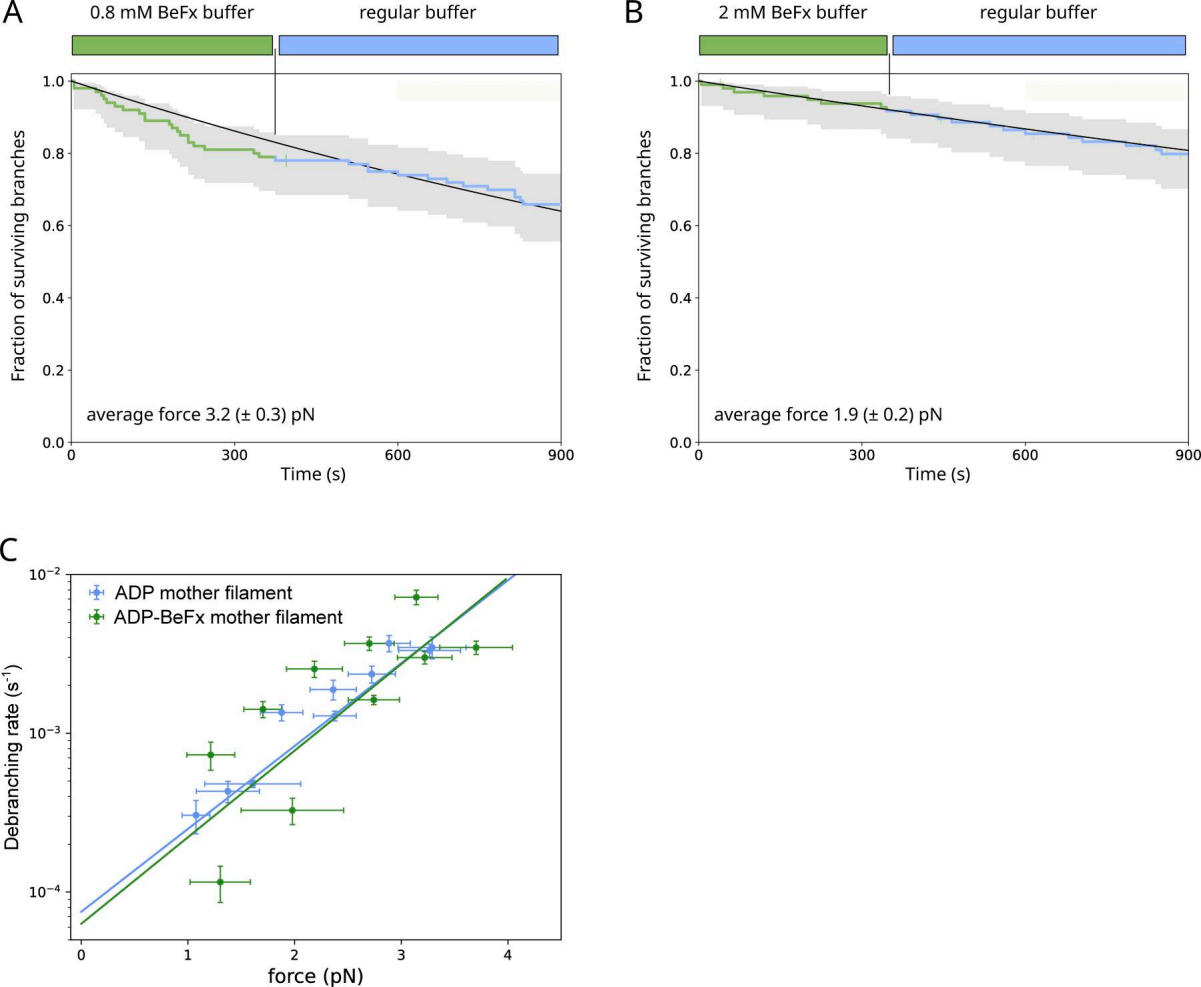
**Nucleotide state of the mother filament does not affect debranching rate. (A and B)** Branches initiated as detailed in Fig. 1 A are exposed to a solution containing either 0.8 (A) or 2 (B) mM BeFx for 4 min. Debranching events are recorded first in the same 0.8 or 2 mM BeFx buffer, before switching to a regular buffer solution. All buffer conditions contain 0.1 µM Alexa Fluor 568 (10%)–G-ATP-actin. For each condition, 100 branches were analyzed. Image acquisition rate is 1 frame every 5 s. **(C)** ADP-Arp2/3 complex branches initiated either from ADP- or ADP-BeFx-actin mother filaments debranch at the same rate under 1- to 4-pN pulling forces. For both conditions, branches are exposed to 0.15 µM actin upon the start of the debranching phase. Each individual point is from a single experiment with at least 62 analyzed branches. Debranching rates are obtained from single-exponential fit from survival fraction curves. Error bars are 95% confidence intervals from those fit. Lines represent single-exponential fits from each population of debranching rates.

Branches were formed with ATP-Arp2/3 complexes and then aged in the presence of 2 mM BeFx for 6 min prior to force-induced debranching in a buffer lacking BeFx (Fig. 1, C and D). The debranching rate for ADP-BeFx-Arp2/3 complex branch junctions followed the same exponential behavior with increasing force but was consistently around 30 times lower than for ADP-Arp2/3 complex branches (Fig. 1 F). We also observed that debranching was not affected by the presence or absence of BeFx in solution (Fig. S2, A and B) and that the nucleotide state of the mother filament did not impact debranching (Fig. S2 C). These observations allowed us to conclude that branch junctions with the Arp2/3 complex in the ADP-BeFx state detach more slowly than in the ADP state.

### Pi readily associates and dissociates from Arp2/3 complexes at branch junctions

**Figure 2.**
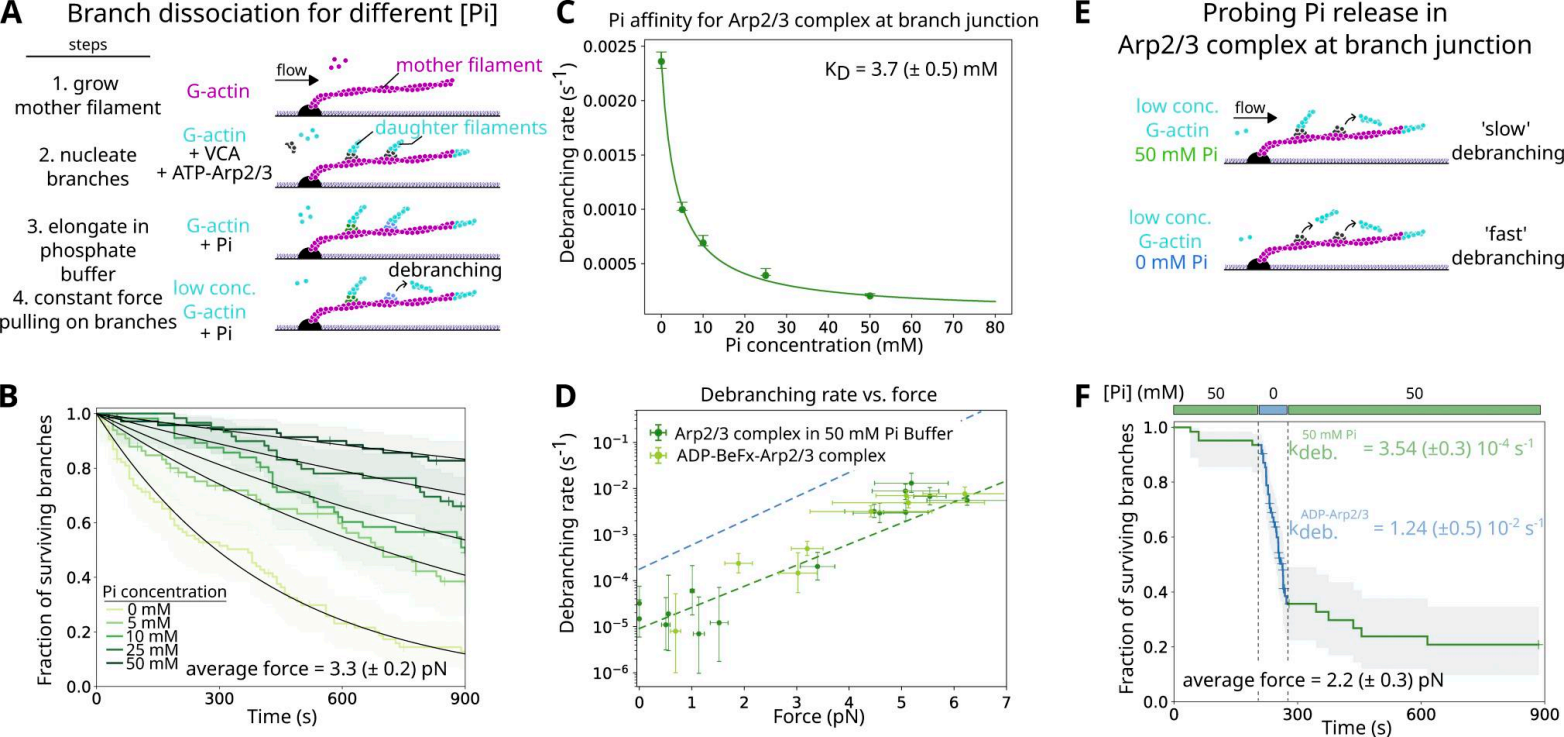
**Pi is in rapid equilibrium with the Arp2/3 complex at branch junctions. (A)** Schematic of the debranching experiment for Arp2/3 complex branch junctions exposed to buffers containing different Pi concentrations. From step 3, branches, initiated as in Fig. 1 A, are constantly exposed to a phosphate buffer. **(B)** Surviving fractions of branches monitored over time by applying an average force of 3.3 (±0.2) pN, and exposing them to buffers of different Pi concentrations, containing 0.15 µM Alexa Fluor 568 (10%)–G-ATP-actin. Black curves are single-exponential fits (number of analyzed branches = 86 [0 mM Pi], 66 [5 mM Pi], 56 [10 mM Pi], 70 [25 mM Pi], and 60 [50 mM Pi]). Shaded areas are 95% confidence intervals. Vertical ticks on the surviving curves are times where censoring events occurred. **(C)** Debranching rates as a function of Pi concentration, obtained from the fits of the curves shown in B. Error bars are confidence intervals on the single-exponential fits, obtained by fitting the upper and lower bounds of 95% confidence interval curves. The line is the result of a hyperbolic fit yielding K_D_, the equilibrium dissociation constant of Pi from the Arp2/3 complex at branch junction. **(D)** Debranching rates as a function of constant pulling forces, for Arp2/3 complex branch junctions exposed to a 50 mM Pi buffer solution (green), and for ADP-BeFx-Arp2/3 complex branch junctions (light green). Error bars are standard deviations for the force, and confidence intervals on the single-exponential fits, obtained by fitting the upper and lower bounds of 95% confidence intervals of the survival fractions of debranching experiments. For each force condition, data are from experiments with on average 100 analyzed branches for branches exposed to 50 mM Pi and 90 analyzed branches for ADP-BeFx-Arp2/3 complexes. The green dashed line is a single-exponential fit of the 50 mM Pi data. The blue dashed line is from Fig. 1 F for ADP-Arp2/3 complex branches. **(E)** Schematic of the debranching experiment of Arp2/3 complex branch junctions exposed to 50 mM Pi or regular buffer, with a fast flow switching between the two conditions (<1 s). **(F)** Surviving fraction of branches monitored over time by applying an average force of 2.3 (±0.3) pN and exposing them to either regular or 50 mM phosphate buffer, both containing 0.1 µM Alexa Fluor 568 (10%)–G-actin. Debranching rates obtained from single-exponential fits (not shown) of the different regimes of the curves are indicated (see also Fig. S3 D). 5 s before the buffer condition is rapidly changed by microfluidics, the acquisition frame rate is increased from 1 to 2 frames per second. Censoring events are indicated by vertical ticks. *n* = 62 analyzed branches.

**Figure S3.**
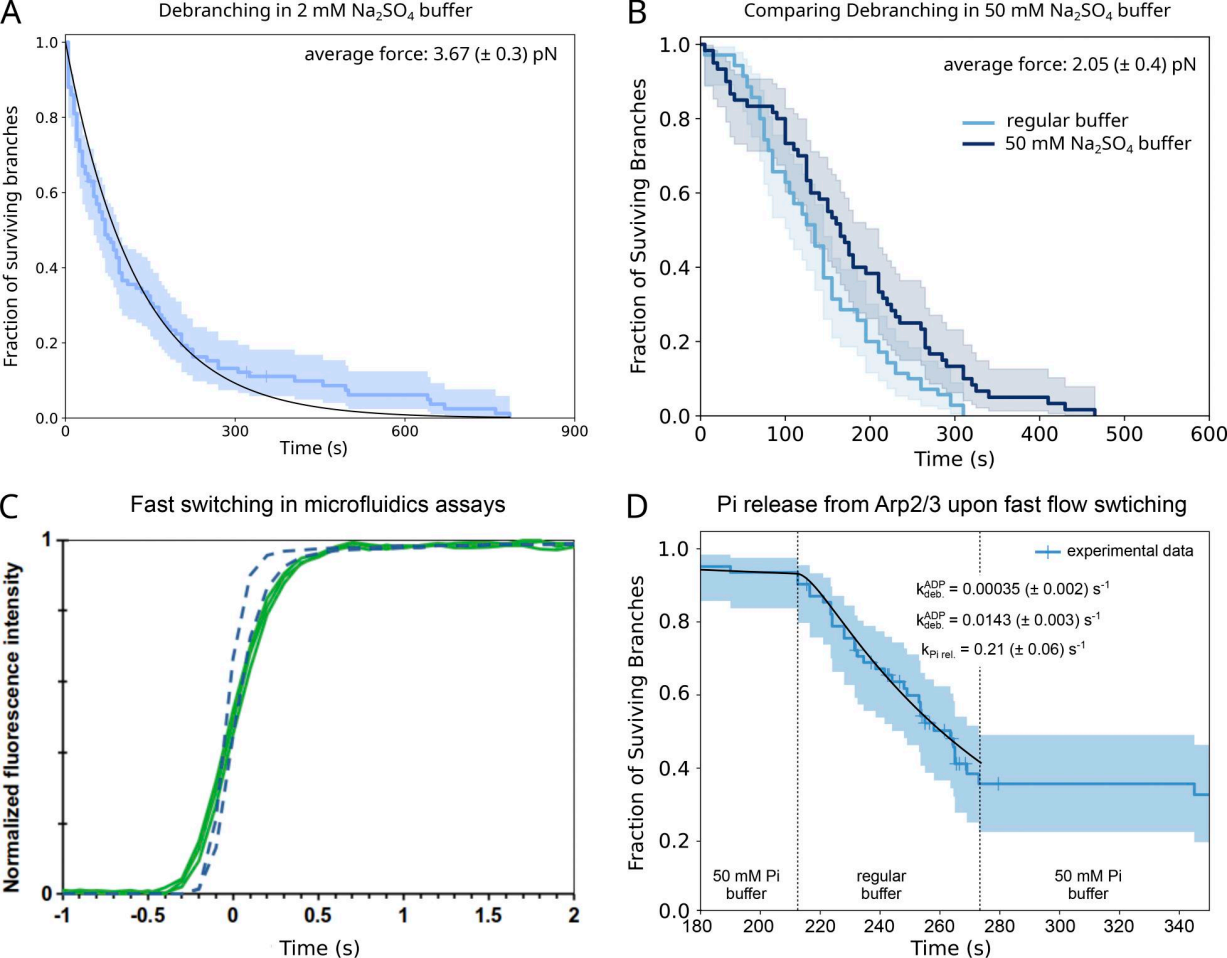
**Pi release rate from within the Arp2/3 complex at branch junctions. (A)** ADP-Arp2/3 complex branches dissociate equally fast in Na_2_SO_4_ or regular buffer. Fraction of surviving ADP-Arp2/3 complex branch junctions exposed to a buffer with 0.15 µM actin and 0.8 mM Na_2_SO_4_, 23.4 mM NaF, and 50 mM KCl, with an average pulling force of 3.67 (±0.3) pN. The exponential fit (black line) yields a debranching rate of 7.9 10^−3^ (±0.3) s^−1^, in agreement with the expected rate for ADP-Arp2/3 complex branches (Fig 1 F). The acquisition time interval is 5 s. Number of analyzed branches = 100. **(B)** Fraction of surviving ADP-Arp2/3 complex branch junctions exposed to a buffer with 0.3 µM actin and either 50 mM KCl or 50 mM Na_2_SO_4_. The average force at the time of debranching is 2.05 pN. (*n* = 70 (50 mM KCl) and 60 (50 mM Na_2_SO_4_) branches analyzed). **(C)** Fast switching between two buffer conditions in microfluidics. Increase in fluorescence intensity upon flow switching in a microfluidics assay, with a flow rate of 10,000 nL/min. Depending on the size of the fluorescent compounds, either Alexa dye (blue, dashed line) or Alexa-labeled actin (green), the switching is typically between 0.5 and 1 s. Fluorescence images were recorded at 10 images per second in TIRF mode, with an evanescent penetration depth of 150 nm. All curves are positioned with time 0 when the normalized intensity reaches 0.5. **(D)** Estimating the Pi release rate of the Arp2/3 complex at branch junctions. Upon switching from 50 mM Pi buffer to regular buffer, the branch survival fraction decreases faster as a function of time. According to the reaction scheme presented in Fig. 8 A, Pi is released from the Arp2/3 complex at the branch junction, with a rate k_Pi rel_. Debranching occurs at a rate k_deb., ADP-Pi_ or k_deb., ADP_, for ADP-Pi- or ADP-Arp2/3 complex branches, respectively. The observed branch survival fraction thus evolves as (see Materials and methods) [(k_deb.,ADP_ - k_deb.,ADP-Pi_).exp(-(k_deb.,ADP-Pi_+k_Pi rel_).t)-k_Pi rel_.exp(-k_deb., ADP_.t)]/(k_deb., ADP_-k_deb.,ADP-Pi_-k_Pi rel_). The black line is the fit of this function to the experimental data (in blue, from Fig. 2 F), with three rates as free parameters. We obtained k_deb., ADP_ = 0.0143 s^−1^, k_deb., ADP-Pi_ = 0.00035 s^−1^ in reasonable agreement with the rate determined at 2.2 pN in separate experiments, and k_Pi rel_ = 0.21 s^−1^.

To probe the affinity of the Arp2/3 complex at branch junctions to Pi, we investigated Arp2/3 complex branch stability in buffers with different phosphate concentrations, at a given pulling force (∼ 3.3 pN, Fig. 2 A). The debranching rate gradually decreased with increasing phosphate concentration (Fig. 2, B and C). The ionic strength of the phosphate buffer, which impacts protein solubility, could affect the debranching rate. To examine whether this is the case, branches were exposed to a buffer containing 50 mM sodium sulfate in place of Pi. Branches detached at the same rate as ADP-Arp2/3 complex branches in our standard buffer (Fig. S3, A and B). This indicates that ions with similar protein stabilizing capacity play no role in maintaining branch junctions, suggesting that the stabilization of branches in the presence of phosphate results from the binding of Pi within the nucleotide pocket of Arp2 or Arp3. Fitting the data with a hyperbolic saturation function yields the equilibrium constant (K_D_) for Pi binding to the ADP-Arp2/3 complex at a branch junction of 3.7 (±0.5) mM (Fig. 2 C).

In the presence of 50 mM of Pi, the debranching rate increased in an exponential fashion with force (Fig. 2 D), as is observed for ADP- and ADP-BeFx-Arp2/3 complex branch junctions, with values compatible with the ones measured for ADP-BeFx-Arp2/3 complex branch junctions. Given the affinity constant we measured, in the presence of 50 mM Pi, Arp2/3 complexes at branch junctions are 93% of the time in the ADP-Pi state. If we consider that ADP-BeFx is a good mimic of the ADP-Pi state for Arp2/3 complexes at branch junctions, and because the debranching rate of ADP-Arp2/3 complex branch junctions is 30-fold faster than in the ADP-BeFx state, this would mean that in the presence of 50 mM phosphate, 32% of the debranching events occurs with Arp2/3 complexes in the ADP-Pi state, and 68% in the ADP state (Curtin–Hammett principle [Haupert et al., 2009]; see Materials and methods).

Finally, we also performed experiments in standard “open” chambers to assess the impact of the nucleotide in the absence of pulling force (Fig. S1, C and D). The debranching rates of ADP- and ADP-Pi-Arp2/3 complex branch junctions remained significantly different without pulling force (Fig. 2 D).

We next investigated the kinetics of Pi release from Arp2/3 complexes. We used microfluidics to quickly change the solution surrounding the filament branches, in ∼0.5 s (Fig. S3 C) (Wioland et al., 2022), while imaging branch dissociation at a rate of 2 frames per second. Following branch formation, we recorded branch dissociation in a 50 mM phosphate buffer before rapidly switching to a regular buffer with no phosphate, and then switching back to the initial 50 mM phosphate buffer after a few tens of seconds (Fig. 2 E). Throughout the process, we applied a constant 2.2-pN pulling force. For each phase of the experiment, branches detached at rates similar to those determined previously for either ADP-Pi- or ADP-Arp2/3 complex branch junctions (Fig. 2, E and F). Upon buffer switching, the survival curve of the branches displays a sharp change of slope, indicative of an abrupt transition between two regimes. By fitting the second regime with a model where Arp2/3 complex branches can either debranch in the ADP-Pi state, or first release their Pi and then debranch in the ADP state (see Materials and methods), we obtained a Pi release rate from Arp2/3 complexes of 0.21 (±0.06) s^−1^ (Fig. S3 D). This indicates that Pi is in rapid equilibrium with the Arp2/3 complex at branch junctions, since debranching is slower than 0.01 s^−1^ in all the conditions we tested (Fig 1 F and Fig. 2 D).

**Figure 3.**
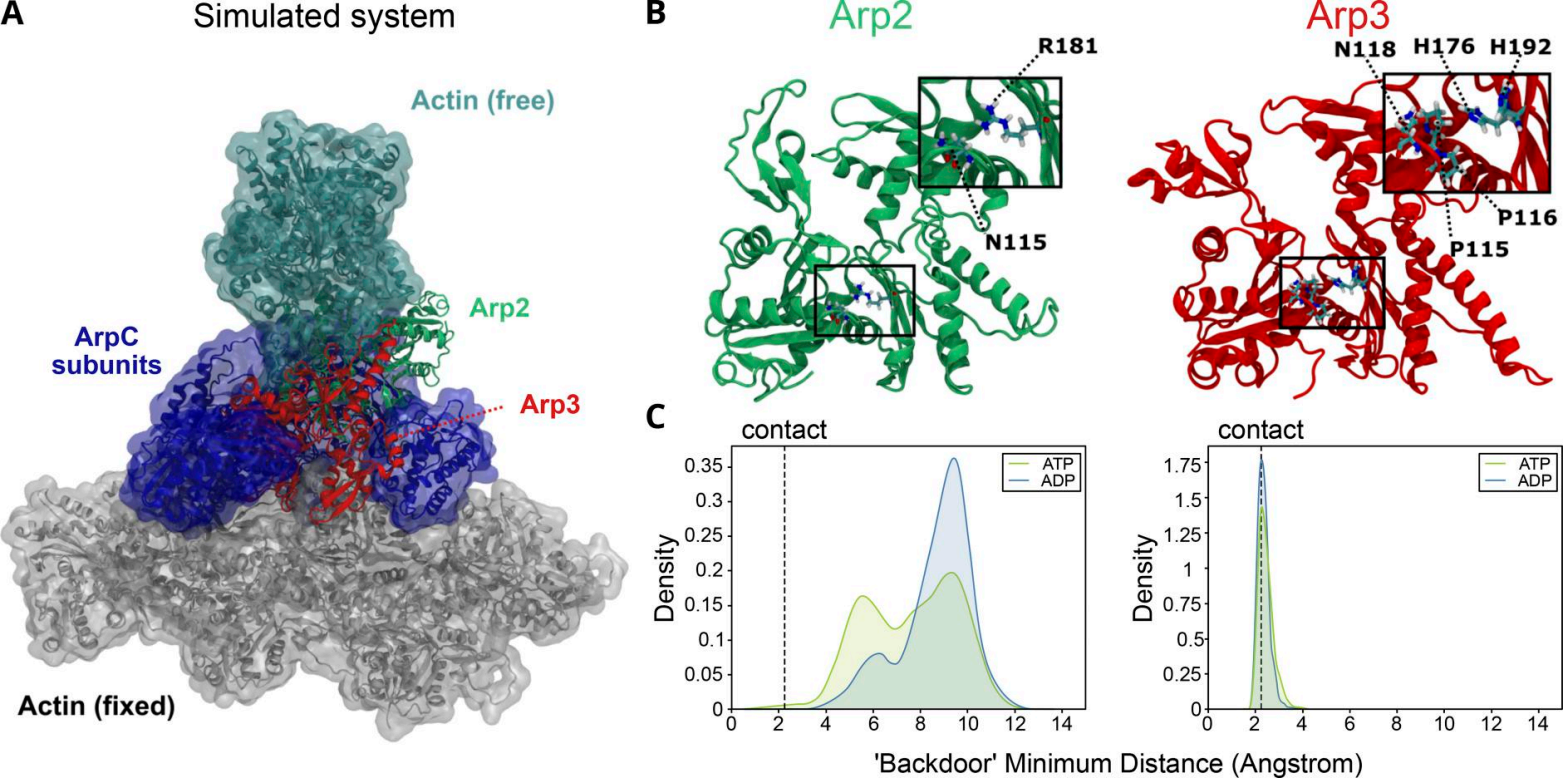
**Putative backdoor for Pi release is open in Arp2, but closed in Arp3. (A)** Simulated system contains 6 actin subunits as the mother filament (gray), ArpC1 to ArpC5 (blue), Arp2 (green), Arp3 (red), and the 2 first actin subunits of the daughter filament (green). Actin, Arp2, and Arp3 subunits were modeled in the ADP state (see Materials and methods; PDB 7TPT). Actin subunits from the mother filament are fixed, while the two actin subunits of the daughter filament are free to evolve during the molecular dynamics simulation. **(B)** Putative Arp2 and Arp3 backdoors shown on the protein structures (from PDB 7TPT), with highlights of key amino acids involved in the open/closed state of the backdoors. **(C)** Distribution of the minimum distance between loops 113–117 and 179–183 in Arp2, and 116–120 and 190–194 in Arp3, on the last 300 ns of all 3 repeats, revealed a much wider opening of the putative Pi release backdoor in Arp2 than in Arp3.

We next sought to assess whether the putative Pi backdoors in Arp2 and Arp3 were in open or closed states, for Arp2/3 complexes at branch junctions. These putative backdoors correspond to the main backdoor N111-R177 in mammalian actin (Oosterheert et al., 2023; Schahl et al., 2025). We performed 1-μs all-atom molecular dynamics simulations of the branch junction (see Materials and methods), with the Arp2/3 complex in the ADP state, 6 ADP–actin subunits for the mother filament, and 2 ADP–actin subunits for the daughter filament (Fig. 3 A). Because the sequences between mammalian actin, Arp2, and Arp3 are quite divergent, we measured the minimal distance observed between residues 113–117 and 179–183 in Arp2, and residues 116–120 and 190–194 in Arp3 to assess whether these putative backdoors in Arp2 and Arp3 are open or closed (Fig. 3 B). Throughout the course of the simulations, we observed that only the backdoor in Arp2 appeared to dynamically widen, while the one in Arp3 remained closed. The presence of 2 successive prolines in Arp3, P115 and P116, rigidifies the loop. It thus seems that the nucleotide pocket of ADP-Arp2, but not the one of ADP-Arp3, is accessible for Pi to shuttle in and out. Therefore, the variation of the debranching rate to the presence of Pi in solution (Fig. 2 C) would result only from the saturation of a single binding site within the Arp2/3 complex at the branch junction. To see whether the presence of the gamma-phosphate in the nucleotide pocket would change the opening of these putative backdoors, we also performed simulations with ATP inside Arp2 and Arp3 at branch junctions. We observed distributions of the minimum distances of the backdoors similar to the ones in the ADP state (Fig. 2 C). This illustrates the ability of the backdoor of Arp2 to remain open, even in the presence of the third phosphate within the nucleotide-binding site.

Taken together, our data reveal that ADP- and ADP-Pi-Arp2/3 branch junctions have debranching rates with similar apparent exponential responses to force. However, the presence of Pi, presumably inside Arp2, strongly stabilizes branch junctions, in the absence of force and over a broad range of pulling forces.

### Branch renucleation depends on force and on the nucleotide state of the Arp2/3 complex

We next wondered how the relative stability of the two interfaces of the Arp2/3 complex at the branch junction could vary as a function of the applied pulling force and the nucleotide state of the complex. Depending on which interface ruptures first and causes the branch to dissociate from the mother filament, the Arp2/3 complex can either remain on the mother filament and potentially regrow a branch, or dissociate from the mother filament and leave with the branch.

To gain insights into interface stability, we needed to combine the information obtained from debranching experiments with the information from renucleation experiments, as a function of force. As a simple model, each Arp2/3 complex interface, either with the mother filament or with the daughter filament, ruptures with a rate that varies with the applied force according to the transition state theory for a “slip” bond (Dudko et al., 2008), following the Bell–Evans relation: k_off_(F) = k_off_,_F=0_ ⋅ exp(F⋅Δx/k_B_T), where Δx is the distance between the bound and transition states along the reaction coordinate, and k_B_⋅T is the thermal energy (4.1 pN.nm at 25°C). The debranching rate is the sum of the dissociation rate of the two interfaces, k_deb_(F) = k_off_^daughter^(F) + k_off_^mother^(F). The probability to renucleate a branch is given by the probability that the Arp2/3 complex–daughter filament interface ruptures first, which can be written as k_off_^daughter^(F)/(k_off_^daughter^(F) + k_off_^mother^(F)), under saturating actin and ATP concentrations (as previously established in Ghasemi et al., 2024).

**Figure 4.**
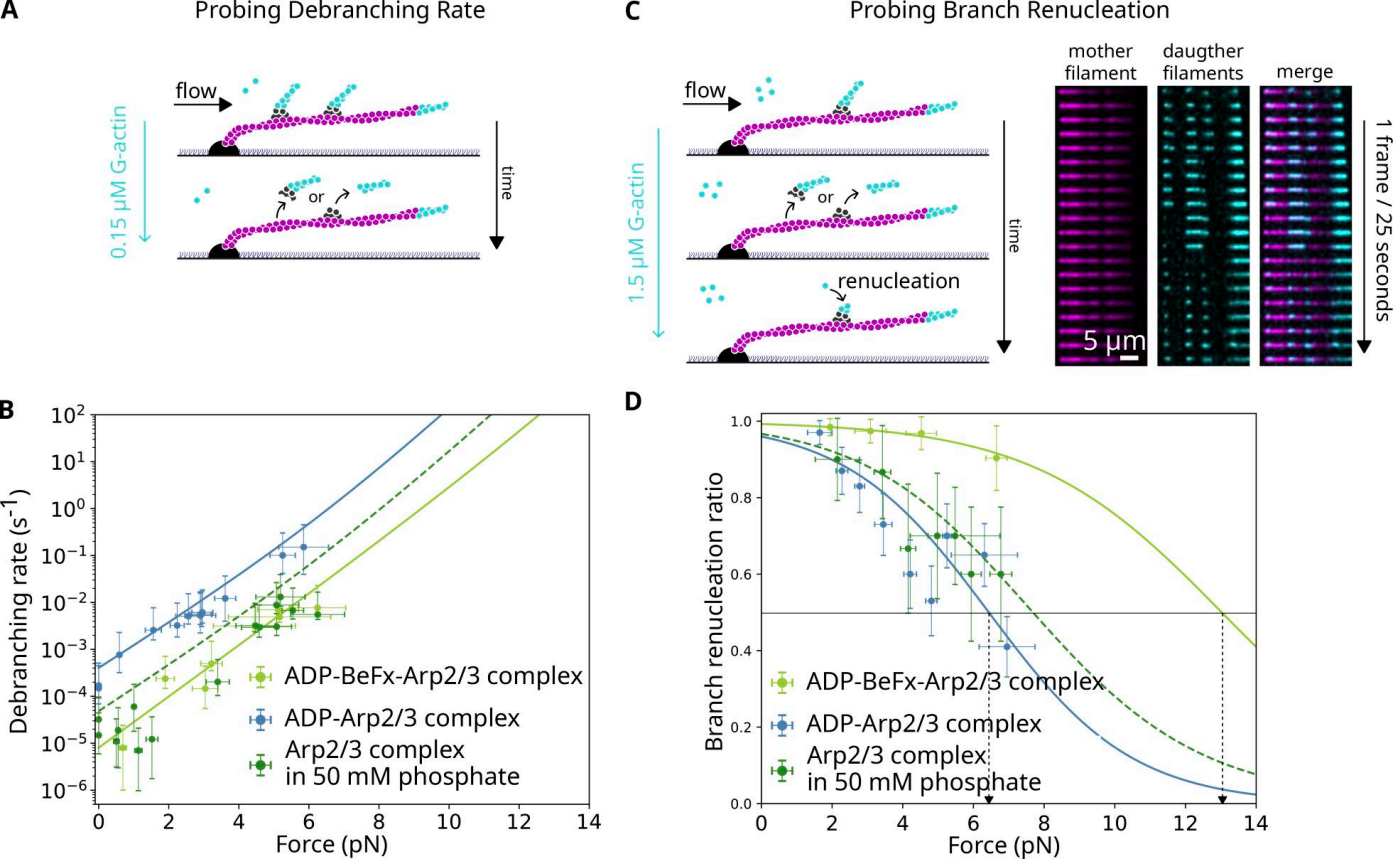
**Stability of the Arp2/3 complex interfaces is nucleotide-dependent. (A)** Schematics of the experiment where the debranching rate is probed at different pulling forces in the presence of 0.15 µM actin. **(B)** Debranching rate as a function of the pulling force for ADP (blue)- and ADP-BeFx (light green)-Arp2/3 complex branch junctions, and for Arp2/3 complex branch junctions exposed to 50 mM phosphate buffer (dark green, dashed). Each point is from a single experiment with at least 40 analyzed branches. The error bar for each individual data point is the standard deviation of the force. All data points are from Figs. 1 and 2. **(C)** (Left) Schematics of the experiments where the branch renucleation ratio is probed at different pulling forces, in the presence of 1.5 µM Alexa Fluor 568 (10%)–G-actin. (Right) Time-lapse images showing the branch renucleation from ADP-BeFx Arp2/3 complexes, with the actin mother filament in magenta, and actin branches in cyan. The scale bar is 5 µm. **(D)** Branch renucleation ratio as a function of the pulling force for ADP (blue)- or ADP-BeFx (light green)-Arp2/3 complexes, and for Arp2/3 complexes exposed to 50 mM phosphate (dark green, dashed). Each point is from a single experiment with at least 30 analyzed branches. The error bar for each individual data point is the standard deviation of the force, and the 95% confidence interval for the branch renucleation ratio. In B and D, continuous lines are best fits of the data, obtained by the simultaneous least-square error minimization of the debranching rate and of the branch renucleation ratio, for each Arp2/3 complex nucleotide state (see the main text). For the experiments performed in the presence of 50 mM phosphate, the dashed lines are directly obtained by considering branch junctions that detach 68% of the time with their Arp2/3 complex in the ADP state, and 32% in the ADP-Pi state that do not interconvert, and using the parameter obtained from the fits of the ADP- and ADP-BeFx-Arp2/3 complex branch junction data (see Materials and methods).

**Figure S4.**
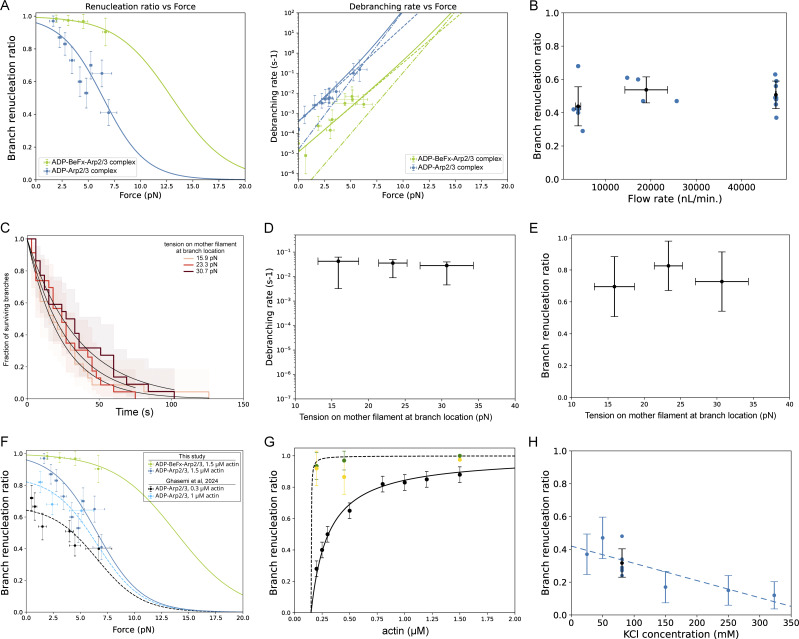
**Simultaneous fit of debranching rate and branch renucleation ratio. (A)** Same data and fit as in Fig. 4, C and D, displayed from 0 to 20 pN. The dashed lines indicate the contribution to the overall debranching rate of the Arp2/3 complex interface with the daughter filament, while the dash-dot lines the one with the mother filament. **(B)** Branch renucleation ratio does not depend on the flow rate on surviving Arp2/3 complexes. Branch renucleation ratio from ADP-Arp2/3 (average pulling force on branch junctions is 3.8 (±0.9) pN) force as a function of applied flow rate in the microfluidics chamber (*n* = 26, 53, 20, 23, 22, 26, 21, 99, 60, 60, 91, 35, 81, 36, 88, 35, 81, 29, and 89 branches in flow rate ascending order), in the presence of 0.5 µM actin in F-buffer. Black data points are average values from individual data points binned by flow rates, with standard deviation error bars. Pairwise Welch’s tests result in *P* values >0.13. **(C)** Tension exerted on mother filaments at the branch location has no impact on the debranching rate, and on the branch renucleation ratio for ADP-Arp2/3 complex branches. Surviving fractions of branches monitored over time for branches grouped according to the tension experienced by the mother filament at the branch junction (*n* = 23, 23, and 22 branches for an average tension on the mother filament at the branching point of 15.9, 23.3, and 30.8 pN, respectively). All daughter filaments are exposed to the same flow rate, are pulled with an average force of 4.1 pN, and are exposed to 1.5 µM Alexa Fluor 568 (10%)–G-ATP-actin. Shaded areas are 95% confidence intervals. Black curves are single-exponential fits. **(D)** Debranching rates as a function of the tension experienced by the mother filament at the branching point, obtained from single-exponential fits of the data in C. Pairwise logrank tests performed between the survival fractions of the three populations show no statistically significant difference (all *P* values >0.05). **(E)** Branch renucleation ratio as a function of the tension experienced by the mother filament at the branch point, from the three groups of branches used in C. **(F)** Branch renucleation ratio follows the same decaying trend at different actin concentrations for ADP-Arp2/3 complexes. Comparison of the branch renucleation ratio for different pulling forces for ADP (blue)- or ADP-BeFx (green)-Arp2/3 complex branch junctions, in the presence of the indicated concentration of G-actin and 200 µM ATP. Data at 0.3 and 1 µM actin for ADP-Arp2/3 complex branch junctions are from Ghasemi et al. (2024). Data at 1.5 µM actin are from Figure 4. For ADP-Arp2/3 complex branches, each point is from a single experiment with at least 30 analyzed branches. Continuous lines are the best fits of the branch renucleation ratio from Fig. 3. Dashed lines are the best fits of the branch renucleation ratio data at 0.3 and 1 µM with a rescaling factor compared with the fitted curve of the 1.5 µM experimental data, with the rescaling factor as the only free parameter. The branch renucleation ratio depends on the actin concentration as follows: $\frac{0.97.k_{on}.\left(\left[actin\right]-C_{c}\right)}{k_{on}.\left(\left[actin\right]-C_{c}\right)+k_{off}^{ATP-Arp2/3}}$ (Ghasemi et al., 2024). The renucleation factors estimated from the fits would give an estimate of the actin concentration of 0.6(±0.05) and 0.9(±0.2) µM for the experiment performed at 0.3 and 1 µM, respectively, in Ghasemi et al. (2024). **(G)** Branch renucleation at different actin concentrations. Branch renucleation ratio from ADP-BeFx-Arp2/3 branches (green) in the presence of 0.2 (*n* = 31 branches), 0.45 (*n* = 32 branches), 1.5 µM actin (*n* = 32 branches), 200 µM ATP, with an average pulling force of 2 pN. Branch renucleation ratio from ADP-Arp2/3 complex branch junctions exposed to 20 nM cortactin (gold), in the presence of 0.2 (*n* = 25 branches), 0.45 (*n* = 31 branches), 1.5 µM actin (*n* = 40 branches), 200 µM ATP, with an average pulling force of 2.2 pN. Black line and data points are ADP-Arp2/3 complex branch junctions without cortactin from Ghasemi et al. (2024), and were obtained for 1 pN pulling force. The dashed line corresponds to the theoretical renucleation ratio from the ADP-BeFx-Arp2/3 complex, with an actin binding rate to the barbed end of Arp2/3 assumed to be similar to the one determined for the ADP-Arp2/3 complex, k_on_ = 3.4 µM^−1^.s^−1^ (Ghasemi et al., 2024), in competition with the spontaneous dissociation of surviving ADP-BeFx-Arp2/3 complexes from mother filaments occurring at a rate of 4.6 10^−3^ s^−1^ (Fig. 5). **(H)** Impact of ionic strength on branch renucleation ratio. Branch renucleation ratio from ADP-Arp2/3 branches in the presence of 0.5 µM actin and 200 µM ATP, with an average pulling force of 4.2 pN, for different KCl concentrations in the regular buffer. Individual data points are from single experiments (*n* > 30 branches for each experiment). The black data point at 81 mM KCl is the average from six experiments. Error bars are standard deviations. The dashed line indicates the linear regression of the individual data points.

First, we conducted branch renucleation experiments for ADP-Arp2/3 complexes at different pulling forces, in conditions where nearly all Arp2/3 complexes that remain bound to the mother filament after branch dissociation would nucleate a new branch (i.e., in the presence of 1.5 µM actin and 200 µM ATP [Ghasemi et al., 2024]). In these experiments, the applied pulling force is increasing over time as branches are elongating due to the presence of a substantial concentration of actin. The branch renucleation ratio was thus quantified as a function of the average force at which the population of branches detached from the mother filament. The simultaneous fit of the debranching rate and the renucleation ratio measured experimentally from these two sets of experiments indicated that at zero force, the ADP-Arp2/3 complex–mother filament interface is ∼20 times more stable than the ADP-Arp2/3 complex–daughter filament interface (Fig. 4). This difference in interface stability diminishes with increasing force. Above ∼ 6.5 (±0.8) pN, the Arp2/3 complex–mother interface becomes less stable than the interface with the daughter filament, and the majority of Arp2/3 complexes now detach from the mother filament and leave with the daughter filament upon debranching (Fig. S4 A and Table S1). It was previously reported that branch formation was favored on the convex face of the curved filament (Risca et al., 2012). Applying tension on the mother filament would reduce local curvature fluctuations in our microfluidics assay. We observed that the tension on the mother filament did not impact debranching rate nor branch renucleation ratio (Fig. S4, B–E), indicating that the tensile state of the mother filament does not significantly modify the way the Arp2/3 complex interacts with the mother filament.

We next performed branch renucleation experiments with ADP-BeFx-Arp2/3 complex branch junctions (Fig. 4). Strikingly, we observed that the renucleation ratio for ADP-BeFx-Arp2/3 complexes remains high (>90%) for forces up to 6.5 pN, a range of forces much larger than for ADP-Arp2/3 complex branch junctions. According to the fit using the “two-interface” model, at zero force, the ADP-BeFx-Arp2/3 complex–mother filament interface appears to be roughly 100 times more stable than the daughter filament interface, and becomes less stable than the daughter filament interface only above ∼ 13 (±1.5) pN, a force twice higher than for ADP-Arp2/3 complexes. This indicates that the presence of BeFx in Arp2/3 complexes reinforces both interfaces, and the interface with the mother filament more than the one with the daughter filament.

In the presence of 50 mM phosphate, as mentioned earlier, 68% of the debranching events occur with Arp2/3 complexes in the ADP state. When conducting branch renucleation experiments in a 50 mM phosphate buffer, we indeed observed a force response of the branch renucleation ratio compatible with the expected behavior of a mixed population (68% ADP- and 32% ADP-Pi-Arp2/3 complexes) (Fig. 4 D).

**Figure S5.**
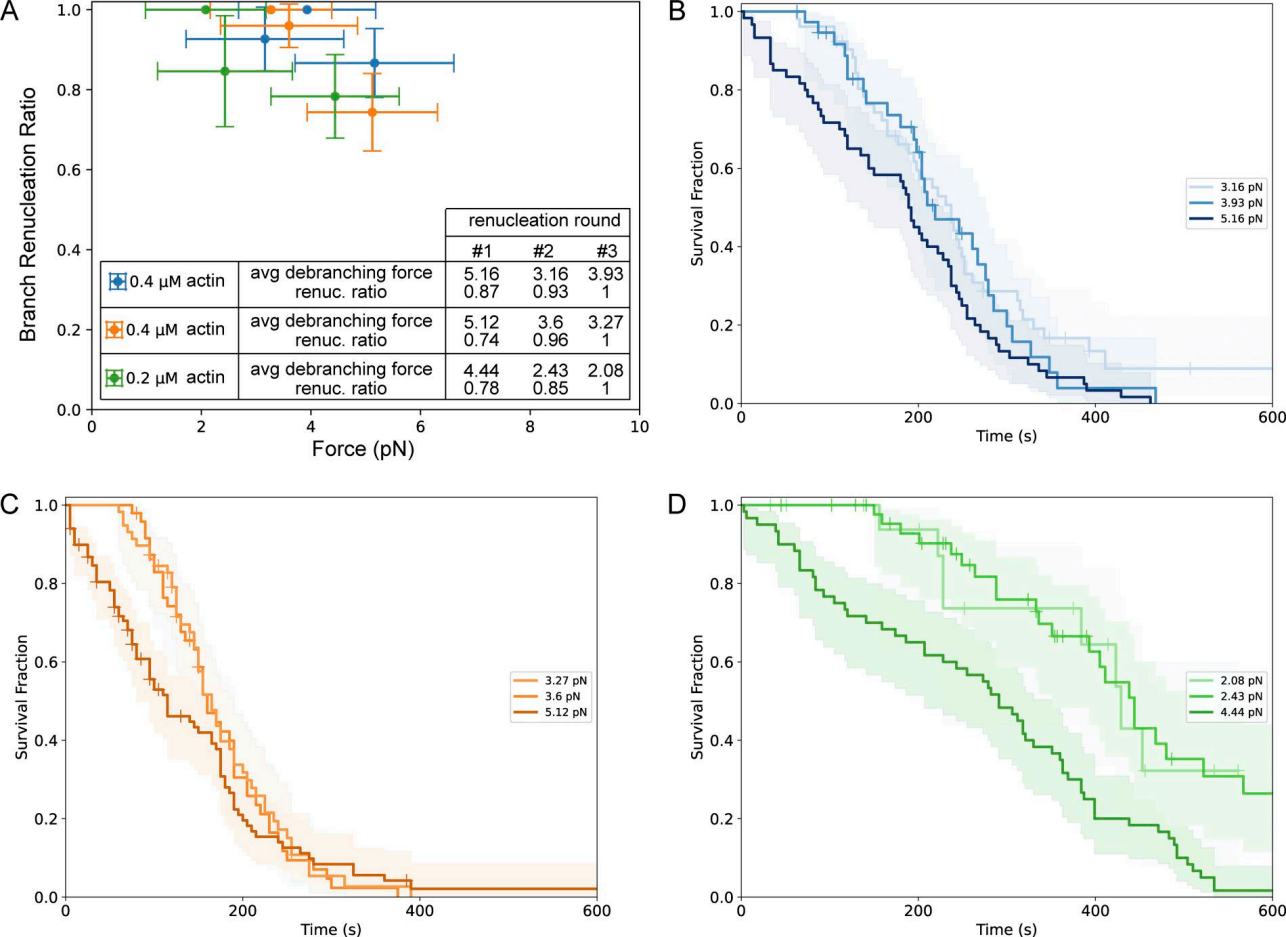
**ADP-BeFx-Arp2/3 complex renucleates for several rounds with a high renucleation ratio. (A)** Branch renucleation ratio as a function of force from Arp2/3 complex branches in the ADP-BeFx state. Renucleation is probed in the presence of indicated concentrations of actin, in the absence of BeFx in solution. **(B–D)** Branch survival fractions from the same initial population of Arp2/3 complexes attached to mother filaments for different forces and actin concentrations. Actin concentration color code is the same as in the top panel, the darkest tone is the first round of branches, the lightest the third round (number of branches per conditions: green, 0.4 µM actin: 60 [5.16 pN], 41 [3.16 pN], 29 [3.93 pN]; orange, 0.4 µM actin: 78 [5.12 pN], 50 [3.6 pN], 44 [3.27 pN]; blue, 0.2 µM actin: 60 [4.44 pN], 36 [2.43 pN], 9 [2.08 pN]).

We also observed that the renucleation ratio of ADP-BeFx-Arp2/3 complex branch junctions remained high for several generations of renucleated branches in the absence of BeFx in solution, even at low actin concentration (Fig. S5). This indicates that Arp2/3 complexes remained in the ADP-BeFx state for several rounds of renucleation. Surviving ADP-BeFx-Arp2/3 complexes can thus nucleate new branches without the need to reload ATP. This additionally indicates that surviving ADP-BeFx-Arp2/3 complexes dissociate slowly from mother filaments, giving enough time to nucleate a new branch when actin concentration is low.

**Figure 5.**
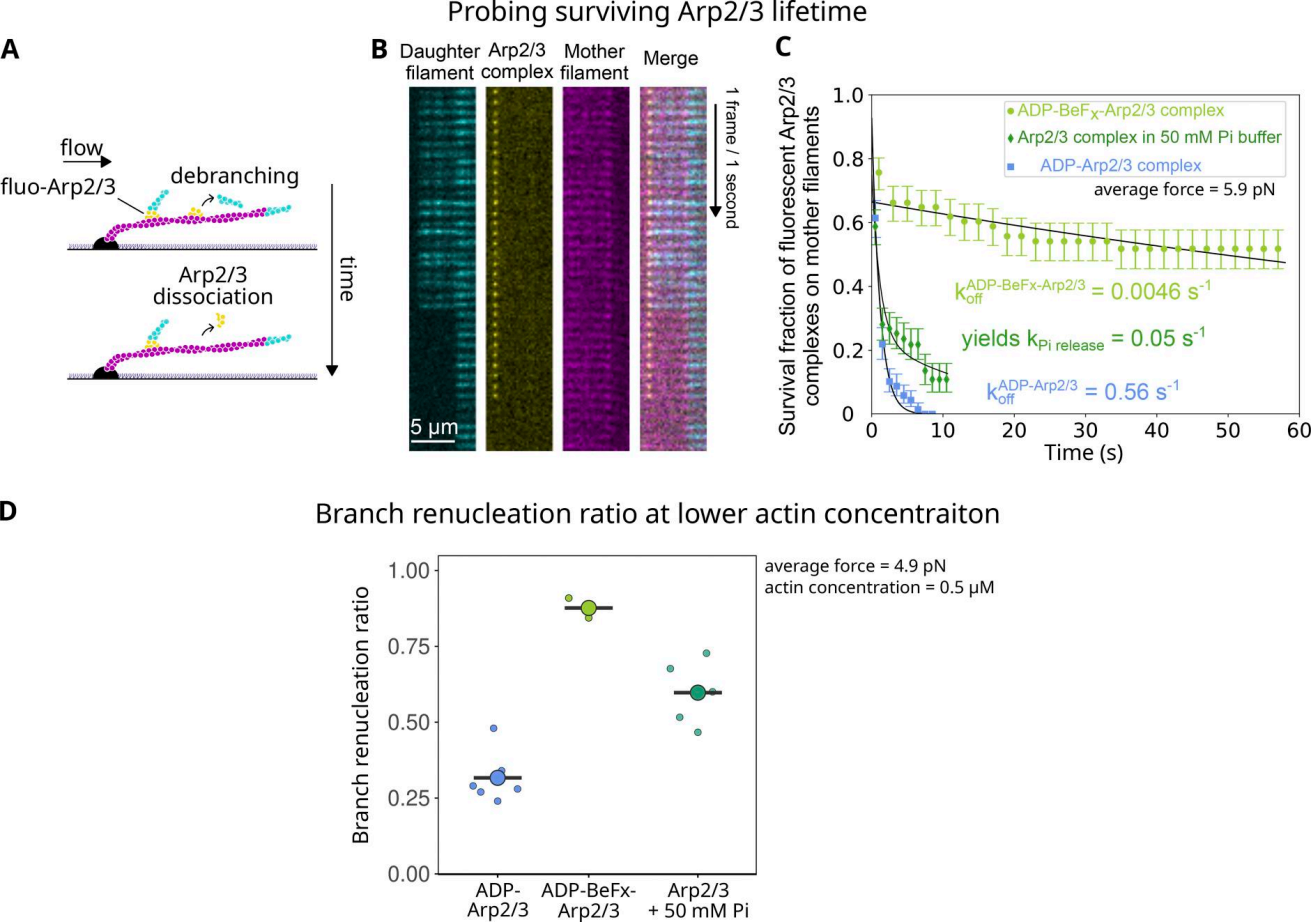
**Arp2/3 complex nucleotide state tunes its stability and its ability to renucleate branches. (A)** Schematic of the debranching experiment with fluorescently labeled Arp2/3 complexes. Previously formed branches are exposed to a buffer containing 200 µM ATP, without actin in order to observe the dissociation of fluorescent Arp2/3. Images are acquired every second for ADP-Arp2/3 branches in a regular buffer or exposed to a 50 mM Pi buffer, and every 2 s for ADP-BeFx-Arp2/3 complex branches. **(B)** Time-lapse images of an experiment using Alexa Fluor 488–labeled Arp2/3 complex in 50 mM Pi buffer. **(C)** Fraction of surviving fluorescently labeled Arp2/3 complex still bound to the mother filament as a function of time, observed as described in A, pooled from two experiments for each condition, in the ADP (*n* = 70 branches, blue) or ADP-BeFx (*n* = 74 branches, light green) nucleotide state, or in the presence of 50 mM phosphate (*n* = 80 branches, dark green). For all experiments, branches are pulled on with an average force of 5.9 pN, and the ionic strength of the buffer is adjusted to match the one of the 50 mM phosphate buffer. Fits (black lines) by a monoexponential decay function yield k_off_^ADP^ = 0.56 (±0.08) s^−1^ and k_off_^ADP-BeFx^ = 4.6 (±0.7) × 10^−3^ s^−1^ for ADP and ADP-BeFx Arp2/3 complex experiments, respectively. For the 50 mM phosphate buffer condition, the fit using a model explained in the main text and the Method section yields k_Pi rel._ = 0.057 (±0.03) s^−1^. Error bars indicate 95% confidence intervals. **(D)** Branch renucleation ratio measured for Arp2/3 complex branch junctions in ADP, ADP-BeFx states, or in the presence of 50 mM Pi, in the presence of 0.5 μM G-actin in solution and an average pulling force of 4.9 pN. Each small data point indicates a single experiment with at least 30 analyzed branches. Large data points are averages for each condition.

**Figure S6.**
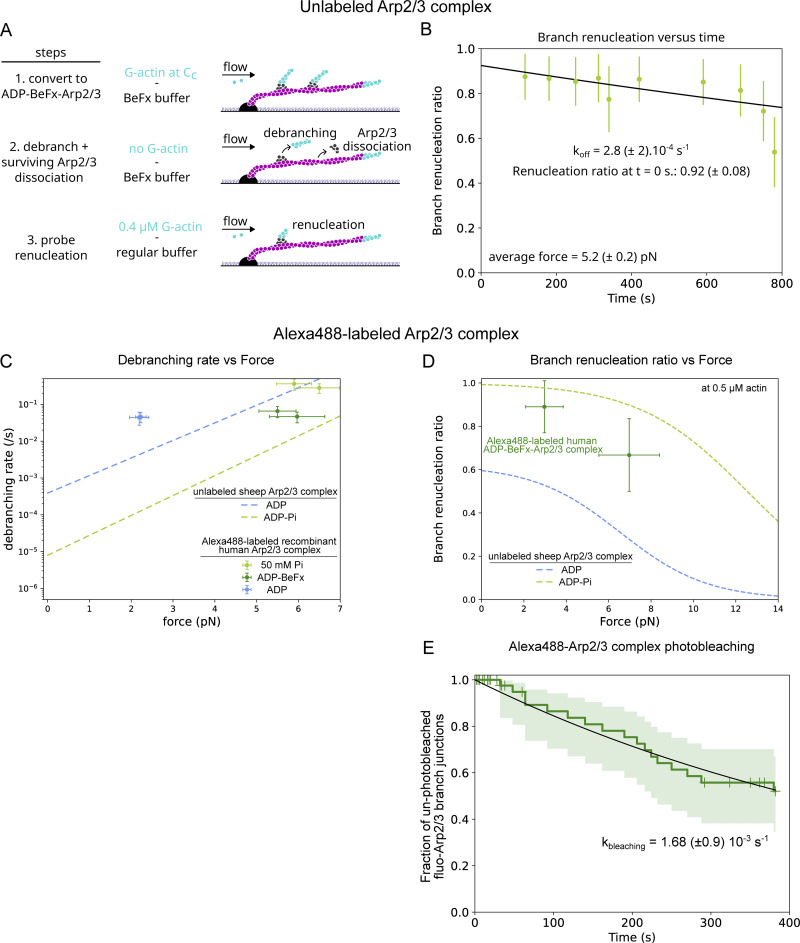
**Surviving ADP-BeFx-Arp2/3 complexes detach slowly from mother filaments. (A)** Schematics of the renucleation experiment for unlabeled ADP-BeFx-Arp2/3 complex branch junctions. Branches are first nucleated and converted to BeFx for 6 min as in Fig. 1 C, then exposed to BeFx buffer with no G-actin for 6 (*n* = 150 branches) or 13 (*n* = 261 branches) minutes, before being exposed again to a regular buffer solution containing 0.4 µM Alexa Fluor 568 (10%)–G-ATP-actin (cyan) to allow branches to regrow. During the debranching step, the debranching time for each branch is recorded. In case of branch regrowth, the time interval between this debranching event and re-exposure to G-actin is calculated to assess the branch renucleation ratio versus time of ADP-BeFx-Arp2/3 complex branches. **(B)** Branch renucleation ratio for ADP-BeFx-Arp2/3 complex branch junctions, probed as shown in A, pulling with an average force of 5.2 pN. Each data point represents a branch renucleation ratio computed on at least 40 branches. Error bars are standard deviations. Exponential fit of the data gives a rate of detachment of the surviving ADP-BeFx-Arp2/3 complex from the mother filament of 2.8 (±2) 10^−4^ s^−1^, and a fraction of Arp2/3 complexes remaining bound to mother filaments upon branch dissociation of 0.92 (±0.08). **(C)** Debranching rates as a function of constant pulling forces (log-linear representation), for ADP (blue)- and ADP-BeFx (green)–Alexa 488–labeled human Arp2/3 complex branch junctions, or in the presence of 50 mM Pi (light green). Error bars are standard deviations for the force, and confidence intervals on the single-exponential fits, obtained by fitting the upper and lower bounds of the 95% confidence intervals of the survival fractions from debranching experiments. Data are obtained from experiments with at least 28 analyzed branches for each condition. As a comparison, dashed lines represent the behavior of the unlabeled sheep Arp2/3 complex, as characterized in Figs. 1, 2, 3, and 4 in the main text. **(D)** Branch renucleation ratio as a function of the pulling force for Alexa 488–labeled human ADP-BeFx-Arp2/3 complexes (green), probed at 0.5 µM actin. Data points are from single experiments with 30 analyzed branches. The error bar for each individual data point is the standard deviation of the force, and the 95% confidence interval for the branch renucleation ratio. As a comparison, dashed lines represent the estimated branch renucleation ratio for unlabeled sheep Arp2/3 complexes at 0.5 µM actin. **(E)** Surviving fractions of unphotobleached Alexa 488–labeled Arp2/3 complex branch junctions over time, probed at an average pulling force of 1 pN, with images acquired every 2 s, obtained from 50 analyzed fluo-Arp2/3 complex branch junctions. Single-exponential fit (black curve) yields a photobleaching rate k_photobleach_ = 1.68 10^−3^ s^−1^. The shaded area represents the 95% confidence interval. Vertical ticks on the surviving curves are times where censoring events occurred.

To gain further insight into the stability of surviving Arp2/3 complexes on mother filaments, we measured their dissociation rate in the different nucleotide states. We performed debranching experiments using Arp2/3 complexes fluorescently labeled with Alexa Fluor 488 (see Materials and methods). We recorded the dwell time of Arp2/3 complexes after branch dissociation in the absence of actin in a buffer containing 200 µM ATP (Fig. 5, A–C). Surviving ADP-Arp2/3 complexes quickly reload ATP (see Fig. 5 E and Ghasemi et al. [2024]) and dissociate at a rate k_off_^ATP-Arp2/3^ = 0.56 s^−1^, similar to what we previously established (Ghasemi et al., 2024). Surviving ADP-BeFx-Arp2/3 complexes dissociated from mother filaments at a rate k_off_^ADP-BeFx-Arp2/3^ = 4.6 × 10^−3^ s^−1^, >100 times slower than ADP-Arp2/3 complexes. We confirmed this stability using unlabeled ADP-BeFx-Arp2/3 complexes, by debranching in the absence of actin for several minutes before re-exposing mother filaments to actin in order to reveal, as they renucleated branches, the surviving ADP-BeFx-Arp2/3 complexes that were still bound to mother filaments (Fig. S6). In the presence of 50 mM phosphate, branches dissociate from Arp2/3 complexes either in the ADP (68%) or in the ADP-Pi (32%) state. In the presence of ATP, we previously showed that the surviving ADP-Arp2/3 complex quickly releases ADP, with a release rate of at least 21 s^−1^ (Ghasemi et al. [2024]; see Materials and methods). We hypothesize that the residency of ADP is so unfavorable that ADP release is much faster than the binding of Pi to the surviving Arp2/3 complex. Thus, we consider that even in the presence of 50 mM Pi, ADP release and ATP reloading occur faster than Pi rebinding, such that surviving ADP-Arp2/3 complexes quickly reload ATP and then dissociate from mother filaments at the rate of 0.56 s^−1^, as above. In contrast, surviving ADP-Pi-Arp2/3 complexes either dissociate in the ADP-Pi state, or release their Pi and rapidly reload ATP, and then dissociate from mother filaments. Based on this reaction scheme (see Fig. 8 A), we computed the fraction of surviving fluorescent Arp2/3 complexes still bound to the mother filament (see Materials and methods) and fitted the data, yielding a Pi release rate from surviving ADP-Pi-Arp2/3 complexes of 0.057 (±0.03) s^−1^ (Fig. 5 C), around 4 times lower than what we observed for Pi release from Arp2/3 complexes at branch junctions (Fig. 2 F).

Both the slow spontaneous dissociation of surviving Arp2/3 complexes in the ADP-Pi state and the slow Pi release give ample time for actin monomers to bind to surviving ADP-Pi-Arp2/3 complexes and regrow a branch, and their renucleation efficiency should thus be comparable to that of ADP-BeFx-Arp2/3 complexes. To illustrate this, we conducted experiments in the presence of a low actin concentration (0.5 µM), where the branch renucleation ratio for ADP-Arp2/3 complexes is low (35%, Fig. 5 D). We measured a higher renucleation ratio in the presence of 50 mM Pi compared with what we observed for ADP-Arp2/3 complexes in a regular buffer (60% versus 35%, Fig. 5 D). This is consistent with our other measurements because in the presence of 50 mM Pi, 32% of the surviving Arp2/3 complexes are in the ADP-Pi state and renucleate a branch with ∼ 90% efficiency, as measured for ADP-BeFx-Arp2/3 complexes (Fig. 5 D and Fig. S4 G).

We have summarized all the reaction routes from the branch nucleation to detachment and renucleation, with quantitative values for the rates measured in this study, in Fig. 8 A.

### GMF destabilizes the ADP-Arp2/3–daughter interface

GMF inhibits Arp2/3 nucleation and directly promotes debranching (Ghasemi et al., 2024; Koundinya et al., 2025; McGuirk et al., 2024; Ydenberg et al., 2013). However, the molecular details of how GMF favors debranching and prevents branch renucleation remain unclear. We thus explored how pulling forces and the nucleotide state of the Arp2/3 complex could impact human GMFγ (hereafter referred to as “GMF”) activity.

**Figure 6.**
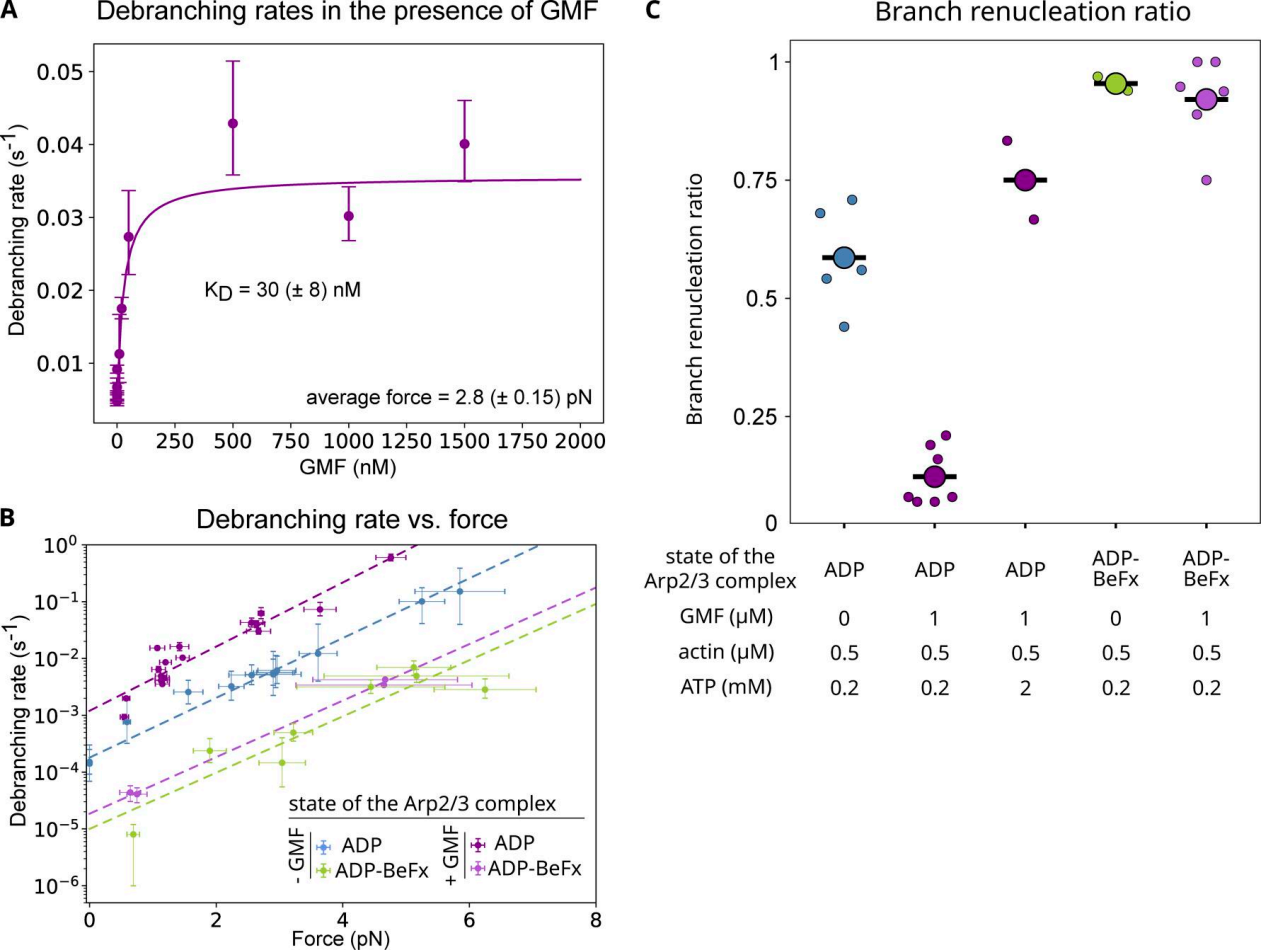
**GMF accelerates debranching in a nucleotide-dependent manner. (A)** Debranching rate of ADP-Arp2/3 complex branch junctions exposed to various concentrations of GMF, from experiments performed at a constant average pulling force of 2.8 pN, in the presence of 0.15 µM actin and 200 µM ATP. Each data point is from a single experiment with at least 30 analyzed branches. Error bars are confidence intervals on the single-exponential fits obtained by fitting the upper and lower bounds of 95% confidence intervals of the survival fractions of debranching experiments. The binding affinity of GMF for ADP-Arp2/3 complexes at branch junctions is 30 (±8) nM, as derived from the fitting of the saturation curve. **(B)** Debranching rate of ADP- or ADP-BeFx-Arp2/3 complex branch junctions, in the presence or absence of at least 500 nM GMF in solution, for different pulling forces (log-linear representation). Dashed lines are single-exponential fits of the data points for each condition. Error bars are standard deviations for the force, and confidence intervals on the single-exponential fits, obtained by fitting the upper and lower bounds of 95% confidence intervals of the survival fractions of debranching experiments. Each data point is from a single experiment with at least 19 and 50 analyzed branches for ADP- and ADP-BeFx-Arp2/3 complex branch junctions in the presence of GMF, respectively. Data in the absence of GMF are from Fig. 3. To facilitate the comparison of the data, single-exponential fits are represented (dashed lines). **(C)** Branch renucleation ratio for ADP- or ADP-BeFx-Arp2/3 complex branch junctions exposed to an average pulling force of 2.5 pN, in the presence or absence of 0.5 µM actin and 1 µM GMF, and either 200 µM or 2 mM ATP in solution. Small data points are from single experiments with at least 30 analyzed branches. Large data points represent the average value for each condition (at least 2 replicates).

First, we exposed ADP-Arp2/3 complex branch junctions to varying concentrations of GMF. We observed an up to an approximately sixfold increase in the debranching rate, with a GMF affinity for the ADP-Arp2/3 complex at the branch junction of 30 (±8) nM (Fig. 6 A), consistent with previous studies using *Drosophila* GMF (Ghasemi et al., 2024) or yeast *S. pombe* GMF (Pandit et al., 2020), though with different affinity and amplitude for each species. The debranching rate of ADP-Arp2/3 complex branch junctions exposed to saturating amounts of GMF (>500 nM) increases in an exponential fashion with force, in a similar manner to without GMF (Fig. 6 B). Exposing ADP-BeFx-Arp2/3 complex branch junctions to a high concentration of GMF did not result in any appreciable increase in the debranching rate, at all forces tested (Fig. 6 B).

We next investigated the ability to renucleate a branch in the presence of GMF, as a proxy to reveal whether the Arp2/3 complex leaves with the branch upon debranching or remains attached to the mother filament. Previous attempts to measure the dwell time of surviving ADP-Arp2/3 complexes in the presence of GMF (Chung et al., 2022; Ghasemi et al., 2024) did not allow discriminating between these two possibilities. As observed with *Drosophila* GMF (Ghasemi et al., 2024), the branch renucleation ratio was very low in the presence of GMF compared with its value in the absence of GMF (Fig. 6 C). Unexpectedly, increasing the ATP concentration 10-fold, to 2 mM, was sufficient to restore the branch renucleation ratio to that of ADP-Arp2/3 complex branch junctions in the absence of GMF (Fig. 6 C). This observation suggests that ADP-Arp2/3 complexes remain bound to mother filaments after branch dissociation, but that GMF accelerates their subsequent departure. This accelerated departure of the surviving ADP-Arp2/3 complex can be outcompeted by increasing ATP concentration to reload ATP inside Arp2/3 complex faster, which allows branch regrowth. It is possible that GMF not only accelerates surviving ADP-Arp2/3 dissociation, but also partially inhibits the reloading of ATP from surviving Arp2/3 complexes (by either preventing the departure of the bound ADP or the binding of ATP). For ADP-BeFx-Arp2/3 complex branches, at 200 µM ATP, branch renucleation ratio was unaffected by GMF (Fig. 6 C). Thus, GMF is unable to destabilize ADP-BeFx-Arp2/3 complexes at branch junctions, and unable to accelerate the dissociation of the surviving ADP-BeFx-Arp2/3 complex from the mother filament after debranching. Note that our observations do not rule out the ability of GMF to bind to ADP-BeFx-Arp2/3 complexes, as GMF is able to bind to ATP-Arp2 of inactive Arp2/3 complexes in solution as evidenced by cryo-EM studies, albeit with low affinity (Luan and Nolen, 2013). Our data suggest that GMF has a limited impact on ADP-BeFx-Arp2/3, which could be due to the high stability of the filament-binding interfaces of Arp2/3 complexes in the ADP-BeFx state.

### Cortactin stabilizes Arp2/3 complex branch junctions in a force-dependent manner

**Figure 7.**
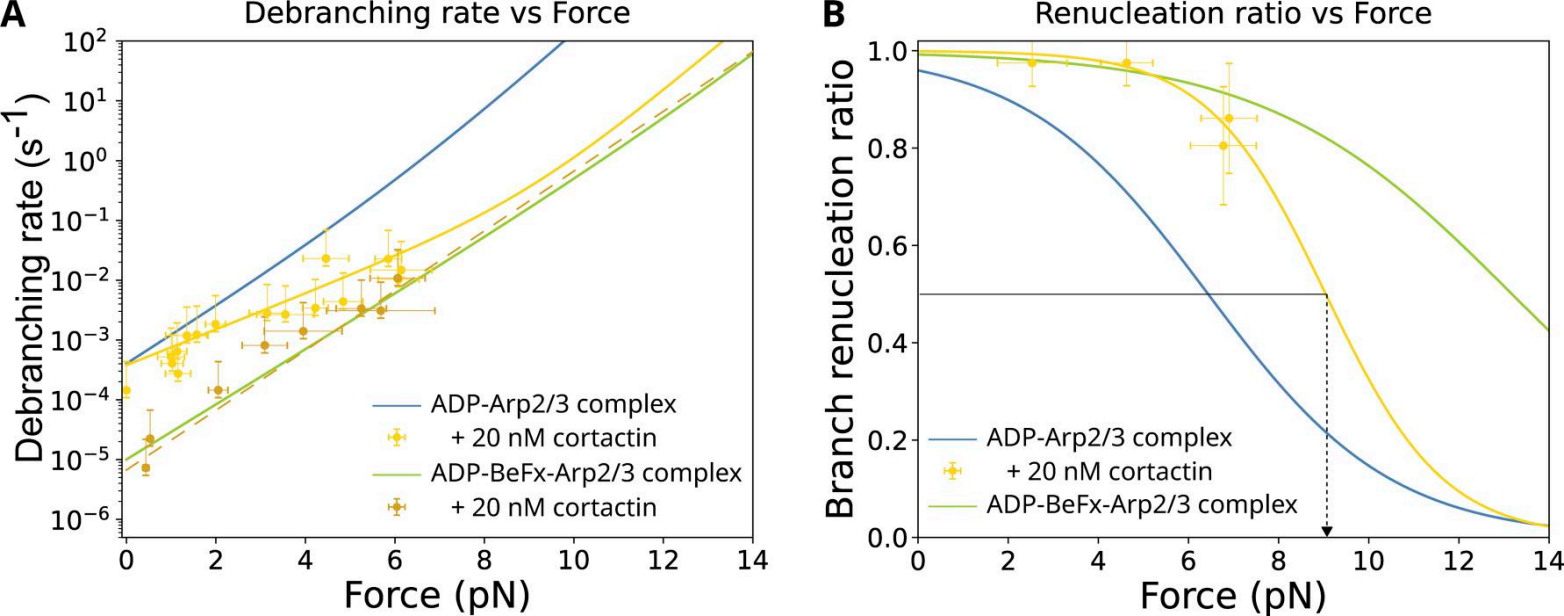
**Cortactin stabilizes ADP-Arp2/3 complex branch junctions and favors renucleation. (A)** Debranching rates for different pulling forces for ADP (yellow)- or ADP-BeFx (medium gold)-Arp2/3 complex branch junctions in the presence of 20 nM cortactin, and various actin concentrations. Each data point is from a single experiment with at least 60 and 37 analyzed branches for ADP- and ADP-BeFx-Arp2/3 complexes, respectively. Error bars are standard deviations. The dashed line for the ADP-BeFx-Arp2/3 complex in the presence of cortactin is a single-exponential fit from the data.** (B)** Branch renucleation ratio for different pulling forces for ADP-Arp2/3 complex branch junctions, in the presence (yellow) of 20 nM cortactin, with 1.5 μM G-actin and 0.2 mM ATP. Each point is from a single experiment from at least *n* = 30 branches. Error bars are standard deviations. In A and B, yellow lines are best fits of the data, obtained by the simultaneous error minimization of the probability of renucleating a branch and of the debranching rate, for ADP-Arp2/3 complex with cortactin, and debranching rates and renucleation ratios in the absence of cortactin are shown as lines, as in Fig. 3 (ADP (blue)- and ADP-BeFx (green)-Arp2/3 complex branches), for comparison.

We found that the presence of 20 nM cortactin reduces the debranching rate for the ADP-Arp2/3 complex in a force-dependent manner (Fig. 7 A). At low force (below around 2 pN), there is barely any detectable difference in the debranching rate with or without cortactin. At 6 pN, cortactin-bound ADP-Arp2/3 complex branches were ∼30 times more stable than without cortactin, reaching the stability of ADP-Pi-Arp2/3 complex branches without cortactin. Very surprisingly, the branch renucleation ratio of cortactin-bound ADP-Arp2/3 complex branch junctions remains high in the 0- to 6-pN force range (Fig. 7 B and Fig. S4 G). Although cortactin bridges the Arp2/3 complex and the daughter filament according to structural data (Liu et al., 2024), our observations indicate that this interface remains weaker than the interface with the mother filament, for all the pulling forces we probed. Cortactin shortens the distance between the energy minimum of the branch junction and the position of the energy barrier (“transition state”), making both interfaces more resistant to high pulling forces (Table S1).

We did not detect any appreciable impact of cortactin on the debranching rate of ADP-BeFx-Arp2/3 complex branch junctions at all forces (Fig. 7 A). Similar to GMF, we cannot rule out that cortactin binds to Arp2/3 complexes in the ADP-BeFx state, but it would then do so without providing extra stabilization.

## Discussion

**Figure 8.**
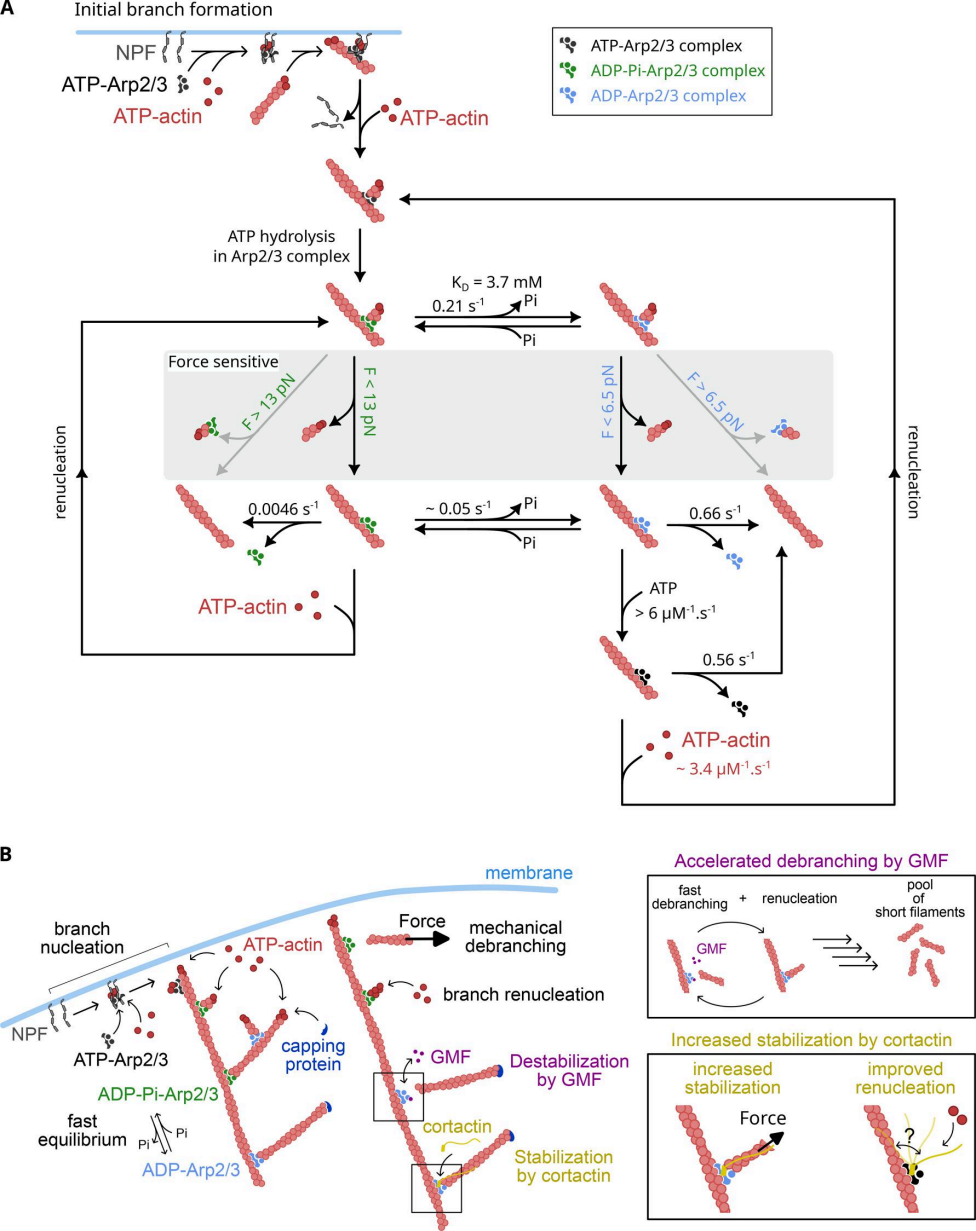
**Branch regulation by phosphate, mechanics, and regulatory proteins. (A)** Reaction scheme of the regulation of actin filament branches as a function of force and Pi in solution. After branch initiation by the coordinated action of membrane-bound NPFs, actin and inactive ATP-Arp2/3 complex, ATP-Arp2/3 quickly becomes ADP-Pi-Arp2/3. Depending on Pi concentration, branches are in rapid equilibrium between ADP- and ADP-Pi states. The gray box represents the force-sensitive stage. In the low pulling force regime (below 6.5 pN), for Arp2/3 complexes in either the ADP or ADP-Pi state, the interface with daughter filaments is more likely to rupture before the Arp2/3 complex–mother filament interface. Surviving ADP-Pi-Arp2/3 complexes detach very slowly from mother filaments. Pi release from the surviving ADP-Pi-Arp2/3 complex is slow enough to allow actin to bind to the Arp2/3 complexes and regrow a branch, without the need to exchange ADP for ATP in Arp2/3. The surviving ADP-Arp2/3 complex is more unstable, and rapidly reloads ATP to allow actin to bind to renucleate a branch where ATP in Arp2/3 complex will be hydrolyzed. Rate of the dissociation of surviving ADP-Arp2/3 complex from mother filaments is from previously published work (Ghasemi et al., 2024). **(B)** Actin filament branches are initiated by membrane-bound NPFs that activate Arp2/3, and recruit two actin monomers to the pseudo-barbed end formed by Arp2 and Arp3. Upon ATP hydrolysis within the Arp2/3 complex, the cytoplasmic Pi concentration sets the equilibrium between the ADP and ADP-Pi states of the Arp2/3 complex at branch junctions. Pulling force accelerates debranching. Capping proteins limit the growth of branches. Branch renucleation is favored by elevated concentrations of actin, ATP, or Pi in the cytoplasm. GMF and cortactin do affect ADP-Pi-Arp2/3 complex branch stability. GMF accelerates debranching and dissociation of surviving ADP-Arp2/3. Cortactin stabilizes branch junctions and favors renucleation from ADP-Arp2/3 complexes.

Here, using an *in vitro* microfluidics-based approach, we demonstrated that mammalian ADP-Pi-Arp2/3 complex branch junctions are around 30 times more stable than ADP-Arp2/3 complex branch junctions, even in the absence of force (see Fig. 8).

In the presence of Pi in solution, Arp2/3 complexes at branch junctions are in rapid equilibrium between ADP and ADP-Pi states. Mammalian actin subunits in filaments have an affinity for Pi (with a dissociation constant of 1.5 mM [Carlier and Pantaloni, 1988]) roughly similar to what we measured here for Arp2/3 complexes at branch junctions, but actin subunits release Pi nearly 100 times more slowly (Fujiwara et al., 2007; Jégou et al., 2013; Oosterheert et al., 2023; Schahl et al., 2025). Because of the slow actin Pi release rate, it is often said that actin filaments “age,” slowly transitioning over a few tens of seconds from being mostly composed of actin subunits in the ADP-Pi states, to a mixture of ADP-Pi/ADP subunits. In contrast, Arp2/3 complex branch junctions reach an equilibrium state, 50 times faster, in a couple of seconds. Importantly, in the presence of phosphate, Arp2/3 complex branch junctions quickly alternate between two conformations that have large differences in mechanical stability.

The debranching rate can be accelerated 200 times by applying a pulling force of 5 pN, a relevant force amplitude at the single actin filament level in cells. Irrespective of the nucleotide state of the Arp2/3 complex, at physiological ATP (∼ 1 mM) and actin (∼ 100 µM) concentrations, below 5-pN pulling force, the daughter filament detaches alone in most cases, and the surviving Arp2/3 complex that remains bound to the mother filament will regrow a branch.

In order to investigate in detail the ADP-Pi state of the Arp2/3 complex, we have taken advantage of ADP-BeFx, which is commonly referred to as an ADP-Pi analog for actin and myosin. Although still debated, it has been proposed that structurally, the ADP-BeFx state of F-actin is a mimic of the ATP state, based on the distance between the beta-phosphate and beryllium (Merino et al., 2018; Oosterheert et al., 2022). Our kinetic and mechanical measurements on branch junctions tend to suggest that ADP-BeFx is a good mimic of the ADP-Pi state for the Arp2/3 complex. However, one cannot exclude the possibility that branch junctions in the ADP-Pi and ATP states share conformational similarities that would make them hard to distinguish based on the kinetic measurements we have conducted in the present study.

### Molecular implications of the mechanical stability of Arp2/3 complex branches

One very striking observation is the greater stability of Arp2/3 complexes in the ADP-Pi state compared with the ADP state. Both interfaces of the branch junction are more stable when Arp2/3 is in the ADP-Pi state than in the ADP state. Even more striking is the nucleotide-dependent stability of surviving Arp2/3 complexes that remain bound to mother filaments after debranching. This indicates that the presence of Pi in Arp2 and/or Arp3 allosterically modulates the interface of the Arp2/3 complex with the mother filament. This is reminiscent of the role of Pi in F-actin, which was shown to be a central node of the allosteric network that connects the different actin subdomains, in particular subdomain 1 and the D-loop of subdomain 2 (Merino et al., 2018; Oosterheert et al., 2022; Schahl et al., 2025). In addition, surviving ADP-Pi-Arp2/3 complexes do not need to reload ATP to regrow a branch. The presence of Pi seems to maintain Arp2 and Arp3 in an “active” short-pitch conformation, to which actin can bind to elongate a branch. The Pi release is slower in surviving ADP-Pi-Arp2/3 complexes than at branch junctions, which seems to indicate that the presence of the actin subunits of the daughter filament contacting Arp2 and Arp3 adds extra conformational shifts to the nucleotide-binding site. At branch junctions, we observed that Pi easily shuttles in and out of the Arp2/3 complex (Fig. 2). Our *in vitro* observations do not allow us to identify whether Pi binds preferentially to Arp2, Arp3, or both. Previous studies point toward ATP hydrolysis in Arp2 as having a more central role in branch stability (Ingerman et al., 2013; Martin et al., 2006), and taking place sooner than in Arp3 (Dayel and Mullins, 2004; Le Clainche et al., 2003). In our molecular dynamics simulations of the ADP-Arp2/3 complex at a branch junction, the main backdoor through which Pi is thought to be escaping appears to be widely open in Arp2 and closed in Arp3 (Fig. 3).

The interface between the Arp2/3 complex and the mother filament is much less stable for surviving Arp2/3 complexes than for Arp2/3 complexes at branch junctions, by at least 10 k_B_.T in both ADP or ADP-Pi states. This means that the presence of the daughter filament anchors very tightly Arp2/3 complexes to mother filaments, pointing toward an allosteric network connecting the two interfaces of the branch junctions via drastic changes in the Arp2/3 complex conformation.

Structures of ADP- and ADP-BeFx-Arp2/3 branch junctions have been compared by Chavali et al. (2024). They observed minor differences between the two structures. These minor structural differences do not seem to account for the large difference in the stability of branch junctions we report in the present study. The fact that the authors investigated branch junctions using *S. pombe* Arp2/3 complex and mammalian actin may possibly lead to altered interactions between subunits of the Arp2/3 complex and actin subunits of mother and daughter filaments.

The presence or absence of Pi in the Arp2/3 complex strongly controls GMF and cortactin activity on Arp2/3 branch junctions (Figs. 6 and 7). The absence of Pi may render key residues at the surface of the Arp2/3 complex more accessible. Similar to what has been previously proposed for cofilin for binding and severing ADP–actin filaments but not ADP–Pi–actin filaments (Chou and Pollard, 2019; Oosterheert et al., 2022), GMF and cortactin might sense the conformational state of Arp2/3 complex branch junctions imposed by the presence of Pi. Upon binding, cortactin creates additional contacts between the Arp2/3 complex and actin subunits of the daughter filament (Liu et al., 2024). We find that cortactin modifies the energy profile of the Arp2/3 complex–daughter filament bond, by shortening the distance to the energy barrier maximum ($\Delta x_{cortactin}^{daughter}<\Delta x^{daughter}$), without changing the height of the energy barrier $(k_{off,F=0,cortactin}^{daughter}$ ≃ $k_{off,F=0}^{daughter}$). This translates into the interface being less force-sensitive. Nevertheless, similar to the effect of Pi, cortactin also drastically improves the stability of the interface of the ADP-Arp2/3 complex and the mother filament. Altogether, cortactin stabilizes branches and strongly favors branch renucleation (in the 0- to 8-pN force range). In our assays, the continuous presence of cortactin in solution during rebranching prevents us from assessing whether the same cortactin remains bound throughout the debranching–renucleation process. McGuirk et al. recently reported a dwell time of ∼37 s for cortactin at a branch junction using an all-mammalian system, which is quite long (McGuirk et al., 2024). Using fluorescent cortactin, future studies could thus investigate whether cortactin remains attached to the Arp2/3 complex or leaves with the branch.

Regarding GMF, we reveal that it destabilizes the Arp2/3 complex–daughter filament interface, leaving the Arp2/3 complex attached to the mother filament (Figs. 6 and 8). This contradicts interpretations from recent studies, including ours, where it was hypothesized that GMF would induce the departure of the Arp2/3 complex with the branch (Chung et al., 2022; Ghasemi et al., 2024). The subsequent binding of GMF to surviving Arp2/3 complexes appears to dramatically accelerate its dissociation. However, the presence of millimolars of ATP in solution is enough to prevent this outcome.

### Cellular implications of the mechanical stability of Arp2/3 complex branches

In cells, besides elevated ATP (∼ mM) and free monomeric actin (∼ 100 µM) concentrations in the cytoplasm, free Pi could play an important role in regulating actin filament branch stability. We showed that Pi affinity for Arp2/3 complexes at branch junctions is around 3.7 mM (Fig. 1). Pi concentration in the cytosol in different mammalian cell types lies between 1 and 10 mM (Gillies et al., 1982; Soboll et al., 1978), and its concentration is probably locally regulated as it is the case for solutes in general (Kim and Delarue, 2025). In the case of branched actin networks that turn over very fast, such as endocytic patches (turnover rate ∼ 1–2 s [Lacy et al., 2019; Mousavi et al., 2025; Smith et al., 2026]), some Arp2/3 branches may still be in the ADP-Pi state and thus provide some level of mechanical stability to resist to the force produced during membrane invagination.

In actin networks turning over more slowly, the ability of the Arp2/3 complex to switch back toward the ADP-Pi state thanks to Pi in solution could provide an efficient way to regulate GMF debranching activity. Overall, the debranching rate can be modulated by as much as 120-fold, the fastest debranching rate being by GMF acting on the ADP-Arp2/3 complex. GMF could produce short filaments in cells by quickly pruning branches generated by the same Arp2/3 complex, remaining active for several rounds of branch dissociation and regrowth, with GMF being unable to prevent branch renucleation at the millimolar ATP concentration in the cellular environment (Fig. 8 B). Indeed, the presence of short actin “oligomers” has been reported to be key in lamellipodium turnover (Raz-Ben Aroush et al., 2017). In the context of cortex regrowth in blebs, GMF could participate by generating short filaments that invade the bleb to quickly regrow an actin network there (García-Arcos et al., 2024).

Cortactin binding provides a means to delay branch dissociation upon force application, which could be sufficient to maintain branched network integrity under some level of stress in cells. Any significant stress that accelerates debranching would lead to a change in the network architecture, not only by changing the network branch connectivity, but also by generating new filaments through branch renucleation. This mechanically tuned response would be local and would not require membrane-bound NPFs to initiate new branches. Conversely, above a critical mechanical stress level, the Arp2/3 complex detaches with the daughter filament upon debranching, inhibiting renucleation. In cells, the branched actin network would thus rupture in response to high stress, a way to provide a clear response to external mechanical stimuli.

Future studies should explore the role of mechanical stress and Pi on the competition between cortactin and various branch destabilizers, such as ADF/cofilin (Chung et al., 2022), GMF (McGuirk et al., 2024), and coronin (Koundinya et al., 2025; Shatery Nejad et al., 2025). This will allow us to better understand how branched actin networks are regulated in a more complex and physiological context. Moreover, mammalian Arp2/3 complexes exist in different flavors (Cao and Way, 2024), thanks to the various combinations of the isoforms of ArpC1A/ArpC1B, ArpC5/ArpC5L, and Arp3/Arp3B (Abella et al., 2016), and to posttranslational modifications (PTMs). Different flavors of branch junctions can be created, depending on the cell type and the location within the cell (Vasconcellos et al., 2025). Our work sheds light on key factors regulating branch stability and turnover (Pi, regulatory proteins, forces). Future studies should expand this work, and include other factors (Arp2/3 iso-complexes, PTMs) in order to better understand this central player of actin network architecture.

## Materials and methods

### Proteins

Alpha-skeletal actin (UniProt P68135) was purified from rabbit muscle acetone powder following the initial protocol from Spudich and Watt (1971). Briefly, rabbit muscle acetonic powder was obtained from fresh rabbit muscles, which were chopped, then washed by 2 rounds of resuspension in extraction buffer (500 mM KCl, 50 mM KHCO_3_) and centrifugation (4,000 *g*, 10 min, 4°C), then two rounds of resuspension in water whose pH was adjusted with NaCO_3_ to 8.6 and centrifugation (4,000 *g*, 10 min, 4°C). The final pellets were resuspended by manual agitation, frozen, and blended in −20°C acetone. The blended frozen muscles were filtered, and the operation was repeated twice. The resulting muscle paste was spread out and dried overnight at room temperature. The powder was recovered and stored at −20°C until use. Acetonic powder was resuspended in X-buffer (2 mM Tris, pH 7.8, 0.5 mM ATP, 0.1 mM CaCl_2_, 1 mM DTT, 0.01% NaN_3_) and centrifuged (40,000 *g*, 45 min, 4°C). The supernatant was collected and filtered through glass wool. KCl was added to a final concentration of 3.3 M to remove contaminants and the solution centrifuged and filtered as before. The supernatant was then dialyzed overnight against 32 supernatant volumes of dialysis buffer (2 mM Tris, pH 7.8, 1 mM MgCl_2_, 1 mM DTT), which brought the KCl concentration to 0.1 M. KCl was then added to a final concentration of 0.8 M, and the solution was incubated under agitation for 1 h30 at 4°C, then ultracentrifuged at 100,000 *g* for 3 h30 at 4°C. The resulting pellet was resuspended with a potter in X-buffer supplemented with 40 mM KCl and 2 mM MgCl_2_ and incubated overnight at 4°C. KCl was then added to increase its concentration to 0.8 M. The solution was further incubated under agitation for 1 h30 at 4°C, then ultracentrifuged at 100,000 *g* for 3 h30 at 4°C. Repeating those steps as described here allows for a better detachment of undesired actin-binding proteins. The resulting pellet was resuspended using a potter into G-buffer (2 mM Tris, pH 7.8, 0.1 mM CaCl_2_, 0.01% NaN_3_, 0.2 mM ATP, 1 mM DTT), and dialyzed against the same buffer to depolymerize filaments at 4°C for 3 days. The solution was recovered, ultracentrifuged (400,000 *g*, 45 min, 4°C), and injected into an equilibrated Superdex 200 HiLoad column (Cytiva), and actin was eluted with G-buffer into 1-ml fractions. Actin-containing fractions were identified by absorption at 290 nm, validated by electrophoresis, and pooled. The leftmost fractions were excluded as they might contain actin oligomers. The concentration was determined by measuring the optical density at 290 nm. Actin was stored on ice for up to 8 wk, or flash-frozen into liquid nitrogen and stored at −70°C.

Actin was fluorescently labeled on the surface-accessible lysines using Alexa Fluor 488, 568, or 647 NHS ester (Thermo Fisher Scientific) as follows. Actin was dialyzed overnight in modified F-buffer (20 mM PIPES, pH 6.9, 100 mM KCl, 0.2 mM ATP, 0.2 mM CaCl_2_) for polymerization. The resulting filaments were incubated with a 5× excess of fluorophore for 2 h at room temperature on a rotating wheel, then ultracentrifuged at 350,000 *g* for 30 min at room temperature. The resulting pellet was resuspended in G-buffer with a potter, and the actin was left to depolymerize on ice for 2 h. A new round of polymerization was induced by adding 400 mM KCl and 2 mM MgCl_2_ for 1 h at room temperature. The filaments were ultracentrifuged at 350,000 *g* for 30 min at room temperature, and resuspended in G-buffer with a potter. The solution was dialyzed overnight against G-buffer, then ultracentrifuged at 350,000 *g* for 30 min at 4°C. The concentration and labeling fraction were determined by measuring the optical density at 280 and the wavelength of the Alexa dyes. Final labeling fraction was between 15 and 45%, depending on the fluorophore, and used at 10% unless otherwise stated in the main text.

The Arp2/3 complex was purified from the sheep brain thymus, as follows. In a Waring blender, 300 *g* of frozen sheep brain was broken and homogenized into 600 ml of 20 mM PIPES, pH 6.8, 1 mM EGTA, 1 mM DTT, 0.2 mM PMSF, at 4°C. The homogenate was centrifuged at 100,000 *g* for 1 h at 4°C. The supernatant was dialyzed overnight at 4°C against 4 L of 20 mM PIPES, pH 6.8, 1 mM EGTA, 1 mM MgCl_2_, 1 mM DTT, using a 30,000-kDa MWCO dialysis membrane. The supernatant was centrifuged at 100,000 *g* for 30 min at 4°C. The supernatant was loaded onto a SP-Trisacryl column equilibrated with the dialysis buffer. The resin was washed with 5 volumes of the dialysis buffer, then washed a second time with 150 ml of the same buffer supplemented with 30 mM NaCl. The resin was eluted with 150 ml of the same buffer supplemented with 100 mM KCl. The peak fractions were dialyzed against 500 ml of 20 mM Tris-HCl, pH 7.4, 25 mM KCl, 0.5 mM EDTA, 1 mM MgCl_2_, 0.25 mM DTT, 100 µM ATP, using a 30,000-kDa MWCO dialysis membrane overnight at 4°C. The proteins were loaded on a glutathione S-transferase (GST)-VCA–bound Sepharose-GSH column, previously equilibrated in the aforementioned buffer. The proteins were washed first with the same buffer, and a second time with the same buffer supplemented with 200 mM KCl. The proteins were eluted with the same buffer this time supplemented with 200 mM MgCl_2_. The peak fractions were pooled and dialyzed overnight at 4°C against the same buffer (not supplemented with KCl or MgCl_2_). Sucrose was added to a final concentration of 200 mM using a 2 M sucrose stock solution. The Arp2/3 complex concentration was measured, flash-frozen in liquid nitrogen, and stored at −70°C. The Arp2/3 complex can be stored for months at −70°C. Thawed aliquots kept on ice can be used for 2 days.

The fluorescently labeled Arp2/3 complex used in Fig. 5 was prepared using recombinantly expressed human Arp2/3 complexes, produced, and purified as briefly described here. First, to generate baculoviruses, bacmid DNA (2 μg) was mixed with 100 μL SF900-III medium (Life Technologies) and 3 μl FuGENE HD transfection reagent, and incubated for 15 min before being added dropwise to 10^6^ adherent Sf21 insect cells with 2 ml of SF900-III medium at 27°C. After 3 days, the supernatant (P1 virus) was added to a 50 ml culture of Sf21 cells (1–2 × 10^6^ cells/ml) in SF900-III medium with constant shaking at 110 rpm at 27°C. After 3 days, 50 μl of supernatant (P2 virus) was used to infect a second 50 ml Sf21 culture and incubated for 3 days. The resulting supernatant (P3 virus) was stored at 4°C, and 500 μl was used to infect 0.5 L of Sf21 insect cells at 1–2 × 10^6^ cells/ml for protein production. Three days after infection, cell pellets were harvested by centrifugation, washed with phosphate-buffered saline (PBS), flash-frozen in liquid nitrogen, and stored at −80°C. Frozen cell pellets were resuspended to a final volume of 50 ml in purification buffer (50 mM Tris, pH 8, 150 mM NaCl, 2 mM MgCl_2_, 2.5% vol/vol glycerol, 1 mM DTT, 0.2 mM Mg-ATP) supplemented with cOmplete EDTA-free Protease Inhibitor Cocktail (Roche) and BaseMuncher Endonuclease (5 μl per 50 ml) (Cat. # ab270049; Abcam). Resuspended cells were lysed by sonication. After 1-hour incubation, EDTA and EGTA were added to a final concentration of 1 and 5 mM, respectively. The lysate was then clarified by ultracentrifugation at 190,000 *g* for 45 min at 4°C, followed by passage through a 0.45-μm syringe filter. The resulting filtrate was loaded onto a 1 ml StrepTrap XT column (Cytiva). After washing with the purification buffer, the bound Arp2/3 complex was eluted with buffer BXT (IBA) supplemented with 2 mM ATP, 1 mM DTT, 5 mM EGTA, 2 mM MgCl2, and 2.5% vol/vol glycerol. Fractions containing Arp2/3 were pooled and loaded on a Superdex 200 Increase 10/300 Gl column (Cytiva) pre-equilibrated with the purification buffer containing 5% vol/vol glycerol. Fractions (0.4 ml) were analyzed by SDS-PAGE. Fractions containing Arp2/3 were pooled and concentrated to ∼4–5 mg/ml. Small aliquots were flash-frozen in liquid nitrogen and stored at −80°C.

The recombinant Arp2/3 complex was then fluorescently labeled using Alexa Fluor 488 C5-maleimide (Thermo Fisher Scientific). First, DTT was removed from the protein solution by buffer exchange using a Micro Bio-Spin 6 column (Bio-Rad) preloaded with 20 mM Hepes, pH 7.2, 0.2 mM MgCl_2_, 0.2 mM ATP. Next, 10-fold molar excess of Alexa Fluor 488 C5-maleimide in DMSO was added to the Arp2/3 complex solution and incubated on ice for 1 h. The reaction was quenched by adding 1 mM DTT. Unreacted dye was removed by buffer exchange using a Micro Bio-Spin 6. On average, 3.5 Alexa dyes were covalently bound per the Arp2/3 complex.

Spectrin–actin seeds were purified from human erythrocytes as follows. Human erythrocytes were first washed by repeated cycles of centrifugation (3,000 *g*, 15 min, 4°C) and pellet resuspension in PBS buffer with EDTA (5 mM NaPO_4_, pH 7.7, 150 mM NaCl, 1 mM EDTA). Erythrocytes were then lysed in a low ionic strength buffer (5 mM NaPO_4_, pH 7.7, 1 mM PMSF) to turn them into cell ghosts. Cell ghosts were then washed and concentrated by 3 cycles of centrifugation (45,000 *g*, 15 min, 4°C) and pellet resuspension in washing buffer (5 mM NaPO_4_, pH 7.7, 0.1 mM PMSF). The final pellet was resuspended and incubated at 37°C for 40 min with occasional mixing. Cells were then ultracentrifuged at 400,000 *g* for 1 h at 4°C, to remove membrane residues. The supernatant was complemented with 2 mM DTT and protease inhibitors, and an equal volume of ice-cold glycerol was added. The concentration of functional spectrin–actin seeds was determined using a pyrene actin assay, to measure the concentration of growing filament barbed ends, using a spectrofluorometer (Xenius instrument from SAFAS [Monaco]; 10% labeled pyrene actin, excitation 366 nm, emission 407 nm; at room temperature). Spectrin–actin seed solution was then aliquoted and stored at −20°C until used (stable for months).

Recombinant N-terminal GST-tag human N-WASP-VCA (amino acids 392–505, UniProt O00401) and mouse cortactin full length (amino acids 1–546, UniProt Q60598) were expressed and purified as follows. Expression was performed in *Escherichia coli* Rosetta 2 (DE3) for 16 h at 18°C. Proteins were purified by affinity chromatography over a Sepharose 4B GSH affinity column (Cytiva) in GST buffer (20 mM Tris, pH 7.5, 500 mM NaCl, and 1 mM DTT). The protein was eluted with the same buffer supplemented with 50 mM GST. The purification is followed by gel filtration over a Superdex 200 HiLoad column (Cytiva) in GF-buffer (20 mM Tris, pH 7.5, 50 mM KCl, 1 mM DTT, and 5% glycerol). Before the gel filtration step, the GST-tag of GFP-N-WASP-VCA and cortactin was cut by PreScission Protease (Sigma-Aldrich) overnight at 4°C to obtain monomeric N-WASP-VCA and untagged full-length cortactin.

Recombinant N-terminal 6xHis-tagged human GMFγ (amino acids 7–140, UniProt O60234) was a gift from Nicola Burgess-Brown, Center for Medicines Discovery, Univ. of Oxford, Oxford, UK (plasmid # 39037; Addgene; http://n2t.net/addgene:39037; RRID:Addgene_39037), and was expressed in *E. coli* Rosetta 2 (DE3) for 16 h at 18°C. Proteins were purified by affinity chromatography over a HisTrap HP column (Cytiva) in His-buffer (20 mM Tris, pH 7.5, 500 mM NaCl, 1 mM DTT, and 20 mM imidazole) and eluted in the same buffer supplemented with 300 mM imidazole. The purification was polished by gel filtration over a Superdex 200 HiLoad column (Cytiva) in 20 mM Tris, pH 7.5, 500 mM NaCl, and 1 mM DTT, and stored at −70°C.

Recombinant human profilin1 (UniProt P07737) was expressed in *E. coli* (BL21 DE3 Star, Thermo Fisher Scientific) at 37°C for 2.5 h. After centrifugation at 2,000 *g*, the pellet was resuspended in lysis buffer (Tris 50 mM, pH 7.3, 5 mM EGTA, 0.1 mM EDTA, 50 mM KCl, 10 mM DTT, 8 M urea, 0.1% Tween-20, 1 mM PMSF, inhibitors). After sonication, the lysate was centrifuged at 186,000 *g*, 30 min, 4°C. The supernatant was dialyzed overnight at 4°C against the dialysis buffer (Tris 50 mM, pH 7.3, 1 mM EGTA, 0.1 mM EDTA, 50 mM KCl, 1 mM DTT). The protein solution was loaded onto a poly-L-proline column, washed with 3 column volumes of washing buffer (mixture of 3 volumes of dialysis buffer + 1 volume of elution buffer), and eluted with elution buffer (Tris 50 mM, pH 7.3, 5 mM EGTA, 0.1 mM EDTA, 50 mM KCl, 10 mM DTT, 8 M urea). Peak fractions were pooled, dialyzed overnight at 4C against dialysis buffer, concentrated with Vivaspin (cutoff 10 kDa), and dialyzed overnight at 4°C against conservation buffer (Tris 10 mM, pH 7.5, 50 mM KCl, 1 mM DTT). The concentration was determined by absorption at 280 nm using the calculated extinction coefficient (17,020 M^−1^.cm^−1^). The protein solution was aliquoted, flash-frozen, and stored at −70°C.

### Buffers

Standard microfluidics experiments were performed in standard F-buffer containing 5 mM Tris-HCl at pH 7.0, 1 mM MgCl_2_, 0.2 mM EGTA, 0.2 mM ATP, 10 mM DTT, 1 mM DABCO, supplemented with 0.1% bovine serum albumin (BSA) and 50 mM KCl. For Pi experiments, F-buffer was supplemented with KH_2_PO_4_/K_2_HPO_4_ to reach the desired phosphate concentration at pH 7.0, and the concentration of KCl was adjusted to reach a final ionic concentration of 81 mM. BeFx solutions were prepared by mixing 2 mM BeSO_4_ and 10 mM NaF in an F-buffer containing 50 mM KCl.

Open chamber experiments to investigate debranching rate without any pulling force were performed using F-buffer with 50 mM KCl, supplemented with 0.5% methylcellulose 4,000 cP.

### Data acquisition

Experiments were performed using a Nikon TiE inverted microscope equipped with a total internal reflection fluorescence (TIRF) CFI Apo 60× 1.49 NA oil-immersion objective, a Kinetix22 sCMOS camera (Photometrics), and a TIRF illumination setup (iLAS2, Gataca Systems) with 100-mW 488-, 561-, and 642-nm tunable lasers. The temperature was maintained in the chamber assay at 25 (±0.2)°C using a collar objective heater (Okolab). The setup was controlled using MicroManager (Edelstein et al., 2014).

### Microfluidics experiments

Microfluidics experiments were conducted in polydimethylsiloxane (Sylgard) chambers based on the original protocol from Jégou et al. (2011), described in detail in Wioland et al. (2022). First, spectrin–actin seeds were attached to the glass surface by flowing in a solution containing 5 pM in F-buffer, for 4 min. Next, the surface was passivated by exposing it to a solution containing 5% BSA for at least 20 min. Surface-anchored mother filaments were polymerized by flowing in 0.6 μM 10% Alexa Fluor 488–labeled G-actin for 5–10 min. Next, the nucleation of actin filament branches was triggered by flowing in a solution of 20 nM Arp2/3 complex, 50 nM VCA, and 0.4 μM 10% Alexa Fluor 568–labeled G-actin, for around 75 s to obtain a branch density ∼ 1 branch every 10 µm along mother filaments. For experiments in Fig. 1, actin filament branches and mother filaments were aged using a solution at low flow rate containing 0.2 μM 10% Alexa Fluor 568–labeled G-actin for 30 min (or 4 min for Fig. S1, A and B). Finally, actin filaments were exposed to debranching conditions. For constant force pulling experiments, we used a solution containing 0.1 μM 10% Alexa Fluor 568–labeled G-actin in different buffer conditions depending on the assay, in the presence or absence of GMF or cortactin, with varying acquisition frame rates, ranging from 1 image every 0.5 s to 10 s. Please refer to the legend of each corresponding figure for a description of the buffer and protein compositions.

For cortactin experiments, we chose to use 20 nM as our standard concentration for cortactin to be at saturating activity, based on the published results from McGuirk and colleagues, who reported an apparent binding affinity of mammalian cortactin for mammalian Arp2/3 complex at branch junction of 1.3 nM (McGuirk et al., 2024).

### Open chamber experiments

To measure branch detachment without applying any pulling force, we prepared open chambers by melting parafilm stripes sandwiched in between cleaned coverslips. This creates chambers of around 10 μl. Chambers are first passivated with 5% BSA solution for 10 min, then extensively rinsed with F-buffer with 50 mM KCl. Mother filaments are first assembled in a tube for 10 min at room temperature, using 5 µM 10% Alexa Fluor 488–labeled G-actin in F-buffer, with 50 mM KCl. Second, 1.25 µM of preformed mother filaments is next injected inside the chamber in a buffer containing 100 nM Arp2/3, 200 nM VCA, 0.4 µM 10% Alexa Fluor 568–labeled G-actin, 0.4 µM profilin, supplemented with 0.25% methylcellulose. We let the branching reaction proceed for 1 min. The branching solution is then replaced by flowing in a solution containing 0.04 µM 10% Alexa Fluor 568–labeled G-actin F-buffer, 50 mM KCl, and 0.5% methylcellulose. Images are acquired at a frame rate of 1 frame every 30 s in TIRF for a total duration of 60 min.

### Data analysis

#### Fraction of surviving branches

The fraction of surviving branches as a function of time is computed by the Kaplan–Meier method using the “lifelines” package from Python. The error bars represent the 95% confidence interval calculated with the Greenwood’s exponential formula.

#### Branch renucleation ratio

The branch renucleation ratio represents the ratio of the number of renucleated branches to the number of dissociated branches. Error bars show the binomial standard deviation.

#### Force applied to the branch junction

For microfluidics experiments, the tensile force exerted on branches is a result of the viscous drag applied by the fluid to the filaments, as characterized previously in Jégou et al. (2013) for actin filaments. The applied pulling force at the branch junction is F = v_flow_ × L_branch_ × η_actin_, where L_branch_ is the length of the daughter filament, v_flow_ the local flow velocity at 250 nm above the glass surface, and η_actin_ the longitudinal friction coefficient per unit length of the actin filament (η_actin_ = 6 × 10^−4^ pN.μm^−2^.s), determined in a previous publication (Jégou et al., 2013). In each experiment, the reported average force and standard deviation of the force were determined by computing the force applied on each individual analyzed branch at the time of branch dissociation.

#### Affinity constant between Pi and ADP-Arp2/3 complex at branch junctions

The affinity constant of Pi for the ADP-Arp2/3 complex at a branch junction (Fig. 2) was derived fitting the observed debranching rates measured at 3.3 pN as a function of phosphate concentration, using the following equation: k_deb._ ([Pi]) = k_max_ + (k_min_-k_max_).([Pi]/(K_D_+[Pi])), with k_min_, k_max_, and K_D_ as free parameters. The numerical fit was performed using the “curve_fit” function from the SciPy Python package.

#### Computation of the Pi release rate from Arp2/3 complex branch junctions in buffer switching experiments

Based on the Pi affinity constant, initially 93% of Arp2/3 complexes at branch junctions are in the ADP-Pi state in the presence of a 50 mM Pi buffer. Upon switching to standard F-buffer, either a branch detaches with its Arp2/3 complex in the ADP-Pi state, with a rate k_deb, ADP-Pi_, or the Arp2/3 complex releases its Pi, with a rate k_Pi rel._, and then, the branch detaches with the Arp2/3 complex in the ADP state, with a rate k_deb, ADP_. Our assay only monitors the overall debranching rate from those of the two evolving ADP- and ADP-Pi-Arp2/3 complex populations. The population of branch junctions with Arp2/3 complexes in the ADP-Pi state decays following the equation N_ADP-Pi_(t) = N_0_.exp[-(k_deb,ADP-Pi_ + k_Pi rel._).t], where N_0_ is the initial size of the population of branches at t = 0 s. The population of branches with Arp2/3 complexes in the ADP state evolves following the equation: ${\text{dN}}_{\text{ADP}}\left(t\right)/\text{dt}=k_{\text{Pi}\;\text{rel}.}\;.\;N_{\text{ADP}-\text{Pi}}\left(t\right)-k_{\text{deb},\text{ADP}}.\;N_{\text{ADP}}\left(t\right)$. Solving analytically the differential equation, we obtain that the overall population of branches decays following the equation:

$$\begin{array}{c} N_{ADP-Pi}\left(t\right)+N_{ADP}\left(t\right)=\\ \frac{N_{0}}{k_{deb.,ADP}-k_{deb,ADP-Pi}-k_{Pi\;rel.}}\\ \left[\left(k_{deb.,ADP}-k_{deb,ADP-Pi}\right)e^{-\left(k_{deb.,ADP-Pi}+k_{Pi\;rel.}\right)t}-k_{Pi\;rel.}e^{-k_{deb,ADP}t}\right] \end{array}$$

This equation was used to fit the experimental data, with all 3 rates as free parameters for the fit.

#### Distribution of the nucleotide states of surviving Arp2/3 complexes upon debranching in the presence of 50 mM Pi in solution

In the presence of 50 mM phosphate in solution, Arp2/3 complexes at branch junctions are in rapid equilibrium between ADP and ADP-Pi states. The equilibrium ratio between the two states is set by the affinity constant (3.7 mM, Fig. 2 C): 93% in ADP-Pi, 7% in ADP. According to the Curtin–Hammett principle, upon debranching, being considered to be an irreversible reaction, the product ratio of debranching events between Arp2/3 complexes in the ADP-Pi and ADP states can be expressed as a function of the equilibrium ratio between the two states at branch junctions ([Pi]/K_D_) and the ratio between the debranching rates in the two states, k_deb ADP_/k_deb, ADP-Pi_. In other words, the ratio of surviving Arp2/3 complexes between ADP-Pi and ADP states can be written as follows:

$$\frac{surv.\;ADP-Pi}{surv.\;ADP}=\frac{k_{deb,ADP-Pi}\left[Pi\right]/K_{D}}{k_{deb.,ADP}}.$$

Upon detachment, the proportion of surviving ADP-Pi-Arp2/3 complexes is thus $\frac{k_{deb,ADP-Pi}\left[Pi\right]}{k_{deb,ADP-Pi}\left[Pi\right]+k_{deb.,ADP}.K_{D}}≃32\%$.

For this formalism to be considered a good approximation, the debranching rates should be at least 10 times slower than the Pi exchange rates, which is the case in the presence of 50 mM phosphate and at all forces we used in our microfluidics experiments.

#### Dissociation rate of the surviving Arp2/3 complex from mother filaments

The dissociation rates of surviving Arp2/3 complexes in the ADP and ADP-BeFx states (Fig. 5 C) were derived from the exponential fits of the surviving fractions of fluorescently labeled Arp2/3 complexes, in the absence of actin in solution, acquired at a frame rate of 1 image per second for ADP-Arp2/3 complexes, and 1 image every 2 s for ADP-BeFx-Arp2/3 complexes.

In the presence of 50 mM Pi in solution, the surviving Arp2/3 complexes are 68% in the ADP state, and 32% in the ADP-Pi state (see above). We consider that surviving ADP-Arp2/3 complexes quickly reload ATP and dissociate as ATP-Arp2/3 complexes with the rate measured in regular buffer, k_off_^ATP-Arp2/3^. Surviving ADP-Pi-Arp2/3 complexes can either release their Pi with the rate k_Pi rel._, reload ATP and dissociate as ATP-Arp2/3 complexes with the rate measured in regular buffer (k_off_^ATP-Arp2/3^), or dissociate from the mother filament in the ADP-Pi state, with the rate we estimated using BeFx, k_off_^ADP-BeFx-Arp2/3^. The fraction of surviving Arp2/3 complexes in the presence of 50 mM Pi was thus fitted using the following equation:

$$\begin{array}{c} S_{50mM\;Pi}\left(t\right)=0.68.e^{-k_{off}^{ATP-Arp2/3}.t}+\frac{0.32}{k_{off}^{ATP-Arp2/3}-k_{off}^{ADP-BeFx-Arp2/3}-k_{Pi\;rel.}}\\ \left[\left(k_{off}^{ATP-Arp2/3}-k_{off}^{ADP-BeFx-Arp2/3}\right)e^{-\left(k_{off}^{ADP-BeFx-Arp2/3}+k_{Pi\;rel.}\right).t}-k_{Pi\;rel.}e^{-k_{off}^{ATP-Arp2/3}.t}\right] \end{array}$$

with k_Pi rel._ as the only free parameter.

Additionally, as shown in Fig. S6, the slow dissociation rate of surviving ADP-BeFx-Arp2/3 complexes was further validated using unlabeled Arp2/3 complexes. For this, we assessed the renucleation ratio of unlabeled ADP-BeFx-Arp2/3 complexes on mother filaments over time: first, branches were detached in a BeFx buffer without actin for several minutes, before re-exposing to 0.5 µM actin. Arp2/3 complexes were grouped based on the time at which they lost their branches, and the renucleation ratio of each group was evaluated when exposing to actin.

All fits were done using the least-square minimization procedure of the curve_fit function from the SciPy Python package.

#### Molecular dynamics simulations

Simulations were initiated by using the Cryo-EM structure of the ADP-Arp2/3 complex at branched actin junction (PDB 7TPT) (Ding et al., 2022). In this structure, the Arp2/3 complex is in direct contact with 6 actin subunits of the mother filament and 2 actin subunits of the daughter filament. Thus, to reduce the computational cost of the simulations, we only kept these actin subunits for the upcoming steps of the modeling. Initial systems were built using CHARMM-GUI (Jo et al., 2008) to solvate the full complex in a box of explicit water molecules and neutralized in 150 mM KCl. The water box was set rectangular with at least 2-nm distance from the edge to the solute.

Parameters of the systems were as follows: 232 k water molecules; 774 k atoms; box dimensions 14 × 23 × 23 nm^3^; duration 1 µs.

Molecular dynamics simulations were performed using GROMACS 2023.2 (Abraham et al., 2015). The CHARMM36m (Huang and MacKerell, 2013) force field was employed in combination with the TIP3P (Jorgensen et al., 1983) water model. The systems underwent energy minimization using the steepest descent algorithm until the energy gradient converged to a threshold of 0.01 kcal/mol/Å. The solvent was then allowed to relax in the NVT ensemble at 300 K for 625 ps, while the backbone and sidechain atoms of all proteins were restrained using harmonic force constants of 2,000 kJ.mol^−1^.nm^−2^ and 1,000 kJ.mol^−1^.nm^−2^, respectively. Each system then underwent NPT equilibration for 3 ns, first increasing the timestep from 1 fs to 2 fs, then progressively removing all restraints on the Arp2/3 complex and the daughter filament. For the backbone atoms of actin subunits forming the mother filament, we kept restraints using harmonic force constants of 100 kJ.mol^−1^.nm^−2^ during equilibration and production. It allowed us to (1) avoid potential interactions through periodic boundary conditions, and (2) mimic the interactions of the Arp2/3 complex with a stable mother filament. The LINCS algorithm (Darden et al., 1993) was used for bond constraints.

During equilibration, the Berendsen thermostat (Berendsen et al., 1984) and barostat (when applicable) were used. For production, the v-rescale thermostat (Bussi et al., 2007) and c-rescale barostat (Bernetti and Bussi, 2020) were used for temperature and pressure control, respectively. For equilibration and production runs, a cutoff distance for nonbonded interactions was set at 1.2 nm, utilizing a cutoff van der Waals type with a force-switch modifier. The switching distance was configured to 1.0 nm. Coulombic interactions were calculated using the particle mesh Ewald method (Darden et al., 1993), with a cutoff distance of 1.2 nm. The analysis of the distances between residues was conducted on the last 300 ns of the 3 repeats, as previously published in Schahl et al. (2025).

### Online Supplemental material

Fig. S1 shows impact of Arp2/3 complex aging and force on the debranching rate. Fig. S2 shows the nucleotide state of the mother filament does not affect the debranching rate. Fig. S3 shows Pi release rate from within Arp2/3 complex at branch junctions. Fig. S4 shows simultaneous fit of debranching rate and branch renucleation ratio. Fig. S5 shows ADP-BeFx-Arp2/3 complex renucleates for several rounds with a high renucleation ratio. Fig. S6 shows surviving ADP-BeFx-Arp2/3 complexes detach slowly from mother filaments. Table S1 shows parameters of the stability of the Arp2/3 complex interfaces.

## Supplementary Material

Review History

Table S1 shows parameters of the stability of the Arp2/3 complex interfaces.

## Data Availability

The data are available from the corresponding author upon reasonable request.
