# Supplementary material for: Inorganic phosphate rapidly switches the stability of Arp2/3-induced actin branches
Source: J Cell Biol. 2026 Jul 9;225(9):e202605018. doi: 10.1083/jcb.202605018 (PMC13348673; doi:10.1083/jcb.202605018)
Supplement: Table S1 — shows parameters of the stability of the Arp2/3 complex interfaces. [file jcb_202605018_tables1.docx]

### **Table 1 - Parameters of the stability of the Arp2/3 complex interfaces**

Obtained from the simultaneous fit of the debranching rate and the branch renucleation ratio as a function of force.

|  | Arp2/3-daughter filament interface | | Arp2/3-mother filament interface | |
| --- | --- | --- | --- | --- |
| branch junction | $k_{off, F=0}^{daughter}$ (s^-1^) | ${\Delta x}^{daughter}$ (nm) | $k_{off, F=0}^{mother}$ (s^-1^) | ${\Delta x}^{mother}$ (nm) |
| **ADP-Arp2/3 complex** | 3.9 (±0.05) 10^-4^ | 4.5 (±0.15) | 1.6 (±0.7) 10^-5^ | 6.5 (±0.15) |
| **ADP-Pi-Arp2/3 complex** | 8.0 (±0.1) 10^-6^ | 5.1 (±0.1) | 6.0 (±5.5) 10^-8^ | 6.7 (±0.25) |
| **cortactin-bound ADP-Arp2/3 complex** | 3.8 10^-4^ | 2.8 | 3.7 10^-7^ | 6.0 |
