# Supplementary material for: Inorganic phosphate rapidly switches the stability of Arp2/3-induced actin branches
Source: J Cell Biol. 2026 Jul 9;225(9):e202605018. doi: 10.1083/jcb.202605018 (PMC13348673; doi:10.1083/jcb.202605018)
Supplement: Review History [file jcb_202605018_review_history.pdf]

Inorganic phosphate rapidly switches the stability of Arp2/3 induced actin branches

Jiu Xiao, Foad Ghasemi, Adrien Schahl, Miroslav Mladenov, Rebecca Pagès, Mattheu Chavent, Michael Way, Luyan Cao, Guillaume Romet-Lemonne, and Antoine Jégou

Corresponding Author(s): Antoine Jégou, Institut Jacques Monod

Review Timeline:		
	Submission Date:	2026-05-06
	Editorial Decision:	2026-05-29
	Revision Received:	2026-06-08

Monitoring Editor: Matthew Welch

Scientific Editor: Gabriele Stephan

Transaction Report:

(Note: With the exception of the correction of typographical or spelling errors that could be a source of ambiguity, letters and reports are not edited. The original formatting of letters and referee reports may not be reflected in this compilation.)

DOI: https://doi.org/10.1083/jcb.202605018

Revision 0

Review #1

1. Evidence, reproducibility and clarity:

Evidence, reproducibility and clarity (Required)

Summary

This study investigates the mechanochemistry of Arp2/3-mediated branched actin networks at the level of individual branch junctions under load. Using microfluidic single-filament/branch force assays (including constant-force flow and open-chamber imaging) the authors quantify debranching, re-nucleation, and mother- vs daughter-interface stability across nucleotide states of Arp2/3 (ADP-Pi, ADP, and an ADP-BeFx proxy for ADP-Pi). They further test effects by two branch regulators (GMF and cortactin). Key findings include: (i) ADP-Pi and ADP complexes share similar force dependence but differ markedly (~20×) in intrinsic dissociation rate; (ii) phosphate turnover on the Arp2/3 complex is rapid ii) affinity for Pi drops when Arp2/3 loses its daughter filament; (iii) quantification from model fits uncovers large stability differences between daughter and mother interfaces of the Arp2/3 complex; (iv) extraordinary high stability of ADP-Pi-like Arp2/3 on the mother filament; and (v) distinct effects of GMF and cortactin on force-dependent stability. Overall, the work combines technically demanding measurements with mechanistic modeling to probe how nucleotide state and regulatory factors tune branch mechanics.

Major comments:

1. Low force kinetics and completeness of survival curves (Figure 1). "For all forces, the surviving curves exhibited a clear single exponential behavior...." While the data can be fitted to monoexponential decay curves, data at low forces is clearly incomplete. >90% of branches have not dissociated by the end of the experiment. For the particular data shown in 1C (F00nN, n=60 total branches) it means that the time information is coming from <6 observations, which is rather low for the single molecule field. I am slightly worried by this point, since the debranching rates under ADP-Pi conditions at zero force, are even by one magnitude slower. Yet, no raw data is shown. Given that the dissociation rate at low forces is a contentious point, the authors should show the raw data and the corresponding fits. At present, they only show an experimental scheme and images for these "open chamber" assay (Fig S2). Ideally, they would image for much longer than 900s with lower sampling time in those assays, to firmly establish that 20-fold difference also holds at 0 force.

Essential; experiment might already be performed. Otherwise straightforward to do (weeks time).

2. Stability Analysis (Figure 4). I can follow much of the arguments presented in the stability analysis of the daughter vs mother interfaces, which is in principle extremely interesting! However, there are some concerns here:

i) The authors emphasize the zero force ratio derived from fits (which is linked to the stability difference of the two interfaces in the absence of force) despite this being only weakly constrained by data. Intuitively in the model, the stability difference should grow to very large values as the re-nucleation ratio approaches 1 at low force. This combined with the noise in the data poses an issue in my opinion. Looking at the data and the error margin, I think that the authors cannot state with high confidence that there is a real difference between the relative stability of the daughter and mother interfaces between the two nucleotide states of the complex.

Essential; analysis and textual revision only

ii) For ADP-Pi, the renucleation ratio essentially remains flat over the measured force range. Hence, the data can only provide little leverage to estimate both the zero force ratio and, more importantly, the differential distance to the transition state in the slip-bond model in my opinion, which will show in the crossover force. Consequently, the quoted ">100×" stability difference at F=0 and the crossover force >20pN are driven largely by extrapolation rather than direct constraint by data. Given the high number of free parameters in the model, I would anticipate that several crossover forces and differential distances might explain the data nearly equally well. Instead of loosely reporting exact number from fits, I would have hoped for some sort of sensitivity analysis, for instance relying on profile likelihoods. Also parameter values could be reported as bounds (e.g crossover force≫measured range) rather than precise point estimates. This issue re-occurs (albeit not as drastically) for the cortactin experiments (Figure 6).

Essential; analysis and textual revision only

iii) One important expectation from the "two slip bond" model is that branch dissociation rates should not necessarily scale mono-exponentially as they mostly do over the accessible force range of the paper. However, once the "minor" pathway of dissociation

from the mother starts to dominate at high forces, rates become more force sensitive. This is nicely recaptured by the model fits in Figure S6 but deserves some explanation in the text. Otherwise, people will simply remember the "ADP-Pi is 20-fold more stable than ADP at all forces" message.

Essential; textual revision only

iv) One important prerequisite for the model is that isolated Arp2/3 complexes (without a daughter filament) should dissociate with equal rates from mother filaments at all flow rates. Since the Arp2/3 complex prefers mother filament curvature, forces experienced by the mother might change its off-rate. It would be good to refer to this assumption in the text and experimentally verify it. I could not find it in the paper nor in Ghasemi et al 2024.

Essential; simple experiment (a weeks time).

v) The force dependence of the branch re-nucleation rate (Fig 3D) has been measured previously by the same group (Ghasemi et al). While the data in the older paper has not been fitted by a model, the trend of the data in the previous paper looks conspicuously different. Are there any explanations for this? I speculate that it might be related to actin and ATP not being saturated (low-force re-nucleation rate rarely exceeds 80%) in Ghasemi et al., but it would be good to know what the authors think about this.

Essential; textual revision only

3. Stability of the authentic ADP-Pi-Arp2/3 complex on the mother filament. The extraordinary stability of the isolated ADP-BeFx-Arp2/3 complex on mother filaments is surprising, especially considering that both ATP and ADP states are much more labile (Ghasemi et al 2024). I would recommend repeating this experiment in the authentic ADP-Pi state with labelled Arp2/3 complexes as a more direct readout, even if this would require working with very high phosphate concentrations.

Essential; simple experiment (a weeks time).

OPTIONAL: Further, but beyond the scope of the present paper, would be titrating phosphate in these experiments, which would even allow the authors to independently verify the reduced Pi affinity for Arp2/3 in the mother filament. Of note, this affinity difference is needed to satisfy detailed balance in the reaction scheme (Fig 4 D)!

4. Importance of "surviving" ADP-Pi-Arp2/3 complexes. The authors show a) rapid turnover of Pi on the ADP-Arp2/3 complex in both branch- or mother filament-bound state and b) the lowered Pi affinity of the latter. Nonetheless, they emphasize the importance of long-lived "surviving" ADP-Pi bound complexes on the mother (even stated in the abstract). I understand that this fraction shows under some experimental conditions (BeFx), but unless I am missing something, most complexes should rapidly lose their phosphate and either exchange nucleotide or dissociate from the mother under physiological conditions. Please clarify or tone done.

Essential; textual revision only
5. GMF mechanism. The authors claim that GMF "...accelerates the departure of the surviving Arp2/3 complex from the mother...". I assume that they infer this from decrease in the re-nucleation ratio. However, alternatively GMF could simply dwell on the complex, inhibiting re-nucleation without promoting dissociation from the mother. The authors should either monitor Arp2/3 dwell times directly to discriminate between these possibilities or be more cautious in their conclusions.

Essential; simple experiment (a weeks time) or textual revision.
6. Cortactin mechanism and the "leash model". I must say that the cortactin data are the most puzzling part of the paper and had to reconcile with what we know from structure. I was hoping to find some of this resolved in the discussion. However, I do not understand the "leash model" in the discussion section for cortactin-mediated branch stabilization: "This would explain the observed increase in branch survival compared to the absence of cortactin. As the pulling force is increased, this rebinding mechanism becomes less efficient." According to my understanding of the data, this is opposite to what happens. Cortactin only stabilizes the labile interface at elevated forces! Some re-writing might help here.

Essential; textual revision.

Minor comments

Organization:

- I do not want to impose on how to best tell the story, but I felt that Fig1 A-D and Fig 2 A-B belong to one logical unit (nucleotide dependence), whereas Fig 1 E-F and Fig 2 C belong to

the other (Pi binding and exchange). Perhaps consider re-organizing to streamline presentation?

Semantics/Typos:

- Abstract: „... ADP-Pi and ADP-Arp2/3 detach with the same exponential increase as a function of force...". Increase should refer to the dissociation rate, which should be added to the sentence.
- Results page 8: "...and the majority of Arp2/3 complexes detach from the mother filament while remaining bound to the branch at the debranching time." "Branch" should likely be daughter here, as there is no branch after dissociation of either interface.
- Results page 13: "Exposing ADP-BeFx-Arp2/3 complex branch junctions to a saturating amount of GMF...". It is strange to imply saturation, because GMF likely simply does not bind to the complex in this nucleotide state with appreciable affinity. Suggest to change to "high".
- Discussion page 18: "Moreover, in mammalian Arp2/3, His80 in Arp3 (corresponding to His73 in mammalian actin) is not methylated, and corresponds to residue N77 in Arp3, which is also not modified." N77 likely belongs to Arp2?
- Discussion page 19: "We showed that Pi affinity for Arp2/3 complexes at branch junctions is around 3.7 mM (Fig. 1), a value which lies within the reported 1-10 mM Pi concentration measured in the cytosol in different mammalian cell types". Notably, this is not too different from F-actin, which should be mentioned. By this measure alone, free inorganic phosphate could also directly regulate actin filament stability!

Future interest (non essential):

- It would be utterly exciting (but beyond current scope) to quantify how instantaneous debranching rates evolve for naturally aging branches starting from ATP-Arp2/3 complexes!

2. Significance:

Significance (Required)

General assessment:

This is a compelling and carefully executed study that delivers a clear mechanistic framework for how Arp2/3 branch junctions fail and re-form under load. The central strength is the tight integration of state-of-the-art reconstitutions with careful and original

kinetic analysis. The experimental design is elegant and experiments have been carried out to a masterful standard. The figures are clear, the statistics are appropriate with some exceptions as detailed above. There are very few labs in the world that could have achieved this feat!

A few aspects could be further strengthened, most notably the explanation and application of the "two slip bond" model as well as slightly more restraint in speculating around specific regulatory mechanisms. However, these are minor refinements that do not detract from the important contributions of the paper.

Overall, the clearly work merits publication with high priority after revision; most requested changes are textual/analytical with very few targeted experiments, which would substantially strengthen core claims.

Advance relative to prior literature:

The major novel findings of the paper are already summarized above. There is some recent work done on the subject of branch mechanics by the authors (Ghasemi et al 2024, PMID: 38277459) and others (Pandit et al 2020 PMID: 32461373), but the focus of the present work is clearly unique and the there is plenty of novel insight.

Audience and impact:

Primary audience: specialists in cytoskeleton dynamics, in vitro reconstitution single molecule biophysics, and mechanobiochemistry. Secondary: researchers in cell motility, morphogenesis and mechanobiology, physicists working on active matter and modelers studying force producing and load-bearing biopolymer networks. The results and analysis framework should inform quantitative models of branched network turnover under load and the interpretation of regulatory factor action in vivo and in cells.

Reviewer expertise:

Actin dynamics; biochemical reconstitution; single molecule approaches; biophysics.

3. How much time do you estimate the authors will need to complete the suggested revisions:

Estimated time to Complete Revisions (Required)

(Decision Recommendation)

Between 1 and 3 months

4. *Review Commons* values the work of reviewers and encourages them to get credit for their work. Select 'Yes' below to register your reviewing activity at Web of Science Reviewer Recognition Service (formerly Publons); note that the content of your review will not be visible on Web of Science.

Web of Science Reviewer Recognition

No

Review #2

1. Evidence, reproducibility and clarity:

Evidence, reproducibility and clarity (Required)

Xiao et al examine the molecular events occurring when Arp2/3 complex-mediated actin filament branches are removed from mother actin filaments. They do this using microfluidics assay with purified proteins combined with single filament TIRF imaging of branched actin filaments with distinct fluorescent labels. The contribution of different nucleotide states of Arp2/3 complex are tested in conjunction with the relationship force exerted on the branches and regulatory protein involvement from GMF and cortactin. The data seem comprehensive and highly quantified in response to concentration, force, fraction of branches and survival times and branching rates. They find that ADP-BeFx and high phosphate concentrations (leading to the ADP-Pi state) leads to a slower debranching rate at a given level of force applied. The ability to rapidly switch the buffer gives powerful information about response times of debranching compared with other actin remodelling events. They use renucleation experiments to determine that the previous debranching event most often occurs at the Arp2/3 complex/daughter interface, showing that filaments will be ready to re-branch in the stable ADP-Pi bound state. GMF addition allows debranching of the ADP state to occur at a lower force. Cortactin acts similarly to the ADP-Pi state to increase branch stability.

Specific comments

The pulling force on the branches seems to arise from different flow rates in the microfluidics. Viscous drag is mentioned and I can see there is methylcellulose in the buffer. It would be helpful to have the explanation of the conversion between flow and

force, even if it has been standard in previous work.

Pg 5 - what was the motivation to titrate phosphate? It seems a stretch that intracellular Pi levels are tuning branching inside cells more than protein-mediated control (GMF or cortactin) - can the authors evidence this at all?

Minor comments

- In the introduction, while the structural and mutagenesis evidence is clearly stated, in other cases a bit more detail would be helpful e.g. 'biochemical studies', which referred measurement of hydrolysis rates using radiolabelling
- Page 3 Figures shouldn't be referenced in the introduction
- Page 3 slip bond behaviour needs explanation
- Figure 1B seems to be a theoretical schematic which is superfluous
- Figure 4D is helpful, different weight lines might help even more to explain the dominant pathways

Referee cross-commenting

Rev1 sounds like the specialist here. I can't comment on their requests. Some similar points arise between the reviewers which need addressing.

2. Significance:

Significance (Required)

Taking a look at references 16 and 19, I do not find it clear what is achieved differently in the current work compared to these papers and what agrees and what disagrees. If it's a species difference I might expect the two species would be analysed side-by-side in this paper.

I'm not understanding how the authors can distinguish effects of adding phosphate and BeFx on Arp 2 and 3 compared to effects on actin. Importantly, are possible accompanying changes in the actin filament a confounding factor?

The paper is quite specialist to read and the advance appears to be incremental. My expertise is in molecular pathways to actin regulation outside the main area of the paper.

3. How much time do you estimate the authors will need to complete the suggested revisions:

Estimated time to Complete Revisions (Required)

(Decision Recommendation)

Between 1 and 3 months

4. *Review Commons* values the work of reviewers and encourages them to get credit for their work. Select 'Yes' below to register your reviewing activity at [Web of Science Reviewer Recognition Service] (formerly Publons); note that the content of your review will not be visible on Web of Science.

Web of Science Reviewer Recognition

Yes

Review #3

1. Evidence, reproducibility and clarity:

Evidence, reproducibility and clarity (Required)

Please find enclosed the review of the manuscript "Inorganic phosphate in Arp2/3 complex acts as a rapid switch for the stability of actin filament branches" by Xiao et al.

The authors provide a detailed investigation of how the nucleotide bound to the Arp2/3 complex affects branch stability under flow force. From a kinetic perspective, this is an elegant study with generally high-quality data, although some conclusions rest on assumptions rather than direct experimental evidence.

A key question concerns the physiological relevance of these findings. For instance, the concept of branch regrowth may not be applicable in cellular contexts, since forces by actin polymerization would displace existing branches away from sites where they generate this active forces. The authors should clarify the relevance of regrowth during active force generation by branched networks.

Additionally, all experiments employ flow conditions that branches would probably not experience in cells-notably, the flow direction in the cellular context would be reversed. Altering the flow direction relative to the branches could affect not only the relationship between flow rate and branch stability, but potentially other system properties as well.

2. Significance:

Significance (Required)

Furthermore, the study appears not to account for the mother filament (particularly its nucleotide state) or the actin subunit bound to the Arp2/3 complex. The authors should discuss why their interpretation focuses exclusively on the Arp2/3 complex rather than on the actin filaments or Arp2/3-bound actin subunit.

An important concern involves the use of KPi (inorganic phosphate). Based our experience, KPi appears to have effects beyond simply impacting nucleotide state-actin filaments seem to assemble differently in the presence of KPi. The authors should exercise caution in their interpretation of KPi-based experiments.

Overall, while the technical quality and kinetic analyses are state-of-the-art, relating this work to physiological contexts remains challenging, and some conclusions appear overstated.

3. How much time do you estimate the authors will need to complete the suggested revisions:

Estimated time to Complete Revisions (Required)

(Decision Recommendation)

Cannot tell / Not applicable

4. *Review Commons* values the work of reviewers and encourages them to get credit for their work. Select 'Yes' below to register your reviewing activity at Web of Science Reviewer Recognition Service (formerly Publons); note that the content of your review will not be visible on Web of Science.

Web of Science Reviewer Recognition

No

Full Revision

Manuscript number: RC-2025-03126
Corresponding author(s): Antoine, Jégou, Guillaume Romet-Lemonne

1. General Statements

Dear editor,

We are submitting a fully revised version of our manuscript in light of the points raised by the reviewers that we hope to have addressed convincingly below.

As you will see, reviewers are positive about the impact of our manuscript, especially reviewer #1 who seem to have the best expertise in the topic we tackled:

- Reviewer #1 states that "**This is a compelling and carefully executed study [...]**", "**The experimental design is elegant and experiments have been carried out to a masterful standard.**"; "**There are very few labs in the world that could have achieved this feat!**"; "**the focus of the present work is clearly unique and there is plenty of novel insight**" ; and "**Overall, the clearly work merits publication with high priority after revision [...].**"
- For reviewer #3, this paper is "**From a kinetic perspective, this is an elegant study with generally high-quality data**".

We have addressed the reviewers' main concern on the fact that "some conclusions rest on assumptions rather than direct experimental evidence" (reviewer #3) by performing additional experiments:

- We have measured the detachment of surviving-Arp2/3 complexes from mother filament using fluorescently labelled Arp2/3. This experiment was key to confirm that Arp2/3 in the ADP-BeFx state is extremely stable on mother filaments, and to measure Pi release from surviving Arp2/3 complexes.
- We have performed All-atom molecular dynamics (MD) simulations to show that Arp2, but not Arp3, is able to rapidly bind and release inorganic phosphate by having its putative backdoor opened.
- We have performed numerous control experiments to reinforce our conclusions that the nucleotide state of Arp2/3 is the dominant factor contributing to the mechanical stability of branch junctions, rather than the biochemical and mechanical states of the actin filament.
- We have carefully re-assessed our interpretation of the results for branch stability in the presence of cortactin.

Furthermore, as requested by reviewer #2 and #3, we have substantially improved our discussion on the physiological relevance of the nucleotide state of the Arp2/3 complex at branch junctions and how it acts upstream of the regulatory proteins GMF and cortactin.

We believe that our manuscript, now in its revised version, should alleviate the reviewers' concerns. We expect it should represent a significant advance in our understanding of how inorganic phosphate controls the mechanical stability and biochemical regulation of branched filaments, with important consequences on how actin networks can rapidly adapt to external stimuli.

2. Point-by-point description of the revisions

We thank the three reviewers for their critical comments that allowed us to conduct new experiments in order to substantially improve our revised manuscript. Please find below our point-by-point responses in blue.

Reviewer #1

Evidence, reproducibility and clarity

1) Summary

This study investigates the mechanochemistry of Arp2/3-mediated branched actin networks at the level of individual branch junctions under load. Using microfluidic single-filament/branch force assays (including constant-force flow and open-chamber imaging) the authors quantify debranching, re-nucleation, and mother- vs daughter-interface stability across nucleotide states of Arp2/3 (ADP-Pi, ADP, and an ADP-BeFx proxy for ADP-Pi). They further test effects by two branch regulators (GMF and cortactin). Key findings include: (i) ADP-Pi and ADP complexes share similar force dependence but differ markedly (~20×) in intrinsic dissociation rate; (ii) phosphate turnover on the Arp2/3 complex is rapid ii) affinity for Pi drops when Arp2/3 loses its daughter filament; (iii) quantification from model fits uncovers large stability differences between daughter and mother interfaces of the Arp2/3 complex; (iv) extraordinary high stability of ADP-Pi-like Arp2/3 on the mother filament; and (v) distinct effects of GMF and cortactin on force-dependent stability. Overall, the work combines technically demanding measurements with mechanistic modeling to probe how nucleotide state and regulatory factors tune branch mechanics.

2) Major comments:

1. Low force kinetics and completeness of survival curves (Figure 1). "For all forces, the surviving curves exhibited a clear single exponential behavior...." While the data can be fitted to monoexponential decay curves, data at low forces is clearly incomplete. >90% of branches have

not dissociated by the end of the experiment. For the particular data shown in 1C (F00nN, n=60 total branches) it means that the time information is coming from <6 observations, which is rather low for the single molecule field. I am slightly worried by this point, since the debranching rates under ADP-Pi conditions at zero force, are even by one magnitude slower. Yet, no raw data is shown. Given that the dissociation rate at low forces is a contentious point, the authors should show the raw data and the corresponding fits. At present, they only show an experimental scheme and images for these "open chamber" assay (Fig S2). Ideally, they would image for much longer than 900s with lower sampling time in those assays, to firmly establish that 20-fold difference also holds at 0 force.

Essential; experiment might already be performed. Otherwise straightforward to do (weeks time).

In figure 1B, we indeed show a Survival curve for ADP-Arp2/3 complex branch dissociation at 0 pN up to 900 seconds. As now shown in updated supp figure S2, the data was in fact acquired for at least 5000 seconds for ADP-Arp2/3 and ADP-Pi states (N=2 repeats for each condition, with n = 60 and 90 branches for ADP-Arp2/3 branches, and 90 and 132 branches for ADP-Pi-Arp2/3 branches). The debranching rates reported in the initial submission were already obtained by fitting the surviving curves over the whole duration of the experiments.

2. Stability Analysis (Figure 4). I can follow much of the arguments presented in the stability analysis of the daughter vs mother interfaces, which is in principle extremely interesting! However, there are some concerns here:

i) The authors emphasize the zero force ratio derived from fits (which is linked to the stability difference of the two interfaces in the absence of force) despite this being only weakly constrained by data. Intuitively in the model, the stability difference should grow to very large values as the re-nucleation ratio approaches 1 at low force. This combined with the noise in the data poses an issue in my opinion. Looking at the data and the error margin, I think that the authors cannot state with high confidence that there is a real difference between the relative stability of the daughter and mother interfaces between the two nucleotide states of the complex.

Essential; analysis and textual revision only

We thank the reviewer for this comment. The difference in stability between the two interfaces is strongly constrained by the shape of the branch renucleation ratio versus force curve, and its value at 0 pN. This is illustrated in the figure shown below (new Supp Fig. S8), showing the

dissociation rates of the two interfaces (in 'dashed' and 'point-dashed' style) that contribute to the overall debranching rate in each nucleotide condition. Despite the limited force range at which we probed the debranching rate, the branch renucleation ratio curve informs us on which interface is the weakest, and how this evolves with force.

We have assessed the confidence intervals of the parameters obtained from the fits, taking into account the error bars on our experimental datapoints. It seems to indicate that the simultaneous fits of the debranching rate and the branch renucleation ratio curves indeed constrain the parameters quite strongly. These confidence intervals are now reported in the main text and in the summarizing table.

We have repeated branch renucleation experiments for ADP-BeFx- and ADP-Pi-Arp2/3 complex branches (see new figure 4C&D, and our response to the next point). We believe these new measurements allow a better assessment of the relative stability between the two interfaces for Arp2/3 complex branch junctions in the ADP-BeFx state.

Still, we agree with the reviewer that the dispersion of the experimental data does not allow us to have a strong confidence on the crossover force and relative stability difference of the interfaces. Therefore, we have slightly toned down the way we present and discuss the differences in stability when comparing the two nucleotide states.

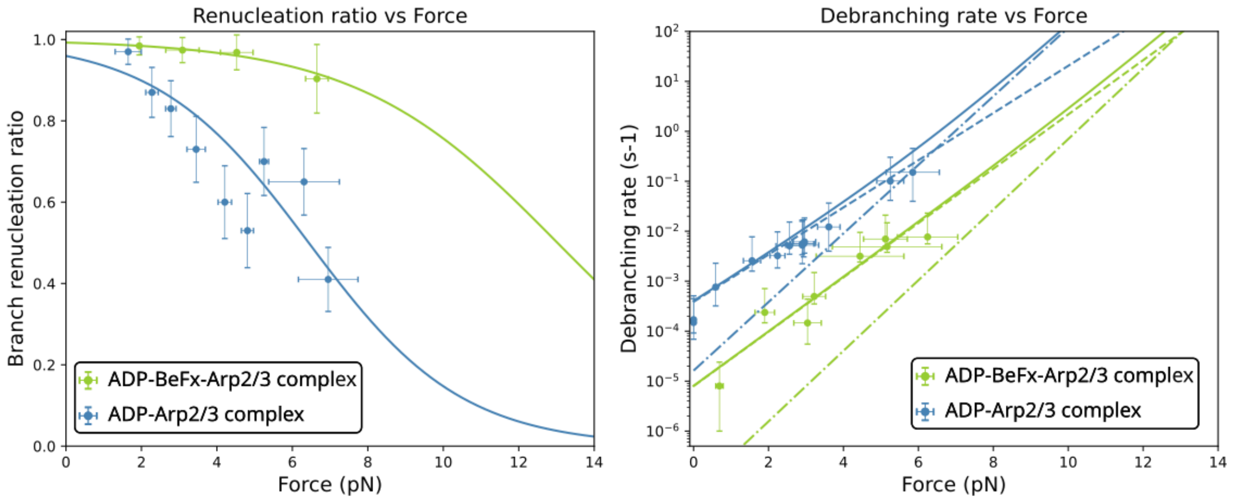

ii) For ADP-Pi, the renucleation ratio essentially remains flat over the measured force range. Hence, the data can only provide little leverage to estimate both the zero force ratio and, more importantly, the differential distance to the transition state in the slip-bond model in my opinion, which will show in the crossover force. Consequently, the quoted ">100×" stability difference at F=0 and the crossover force >20pN are driven largely by extrapolation rather than direct constraint by data. Given the high number of free parameters in the model, I would anticipate that several crossover forces and differential distances might explain the data nearly equally well. Instead of loosely reporting exact number from fits, I would have hoped for some sort of sensitivity analysis, for instance relying on profile likelihoods. Also parameter values could be reported as bounds (e.g crossover force ≫ measured range) rather than precise point estimates. This issue re-occurs (albeit not as drastically) for the cortactin experiments (Figure 6).

Essential; analysis and textual revision only

As mentioned in our response to the previous point, we have repeated renucleation experiments for ADP-BeFx- (and also for Arp2/3 complex branches in the presence of 50 mM Pi) (see new figure 4C&D) to better characterize the differential distance between to the transition force. The crossover force for the ADP-BeFx state is now 13.5 pN and the ratio of the stability between the two interfaces is roughly 100 times.

We agree with the reviewer that the dispersion of the experimental data does not allow us to have a strong confidence on the crossover force and relative stability difference of the interfaces. We have thus toned down the way we report these values. We do believe though that the difference we report between the ADP and ADP-BeFx state appears to be significant and needs to be acknowledged.

As a side note, it has proven to be challenging to pull on branches at forces higher than 7 pN. To apply a large force on the branch junction, we need to have a high flow rate. In this case, it appeared that the height of the filaments (both mother and daughter filaments) above the surface seem to deviate from what we have established in our previous studies (Jegou et al, Nat. Comm. 2013 & Wioland et al, PNAS 2019). This may originate from the fact branched filaments have a more complex shape than an individual filament. Characterizing accurately the evolution of the branch height as a function of the flow rate and applied force would require quite extensive additional characterization, which, we believe, is beyond the current focus of this study on the stability of Arp2/3 complexes.

iii) One important expectation from the "two slip bond" model is that branch dissociation rates should not necessarily scale mono-exponentially as they mostly do over the accessible force

range of the paper. However, once the "minor" pathway of dissociation from the mother starts to dominate at high forces, rates become more force sensitive. This is nicely recaptured by the model fits in Figure S6 but deserves some explanation in the text. Otherwise, people will simply remember the "ADP-Pi is 20-fold more stable than ADP at all forces" message.

Essential; textual revision only

We now have rephrased the key sentences (in the Abstract and Results sections) to more clearly state that the debranching rate is not increasing mono-exponentially with force.

In the Abstract: “Remarkably, we find that branch junctions are over 30-fold more stable when the Arp2/3 complex is in the ADP-Pi rather than ADP state, and that force accelerates debranching with similar exponential factors in both states.”

In the Results section: “The debranching rate seems to increase exponentially with the applied pulling force, in the range of 0 to 6 pN (Fig. 1F; see more refined analysis below). This behaviour is predicted by the Bell-Evans model for a slip bond.”

iv) One important prerequisite for the model is that isolated Arp2/3 complexes (without a daughter filament) should dissociate with equal rates from mother filaments at all flow rates. Since the Arp2/3 complex prefers mother filament curvature, forces experienced by the mother might change its off-rate. It would be good to refer to this assumption in the text and experimentally verify it. I could not find it in the paper nor in Ghasemi et al 2024.

Essential; simple experiment (a weeks time).

We thank the reviewer for this important comment.

First, we investigated whether the viscous drag force, applied on the ADP-Arp2/3 complexes which remain bound to mother filaments could affect their stability. We have performed branch renucleation experiments at different flow rates but with the same pulling force on branch junctions (average force 3.9 pN) by adapting the length of the daughter filament. As shown in new supp. figure S11 (shown below), we did not observe any significant differences between ‘low’ and ‘high’ flow rates. If the off-rate of the surviving Arp2/3 was significantly affected by the flow, this would have led to a variation of the renucleation ratio with the flow rate.

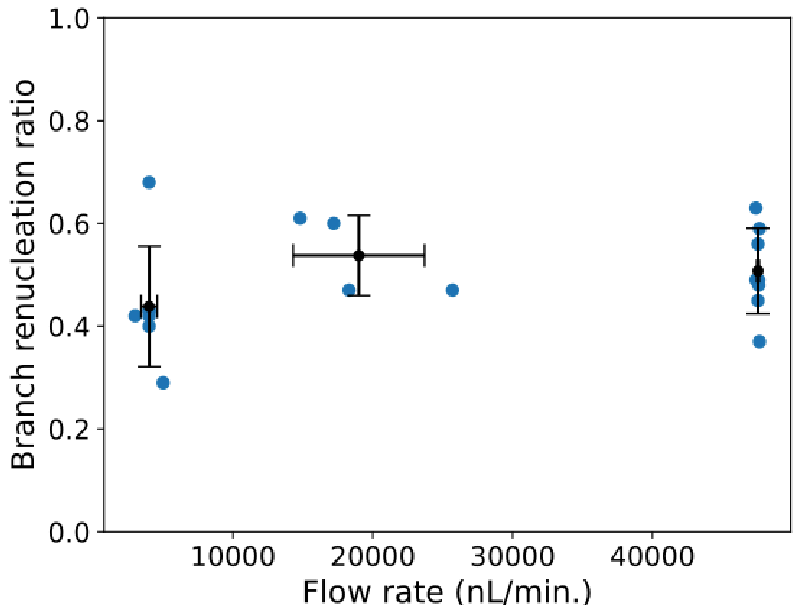

Second, we have investigated the impact of the tension experienced by the mother filament at the location of the branch junction for ADP-Arp2/3 complex branches, with the same pulling force on the branches (average 4.1 pN pulling force on branches). We have quantified the debranching rate from three groups of branches depending on their position along mother filaments. As shown in new supp. figure S12 (shown below), we can observe a small trend, where the debranching rate decreases with the tension on the mother filament at the branching point.

Doubling the tension on the mother filament from 15 to 30 pN decreases the debranching rate by a third. Though, pairwise logrank tests performed between the survival fractions of the three binned groups do not report any statistical significant difference (all p values > 0.05). One possible explanation for this is the height of the mother filament in the microfluidics flow that increases linearly from the anchoring point to the free barbed end. As a consequence the pulling force on the branches will be higher, as branches experience faster flows.

For these same groups, upon branch dissociation, all remaining-bound Arp2/3 complexes are exposed to the same flow rate; the branch renucleation ratios were similar. Thus branch renucleation ratio seems to not significantly depend on the tension experienced by the mother filament at the branching point.

Similarly, Pandit et al PNAS 2020, Extended figure S1, also reported no detectable impact of the mother filament tension on the debranching rate in their assay.

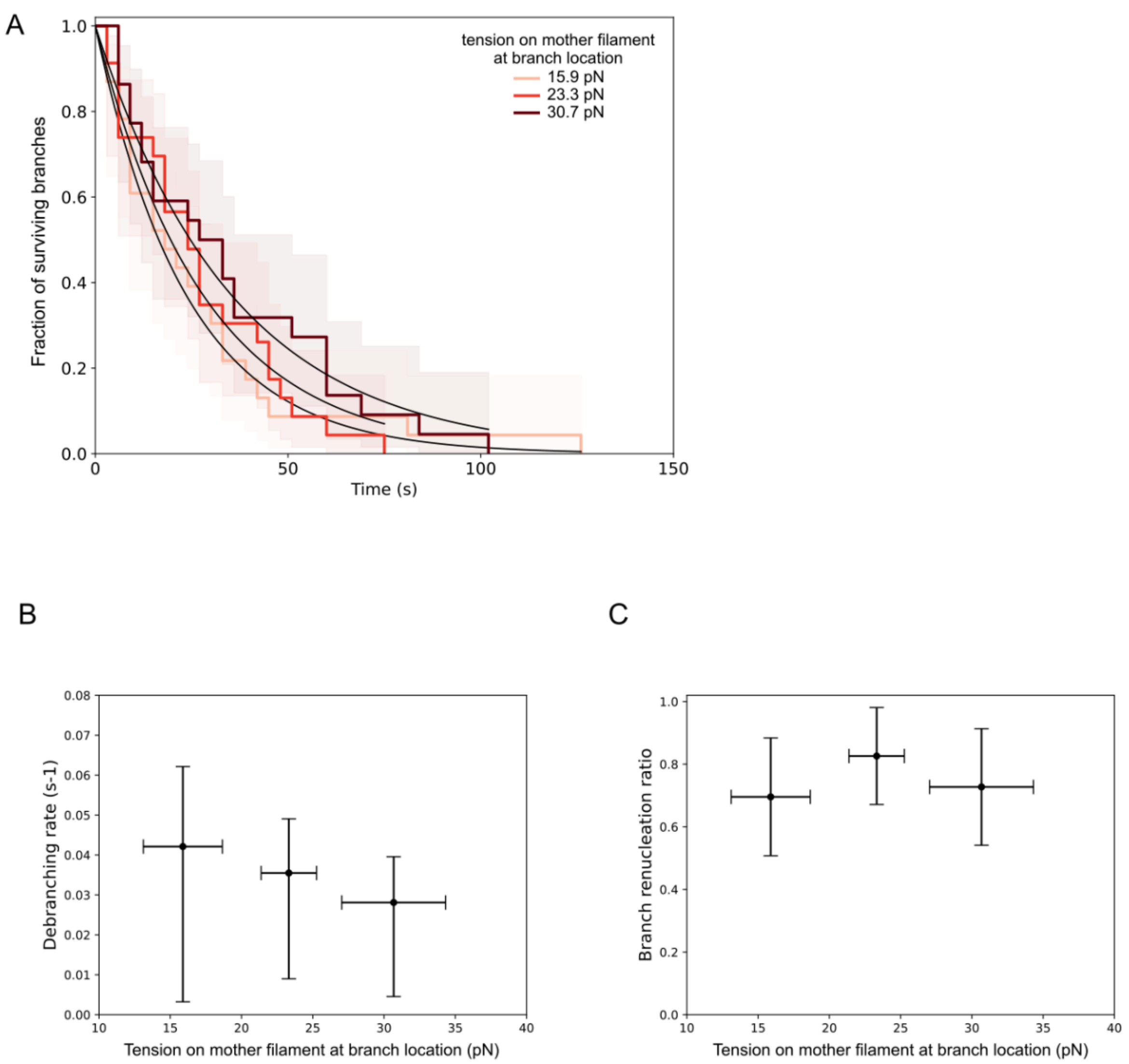

v) The force dependence of the branch re-nucleation rate (Fig 3D) has been measured previously by the same group (Ghasemi et al). While the data in the older paper has not been fitted by a model, the trend of the data in the previous paper looks conspicuously different. Are there any explanations for this? I speculate that it might be related to actin and ATP not being saturated (low-force re-nucleation rate rarely exceeds 80%) in Ghasemi et al., but it would be good to know what the authors think about this.
Essential; textual revision only

This is a good point. We have plotted the data of the renucleation ratio from ADP-Arp2/3 complex from figure 1F of Ghasemi et al, Sc. Adv. 2024 (performed at 0.3 and 1 μM actin), together with the data of the current study from figure 4D (performed at 1.5 μM actin). We feel

this comparison could be of interest to the readers, and have thus integrated it in the manuscript as new supp. figure S13 (shown below).

As expected, the branch renucleation ratio is lower with lower concentrations of actin. The experimental data points from Ghasemi et al are similarly well fitted by the branch renucleation function obtained for 1.5 µM multiplied by a scaling parameter, which reflects the fact that the branch renucleation ratio is actin concentration dependent (Fig. 6A in Ghasemi et al). This scaling parameter was the only free parameter of those fits.

Since the branch renucleation ratio depends on the actin concentration as follows, $\frac{0.97.k_{on}.([actin]-C_c)}{k_{on}.([actin]-C_c)+k_{off}^{ATP-Arp2/3}}$, with k_{on} = 3.4 µM^{-1}.s^{-1} and k_{off} $^{ATP\text{-}Arp2/3}$ = 0.66 s^{-1} from (Ghasemi et al. 2024), the scaling parameter obtained by the fits give estimates of the actin concentration in these experiments, of 0.6(±0.05) and 0.9(±0.2) µM for the experiments performed at 0.3 and 1 µM respectively in (Ghasemi et al. 2024).

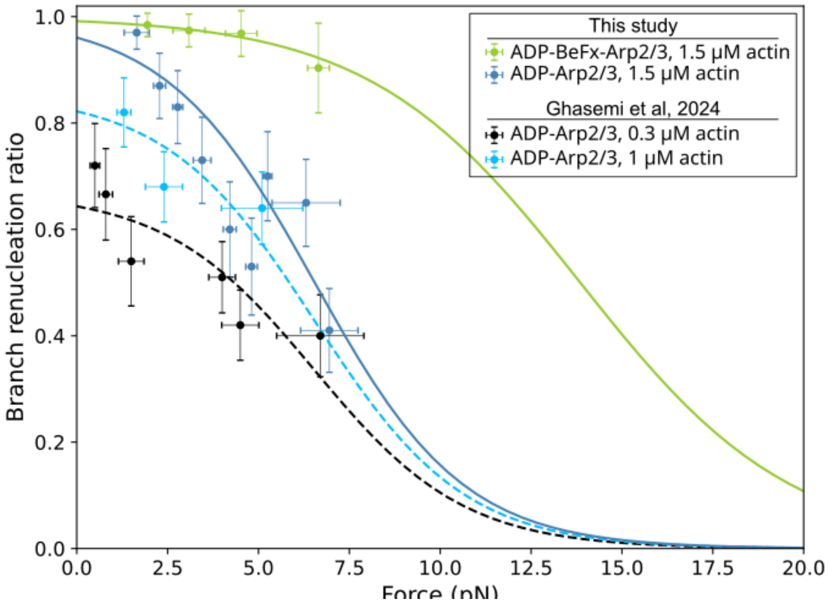

3. Stability of the authentic ADP-Pi-Arp2/3 complex on the mother filament. The extraordinary stability of the isolated ADP-BeFx-Arp2/3 complex on mother filaments is surprising, especially considering that both ATP and ADP states are much more labile (Ghasemi et al 2024). I would recommend repeating this experiment in the authentic ADP-Pi state with labelled Arp2/3 complexes as a more direct readout, even if this would require working with very high phosphate concentrations.

Essential; simple experiment (a weeks time).

We have followed the recommendation of the reviewer and have performed new experiments using fluorescent Arp2/3 complexes for ADP, ADP-BeFx and ADP-Pi states, now displayed in new figure 5C (also shown below).

For fluorescent Arp2/3 complexes remaining bound to the mother filament, the Arp2/3 complex - mother filament interface is ~ 100 times more stable in the ADP-BeFx state (0.0046 s^{-1}) compared to the ADP state (0.56 s^{-1}). We also assessed the dissociation of surviving ADP-BeFx-Arp2/3 complexes using unlabelled Arp2/3 complexes (previously in figure 4B, repeated experiment shown in new supp. figure S10), which also indicates a remarkable stability.

The dissociation curve of surviving Arp2/3 complexes in the presence of 50 mM Pi and 200 µM ATP in solution reflects the mixture of Arp2/3 dissociating in the ADP/ATP state and ADP-Pi-Arp2/3 that can either dissociate in the ADP-Pi state or lose their Pi and dissociate in the ATP state. Despite the presence of 50 mM Pi, the rate at which ADP dissociates and ATP reloads rate is much faster than Pi binding. Fitting this survival curve with a function that accounts for the initial double populations and the evolution of the ADP-Pi population (see Methods) gives a good estimate of the Pi release rate.

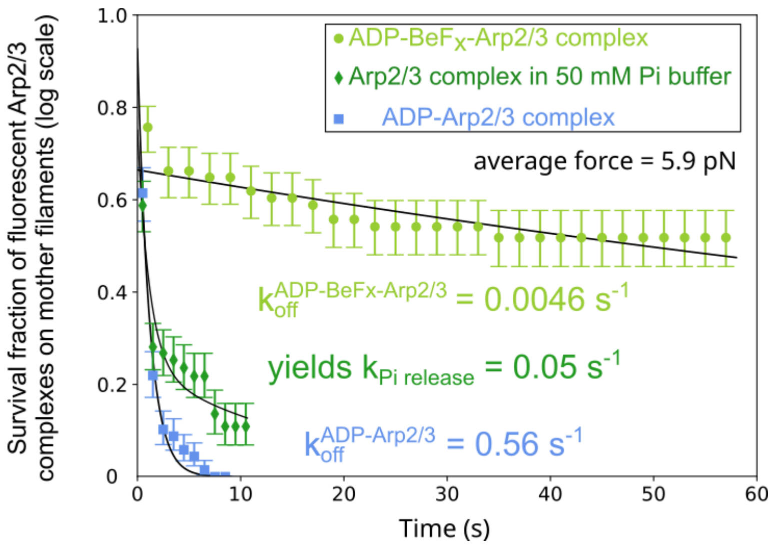

OPTIONAL: Further, but beyond the scope of the present paper, would be titrating phosphate in these experiments, which would even allow the authors to independently verify the reduced Pi affinity for Arp2/3 in the mother filament. Of note, this affinity difference is needed to satisfy detailed balance in the reaction scheme (Fig 4 D)!

We thank the reviewer for this suggestion. High concentrations of phosphate in the buffer renders glass surfaces quite sticky in our assays. We've tried several different passivation strategies (BSA, PLL-PEG, K-casein, …) but none gave satisfactory results. So titrating phosphate, by going beyond 50 mM phosphate, proved to be quite challenging.

Detailed balance, considering the two possible routes connecting the ADP-Pi-Arp2/3 complex branch junction state and the surviving ADP-Arp2/3 complex state, can be written as $K_{Pi\ rel.}^{branch\ junction}$. $K_{debranching}^{ADP\text{-}Arp2/3}$ = $K_{debranching}^{ADP\text{-}Pi\text{-}Arp2/3}$. $K_{Pi\ rel.}^{surviving\ Arp2/3}$. Some of these affinity constants are not known, because of the inability to determine reverse reactions rates such as the rebinding of a daughter filament to a surviving Arp2/3. It is thus hard to determine how the affinity of Pi for Arp2/3 complex changes between Arp2/3 complexes at branch junctions and surviving Arp2/3 complexes on mother filaments.

While we cannot determine the affinity constant of Pi for a surviving Arp2.3 complex, our data indicates that the dissociation rate of Pi is higher from Arp2/3 complexes at branch junction (k_{off} = 0.21 s^{-1}) than from surviving Arp2/3 complexes (k_{off} = 0.05 s^{-1}). This unexpected finding indicates that surviving Arp2/3 complexes adopt a conformation where the nucleotides are

readily exchanged, but where the ‘back door’ for Pi release is less open. We now discuss this point in our revised manuscript.

4. Importance of "surviving" ADP-Pi-Arp2/3 complexes. The authors show a) rapid turnover of Pi on the ADP-Arp2/3 complex in both branch- or mother filament-bound state and b) the lowered Pi affinity of the latter. Nonetheless, they emphasize the importance of long-lived "surviving" ADP-Pi bound complexes on the mother (even stated in the abstract). I understand that this fraction shows under some experimental conditions (BeFx), but unless I am missing something, most complexes should rapidly lose their phosphate and either exchange nucleotide or dissociate from the mother under physiological conditions. Please clarify or tone done.

Essential; textual revision only

We thank the reviewer for their remark. We have tried to clarify this aspect in the manuscript.

As shown now with the departure rate of fluorescent surviving Arp2/3 complexes together with branch renucleation data, we show that surviving ADP-Pi-Arp2/3 complexes are quite stable on mother filaments, because they detach and release their Pi slowly, such that branch regrowth will occur provided there is actin in solution. In the absence of actin monomers, as the reviewer correctly points out, the surviving ADP-Pi-Arp2/3 will predominantly release its Pi and thus become a surviving ADP-Arp2/3 complex. We have modified the text to avoid any confusion.

5. GMF mechanism. The authors claim that GMF "...accelerates the departure of the surviving Arp2/3 complex from the mother...". I assume that they infer this from decrease in the re-nucleation ratio. However, alternatively GMF could simply dwell on the complex, inhibiting re-nucleation without promoting dissociation from the mother. The authors should either monitor Arp2/3 dwell times directly to discriminate between these possibilities or be more cautious in their conclusions.

Essential; simple experiment (a weeks time) or textual revision.

In Ghasemi et al. Sci. Adv. 2024, we examined the departure of Arp2/3 from the mother filament after GMF-induced debranching using fluorescent Arp2/3. Most of the fluorescent Arp2/3 dissociated from mother filaments within the same frame as the branch, i.e. within 0.5 seconds after the debranching event, and none were visible after another second . This could be due to Arp2/3 departing with the branch or an accelerated departure after branch dissociation. In any case, this rules out the possibility that GMF would dwell on the surviving complex for a substantial amount of time without promoting dissociation from the mother.

In the present manuscript, we now show that increasing the ATP concentration 10-fold (from 0.2 to 2 mM) is sufficient to restore the branch renucleation ratio to its level without GMF. This shows that GMF does not cause Arp2/3 to leave with the branch, but rather that it (also) acts on the surviving Arp2/3 complex, in a way that is countered by high concentrations of ATP. More specifically, it suggests that GMF accelerates the departure of the surviving ADP-Arp2/3 complex, either directly and by hindering the reloading of ATP, and that GMF does not affect the surviving Arp2/3 complex once it has reloaded ATP.

We now discuss these two non-mutually exclusive possibilities for the accelerated dissociation of the surviving ADP-Arp2/3 complex in the manuscript.

6. Cortactin mechanism and the "leash model". I must say that the cortactin data are the most puzzling part of the paper and hard to reconcile with what we know from structure. I was hoping to find some of this resolved in the discussion. However, I do not understand the "leash model" in the discussion section for cortactin-mediated branch stabilization: "This would explain the observed increase in branch survival compared to the absence of cortactin. As the pulling force is increased, this rebinding mechanism becomes less efficient." According to my understanding of the data, this is opposite to what happens. Cortactin only stabilizes the labile interface at elevated forces! Some re-writing might help here.

Essential; textual revision.

We thank the reviewer for having us think more thoroughly about the model we initially proposed. We now believe that our 'leash' mechanism is not able to fully recapitulate our observations in a simple and satisfactory manner.

We now propose a much simpler model, where the binding of cortactin to the Arp2/3 complex at the branch junction simply changes the energy landscape of the Arp2/3-daughter interface without the need to invoke a rebinding of the daughter filament upon branch departure. We have updated our interpretation of the data in the Discussion section accordingly.

Overall, our results on the impact of cortactin on branch renucleation highlights a surprising behaviour that would require further investigation to fully decipher the underlying molecular mechanism.

3) Minor comments

Organization:
- I do not want to impose on how to best tell the story, but I felt that Fig1 A-D and Fig 2 A-B belong to one logical unit (nucleotide dependence), whereas Fig 1 E-F and Fig 2 C belong to the other (Pi binding and exchange). Perhaps consider re-organizing to streamline presentation?

We thank the reviewer for their suggestion. We agree that it flows more naturally as suggested, and have made the changes! Thank you.

Semantics/Typos:
- Abstract: „... ADP-Pi and ADP-Arp2/3 detach with the same exponential increase as a function of force...". Increase should refer to the dissociation rate, which should be added to the sentence.

We have corrected this.

- Results page 8: "...and the majority of Arp2/3 complexes detach from the mother filament while remaining bound to the branch at the debranching time." "Branch" should likely be daughter here, as there is no branch after dissociation of either interface.

We have corrected this, thank you.

- Results page 13: "Exposing ADP-BeFx-Arp2/3 complex branch junctions to a saturating amount of GMF...". It is strange to imply saturation, because GMF likely simply does not bind to the complex in this nucleotide state with appreciable affinity. Suggest to change to "high".

We have made the changes accordingly.

- Discussion page 18: "Moreover, in mammalian Arp2/3, His80 in Arp3 (corresponding to His73 in mammalian actin) is not methylated, and corresponds to residue N77 in Arp3, which is also not modified." N77 likely belongs to Arp2?

We have made the changes accordingly.

- Discussion page 19: "We showed that Pi affinity for Arp2/3 complexes at branch junctions is around 3.7 mM (Fig. 1), a value which lies within the reported 1-10 mM Pi concentration measured in the cytosol in different mammalian cell types". Notably, this is not too different from

F-actin, which should be mentioned. By this measure alone, free inorganic phosphate could also directly regulate actin filament stability!

We now mention this and discuss that intracellular Pi can also impact actin filament nucleotide state.

Future interest (non essential):
- It would be utterly exciting (but beyond current scope) to quantify how instantaneous debranching rates evolve for naturally aging branches starting from ATP-Arp2/3 complexes!

We thank the reviewer for this remark. It is indeed quite beyond the scope of the current study, as this would require a way to probe ATP-Arp2/3 complex branches while daughter filaments are still quite short (so pulling on them is difficult). An interesting alternative could be to use ATP analogs, such as App-NHp (aka AMP-PNP), to stabilize this state. However, some studies have mentioned that App-NHp is not very stable.

Significance

General assessment:

This is a compelling and carefully executed study that delivers a clear mechanistic framework for how Arp2/3 branch junctions fail and re-form under load. The central strength is the tight integration of state-of-the-art reconstitutions with careful and original kinetic analysis. The experimental design is elegant and experiments have been carried out to a masterful standard. The figures are clear, the statistics are appropriate with some exceptions as detailed above. There are very few labs in the world that could have achieved this feat!

A few aspects could be further strengthened, most notably the explanation and application of the "two slip bond" model as well as slightly more restraint in speculating around specific regulatory mechanisms. However, these are minor refinements that do not detract from the important contributions of the paper.

Overall, the clearly work merits publication with high priority after revision; most requested changes are textual/analytical with very few targeted experiments, which would substantially strengthen core claims.

We thank the reviewer for their positive evaluation of our manuscript. We hope that our responses to the detailed points above, along with the corresponding revisions of the manuscript, will alleviate their concerns.

Advance relative to prior literature:
The major novel findings of the paper are already summarized above. There is some recent work done on the subject of branch mechanics by the authors (Ghasemi et al 2024, PMID: 38277459) and others (Pandit et al 2020 PMID: 32461373), but the focus of the present work is clearly unique and the there is plenty of novel insight.

Audience and impact:
Primary audience: specialists in cytoskeleton dynamics, in vitro reconstitution single molecule biophysics, and mechanobiochemistry. Secondary: researchers in cell motility, morphogenesis and mechanobiology, physicists working on active matter and modelers studying force producing and load-bearing biopolymer networks. The results and analysis framework should inform quantitative models of branched network turnover under load and the interpretation of regulatory factor action in vivo and in cells.

Reviewer expertise:
Actin dynamics; biochemical reconstitution; single molecule approaches; biophysics.

Review COMMONS

Reviewer #2 (Evidence, reproducibility and clarity (Required)):

Xiao et al examine the molecular events occurring when Arp2/3 complex-mediated actin filament branches are removed from mother actin filaments. They do this using microfluidics assay with purified proteins combined with single filament TIRF imaging of branched actin filaments with distinct fluorescent labels. The contribution of different nucleotide states of Arp2/3 complex are tested in conjunction with the relationship force exerted on the branches and regulatory protein involvement from GMF and cortactin. The data seem comprehensive and highly quantified in response to concentration, force, fraction of branches and survival times and branching rates. They find that ADP-BeFx and high phosphate concentrations (leading to the ADP-Pi state) leads to a slower debranching rate at a given level of force applied. The ability to rapidly switch the buffer gives powerful information about response times of debranching compared with other actin remodelling events. They use renucleation experiments to determine that the previous debranching event most often occurs at the Arp2/3 complex/daughter interface, showing that filaments will be ready to re-branch in the stable ADP-Pi bound state. GMF addition allows debranching of the ADP state to occur at a lower force. Cortactin acts similarly to the ADP-Pi state to increase branch stability.

Specific comments

The pulling force on the branches seems to arise from different flow rates in the microfluidics. Viscous drag is mentioned and I can see there is methylcellulose in the buffer. It would be helpful to have the explanation of the conversion between flow and force, even if it has been standard in previous work.

We apologize if this was unclear: in microfluidics experiments, the buffer does not contain methylcellulose. Methylcellulose is only used for ‘open chamber’ experiments, where no force is applied to Arp2/3 branches, to maintain them in the TIRF field of excitation (Figure S2).
To better clarify the conversion between flow and force, we have rephrased and extended the Methods section to explain how the force on the branch junction is computed based on the local flow velocity and the length of the daughter filament.

Pg 5 - what was the motivation to titrate phosphate? It seems a stretch that intracellular Pi levels are tuning branching inside cells more than protein-mediated control (GMF or cortactin) - can the authors evidence this at all?

We are not claiming that the level of Pi plays a stronger regulatory role than proteins. We show that inorganic phosphate tunes the state of the Arp2/3 complex, which in turn modulates the action of regulatory proteins, such as GMF and cortactin.

Nonetheless, we do show that the contribution of inorganic phosphate is quite central as it can (1) strongly stabilize branch junctions (~30-fold decrease in the dissociation rate), and (2) tune

the activity of GMF and cortactin on Arp2/3 complexes at branch junctions as well as on the ‘surviving’ Arp2/3 complexes that remain bound to mother filaments.

We thus titrated phosphate and found that its impact on Arp2/3 complex stability is significant in the range of Pi concentration that is explored in cells. For the sake of completeness, and following a comment from reviewer #1, we now also mention the affinity of Pi for actin subunits in filaments in the Discussion, and discuss the impact of intracellular Pi on actin itself.

Minor comments
- In the introduction, while the structural and mutagenesis evidence is clearly stated, in other cases a bit more detail would be helpful e.g. 'biochemical studies', which referred measurement of hydrolysis rates using radiolabelling

We have made changes to more precisely define which biochemical assays were used in previous studies.

- Page 3 Figures shouldn't be referenced in the introduction

We have removed the references to the figures from the introduction.

- Page 3 slip bond behaviour needs explanation

We now explain the concept when first using this concept in the manuscript, as follows: “The debranching rate seems to increase exponentially with the applied pulling force, in the range of 0 to 6 pN (Fig. 1F; see more refined analysis below). This behaviour of accelerated debranching with the increase of the applied force is similar to the ‘slip bond’ concept, as predicted by the Bell-Evans model of the force-dependent lifetime of the interaction between two proteins”.

- Figure 1B seems to be a theoretical schematic which is superfluous

We suppose that the reviewer is actually referring to figure 3B of the initial manuscript, describing the energy potential of a molecular interaction as a function of the reaction coordinate. We agree with the reviewer that it is not absolutely required and we have removed it.

- Figure 4D is helpful, different weight lines might help even more to explain the dominant pathways

We have made modifications to the biochemical reaction scheme in this figure (now figure 5F in the revised version). We hope we succeeded in improving its readability. Since the different paths depend on mechano-chemical parameters, there is no real dominant pathway per se.

Referee cross-commenting

Rev1 sounds like the specialist here. I can't comment on their requests. Some similar points arise between the reviewers which need addressing.

Reviewer #2 (Significance (Required)):

Significance

Taking a look at references 16 and 19, I do not find it clear what is achieved differently in the current work compared to these papers and what agrees and what disagrees. If it's a species difference I might expect the two species would be analysed side-by-side in this paper.

We thank the reviewer for this important comment. The goal of our study was not to compare the behaviour of mammalian and yeast Arp2/3 complexes.

We now try to better explain that the motivation of the present work is to address how the nucleotide state of the Arp2/3 complex tunes actin branch mechanosensitive stability, and regulates interactions with well known Arp2/3 complex binding proteins. Most of the reactions are quantified here for the first time. Moreover, the experiments with branch junctions in different nucleotide states are done under controlled mechanical conditions, providing the first direct measurements of the force-dependence of the debranching reactions. Our detailed kinetic analysis of the full reaction scheme allows us to model the different binding interfaces of the Arp2/3 complex.

In addition, it is worth noting that:
1. Species matter and this is why ref 16 and 19 can give the impression to disagree on the ability to renucleate branches thanks to the stability of surviving Arp2/3 complexes on mother filaments.
2. In ref 16 (Pandit et al, PNAS 2020) species are mixed (yeast Arp2/3 and mammalian alpha actin from skeletal muscle), likely leading to a different behaviour compared to the only mammalian protein situation we examine in our current work. In particular, with mixed species one misses the ability to renucleate, as shown in our previous study Ghasemi et al (ref 19). However, since mixing species does not correspond to anything physiological, we do not think it is worth repeating these conditions alongside our experiments.
3. Further, the analysis carried out in ref 16 suffers from important limitations: the force was unknown (not calibrated) and the data was fitted by a model that compounded several reactions, providing only an indirect estimation of the rates, in particular at zero force. In contrast, we have worked with calibrated forces (including dedicated experiments at zero force) and we have carried out specific experiments to directly measure several rates.
4. In ref 19 (our earlier work) we did not investigate the impact of the nucleotide state of the branch junction at all, and we did not systematically measure the dissociation rates as a function of force.

Contrary to Pandit et al, we directly measure the difference in branch stability at zero force between ADP and ADP-Pi states and show that the ~ 30 fold difference holds true at all probed forces. Last, the force dependence of the branch renucleation success rate gives us crucial information on which of the two Arp2/3 complex interfaces ruptures first.

I'm not understanding how the authors can distinguish effects of adding phosphate and BeFx on Arp 2 and 3 compared to effects on actin. Importantly, are possible accompanying changes in the actin filament a confounding factor?

We have checked that the nucleotide state (ADP-BeFx and ADP-Pi versus ADP) of the mother and daughter filaments have no impact on branch stability:

- In the experiments shown in figure 2F, where the buffer condition to which branches are exposed is quickly changed from phosphate buffer to buffer without phosphate, we observe a rapid change of branch stability. Actin subunits at the branch junction are in F-actin conformation according to recent cyroEM observations (ref. Chavani et al, Nat Comm. 2024; Liu et al, NSMB 2024). These actin subunits, initially in the ADP-Pi state, are expected to age and become ADP with a rate of ~ 0.007 s^{-1} (ie half-time of 100 s; ref. Jegou et al, PLoS Biology 2011, Ooosterhert et al, NSMB 2023), a much lower rate than the observed change of the debranching rate (0.21 s^{-1}). This means that the debranching rate is independent of the nucleotide state of daughter and mother filaments.

- In new supp. Figure S4, we show that the debranching rate is similar for ADP-Arp2/3 complex branch junctions initiated from ADP- or ADP-BeFx-actin mother filaments.

- In new supp. Figure S9, we initially exposed branch junctions to a BeFx solution then monitored debranching and branch renucleation in our standard buffer (ie without BeFX or Pi). We observed multiple rounds of branch renucleation, the first with ADP-BeFx-actin daughter filaments, and the following with daughter filaments never exposed to BeFx. They all had the same debranching rates and renucleation success rates.

The paper is quite specialist to read and the advance appears to be incremental. My expertise is in molecular pathways to actin regulation outside the main area of the paper.

The results we present in this study are often unexpected, and some go counter long-standing assumptions. The regulation of Arp2/3-nucleated branches is of importance for the stability and the force-generating capabilities of many actin networks in cells. Last, most of the measurements that we present had never been done, mainly because experiments are difficult to achieve, and require specific tools to monitor several events while controlling the applied force.

Full Revision

We believe our results are of broad interest as they go counter long-standing assumptions. We have rewritten the text in several instances to convey our message more clearly.

Reviewer #3 (Evidence, reproducibility and clarity (Required)):

Please find enclosed the review of the manuscript "Inorganic phosphate in Arp2/3 complex acts as a rapid switch for the stability of actin filament branches" by Xiao et al.

The authors provide a detailed investigation of how the nucleotide bound to the Arp2/3 complex affects branch stability under flow force. From a kinetic perspective, this is an elegant study with generally high-quality data, although some conclusions rest on assumptions rather than direct experimental evidence.

We thank the reviewer for their positive feedback. We have improved our manuscript and performed important additional experiments to provide more direct experimental evidence of our conclusions.

A key question concerns the physiological relevance of these findings. For instance, the concept of branch regrowth may not be applicable in cellular contexts, since forces by actin polymerization would displace existing branches away from sites where they generate this active forces. The authors should clarify the relevance of regrowth during active force generation by branched networks.

We thank the reviewer for this comment. Our in vitro results indeed point to a previously unreported property of branched actin networks, i.e. the ability of Arp2/3 complexes to readily renucleate branches in the ADP-Pi state and that it does require reloading ATP within Arp2/3.

Branched actin networks, especially the lamellipodia or endocytotic patches, do exert active force thanks to actin polymerization of the individual branches at the forefront. Though, the whole actin network is exposed to stress, and the architecture of the network (inter-branch distance, crosslink between branches, …) presumably strongly impact its mechanical properties.

In the case of other types of branched actin networks, such as the actin cortex, myosin motor put the whole network under tension. Such pulling forces on actin branches, depending on the amplitude of the pulling force, can lead to branch regrowth, and network self-repair.

We have modified the text to make the physiological relevance clearer.

Additionally, all experiments employ flow conditions that branches would probably not experience in cells-notably, the flow direction in the cellular context would be reversed. Altering the flow direction relative to the branches could affect not only the relationship between flow rate and branch stability, but potentially other system properties as well.

We agree with the reviewer that in cells branches will not experience flow conditions similar to the ones we use in our in vitro assay. Nonetheless, in cells we expect mechanical stress on the

branch junction to be applied in all directions. In lamellipodia, the compressive force applied at the leading edge is expected to result in diverse local orientations of the force on individual branch junctions within the network (as explained in Lappalainen et al. Nat Rev MBC 2022). Also, branch junctions are found in the cell cortex, where they are exposed to pulling forces resulting from the action of myosin motors and crosslinkers on mother and daughter filaments.

This impact of the direction of the flow was addressed in our previous publication (Ghasemi et al, Sc. Adv. 2024, figure 2) and, to a lesser extent, by the lab of Enrique de la Cruz in Pandit et al, PNAS 2020 (ref. 16). We reported that flow direction has a minimal effect, if any, on branch dissociation rate and renucleation ratio.

Reviewer #3 (Significance (Required)):

Furthermore, the study appears not to account for the mother filament (particularly its nucleotide state) or the actin subunit bound to the Arp2/3 complex. The authors should discuss why their interpretation focuses exclusively on the Arp2/3 complex rather than on the actin filaments or Arp2/3-bound actin subunit.

We have checked that the nucleotide state (ADP-BeFx and ADP-Pi versus ADP) of the mother and daughter filaments has no impact on branch stability :

- In the experiments shown in figure 2F, where the buffer condition to which branches are exposed is quickly changed from phosphate buffer to buffer without phosphate, we observe a rapid change of branch stability. Actin subunits at the branch junction are in F-actin conformation according to recent cyroEM observations (ref. Chavani et al, Nat Comm. 2024; Liu et al, NSMB 2024). These actin subunits, initially in the ADP-Pi state, are expected to age and become ADP with a rate of ~ 0.007 s^{-1} (ie half-time of 100 s; ref. Jegou et al, PLoS Biology 2011, Ooosterhert et al, NSMB 2023), a rate much lower than the observed change of the debranching rate (0.21 s^{-1}). This means that the debranching rate is independent of the nucleotide state of daughter and mother filaments.

- In new supp. Figure S4, we show that the debranching rate is similar for ADP-Arp2/3 complex branch junctions initiated from ADP- or ADP-BeFx-actin mother filaments.

- In new supp. Figure S9, we initially exposed branch junctions to a BeFx solution then monitored debranching and branch renucleation in a regular buffer. We observed multiple rounds of branch renucleation, the first with ADP-BeFx-actin daughter filaments, and the following with daughter filaments never exposed to BeFx. They all had the same debranching rates and renucleation success rates.

An important concern involves the use of KPi (inorganic phosphate). Based our experience, KPi appears to have effects beyond simply impacting nucleotide state-actin filaments seem to

assemble differently in the presence of KPi. The authors should exercise caution in their interpretation of KPi-based experiments.

Concentration of KPi (up to 50 mM Pi) did not slow down barbed end elongation rate in our experiments.

Overall, while the technical quality and kinetic analyses are state-of-the-art, relating this work to physiological contexts remains challenging, and some conclusions appear overstated.

We have made changes in the discussion to try to more clearly relate our in vitro observations and conclusions with the cellular context where branch renucleation could have a strong impact on the architecture and mechanics of actin networks.

Re-reviews by the original reviewers:

Referee #1:

The authors have done an exemplary job comprehensively addressing a long list of points. Their thoughtful revision has significantly improved the paper. I strongly recommend its publication without further delay.

Only a minor semantic issue for improvement:

- page 7 results section: please refer to the "third phosphate" as the gamma phosphate.

Referee #3:

I have read the revised manuscript and the authors' replies to the previous comments. As I said before, and as Reviewers #1 and #2 also noted, the experiments are very well done. However, some of my concerns have not been fully addressed.

I am still not convinced that these findings matter in a biological context, and the authors' response did not change my view on this. They argue that "the whole network is exposed to stress," but this does not account for how branched actin networks actually work: the branches are connected to each other in a complex manner with different orientations, so for phosphate to meaningfully destabilize the network under stress, many branches would have to be under the same stress at the same time. This has not been shown and remains an assumption.

The authors also bring up myosin contractility as a relevant biological scenario, but myosin removes branches through a mechanism that does not necessarily depend on the nucleotide state of the Arp2/3 complex. Using this as support for the physiological relevance of the phosphate effect is therefore a far-fetched argument.

The experimental work is very well conducted, but the biological significance of the results seems limited, and the question of what is genuinely new compared to previous work - from this group and others - has not been satisfactorily answered.

If the other reviewers feel the paper is ready to publish, I will not object. That said, how this paper gets cited in the future - in the context of actin network mechanics - will ultimately reflect

whether these concerns were justified.

May 29, 2026

RE: JCB Manuscript #202605018T

Antoine Jégou
Institut Jacques Monod

Dear Dr. Jégou:

Thank you for submitting your revised manuscript entitled "Inorganic phosphate rapidly switches the stability of Arp2/3 induced actin branches" via Review Commons. We have editorially assessed your manuscript as well as the reviewers' comments and we would be happy to publish your paper in JCB pending final revisions necessary to meet our formatting guidelines (see details below).

In your final manuscript, we ask you to address the final editorial concerns (please see below, appended to this letter) via textual revisions.

To avoid unnecessary delays in the acceptance and publication of your paper, please read the following information carefully.

A. MANUSCRIPT ORGANIZATION AND FORMATTING:

Full guidelines are available on our Instructions for Authors page, http://jcb.rupress.org/submission-guidelines#revised.
Submission of a paper that does not conform to JCB guidelines will delay the acceptance of your manuscript.

1) Text limits: Character count for Articles is < 40,000, not including spaces. Count includes abstract, introduction, results, discussion, and acknowledgments. Count does not include title page, figure legends, materials and methods, references, tables, or supplemental legends.

2) Figures limits: Articles may have up to 10 main text figures.

3) Figure formatting: Scale bars must be present on all microscopy images, including inset magnifications. Molecular weight or nucleic acid size markers must be included on all gel electrophoresis. Aspect ratios of images may not be altered.

4) Statistical analysis: Error bars on graphic representations of numerical data must be clearly described in the figure legend. The number of independent data points (n) represented in a graph must be indicated in the legend. Statistical methods should be explained in full in the materials and methods. For figures presenting pooled data the statistical measure should be defined in the figure legends. Please also be sure to indicate the statistical tests used in each of your experiments (either in the figure legend itself or in a separate methods section) as well as the parameters of the test (for example, if you ran a t-test, please indicate if it was one- or two-sided, etc.). Also, if you used parametric tests, please indicate if the data distribution was tested for normality (and if so, how). If not, you must state something to the effect that "Data distribution was assumed to be normal but this was not formally tested."

**5) Abstract and title: The abstract should be no longer than 160 words and should communicate the significance of the paper for a general audience. The title should be less than 100 characters including spaces. Make the title concise but accessible to a general readership.

Given that your abstract exceeds the 160 word limit, we ask you to shorten the text, while keeping the editorial recommendations regarding the last sentence in mind.

**6) Materials and methods: Should be comprehensive and not simply reference a previous publication for details on how an experiment was performed. Please provide full descriptions in the text for readers who may not have access to referenced manuscripts. For example, in the "Proteins" paragraph of your methods section, you state: "Alpha skeletal muscle actin (UniProt P68135) was purified from rabbit muscle acetone powder following the protocol from (61) as described in (62)." You must remove this and write out the full methodology. The same goes for all other places in the methods where you refer to methods "...as previously described."

7) All antibodies, cell lines, animals, and tools used in the manuscript should be described in full, including accession numbers for materials available in a public repository such as the Resource Identification Portal. Please be sure to provide the sequences for all of your primers/oligos and RNAi constructs in the materials and methods. You must also indicate in the methods the source, species, and catalog numbers (where appropriate) for all of your antibodies. Please also indicate the acquisition and quantification methods for immunoblotting/western blots.

8) Microscope image acquisition: The following information must be provided about the acquisition and processing of images:
a. Make and model of microscope
b. Type, magnification, and numerical aperture of the objective lenses
c. Temperature
d. Imaging medium
e. Fluorochromes
f. Camera make and model
g. Acquisition software
h. Any software used for image processing subsequent to data acquisition. Please include details and types of operations involved (e.g., type of deconvolution, 3D reconstitutions, surface or volume rendering, gamma adjustments, etc.).

**9) References: There is no limit to the number of references cited in a manuscript. References should be cited parenthetically in the text by author and year of publication. Abbreviate the names of journals according to PubMed.

**10) Supplemental materials: There are strict limits on the allowable amount of supplemental data. Articles may have up to 5 supplemental figures. At the moment, you have 16 supplemental figures. While we will be able to give you a bit more space in this case, we will still need for you to reduce the count a bit, for example by combining some of the supplemental figures. Please be sure to correct the callouts in the text to reflect this change. Please also note that tables, like figures, should be provided as individual, editable files. A summary of all supplemental material should appear at the end of the Materials and methods section.

**11) eTOC summary: A ~40-50-word summary that describes the context and significance of the findings for a general readership should be included on the title page. The statement should be written in the present tense and refer to the work in the third person.

12) Conflict of interest statement: JCB requires inclusion of a statement in the acknowledgements regarding competing financial interests. If no competing financial interests exist, please include the following statement: "The authors declare no competing financial interests." If competing interests are declared, please follow your statement of these competing interests with the following statement: "The authors declare no further competing financial interests."

**13) ORCID IDs: ORCID IDs are unique identifiers allowing researchers to create a record of their various scholarly contributions in a single place. Please note that ORCID IDs are now *required* for all authors. At resubmission of your final files, please be sure to provide your ORCID ID and those of all co-authors.

14) A separate author contribution section following the Acknowledgments. All authors should be mentioned and designated by their full names. We encourage use of the CRediT nomenclature.

Please note that JCB now requires authors to submit Source Data used to generate figures containing gels and Western blots with all revised manuscripts. This Source Data consists of fully uncropped and unprocessed images for each gel/blot displayed in the main and supplemental figures. For assays performed using capillary electrophoresis and/or immunoassay-based detection, authors should instead provide the electropherogram graph(s) for each experiment, plotting fluorescence/chemiluminescence intensity vs. molecular weight/size. Please be sure to provide one Source Data file for each figure gels, blots, and/or capillary electrophoresis assays along with your revised manuscript files. File names for Source Data figures should be alphanumeric without any spaces or special characters (i.e., SourceDataF#, where F# refers to the associated main figure number or SourceDataFS# for those associated with Supplementary figures). For traditional gels and blots, the lanes of the gels/blots should be labeled as they are in the associated figure, the place where cropping was applied should be marked (with a box), and molecular weight/size standards should be labeled wherever possible. For capillary electrophoresis assays, each trace in the graph should be color-coded and labeled to indicate which protein, gene, or sample is being measured (please try to avoid red/green combinations to accommodate our color-blind readers).

Source Data files will be made available to reviewers during evaluation of revised manuscripts and, if your paper is eventually published in JCB, the files will be directly linked to specific figures in the published article.

Source Data Figures should be provided as individual PDF files (one file per figure). Authors should endeavor to retain a minimum resolution of 300 dpi or pixels per inch. Please review our instructions for export from Photoshop, Illustrator, and PowerPoint here: https://rupress.org/jcb/pages/submission-guidelines#revised

Journal of Cell Biology now requires a data availability statement for all research article submissions. These statements will be published in the article directly above the Acknowledgments. The statement should address all data underlying the research presented in the manuscript. Please visit the JCB instructions for authors for guidelines and examples of statements at (https://rupress.org/jcb/pages/editorial-policies#data-availability-statement).

B. FINAL FILES:

Please upload the following materials to our online submission system. These items are required prior to acceptance. If you

have any questions, contact JCB's Managing Editor, Lindsey Hollander (lhollander@rockefeller.edu).

-- An editable version of the final text (.DOC or .DOCX) is needed for copyediting (no PDFs).

-- High-resolution figure and MP4 video files: See our detailed guidelines for preparing your production-ready images, https://jcb.rupress.org/fig-vid-guidelines.

-- Cover images: If you have any striking images related to this story, we would be happy to consider them for inclusion on the journal cover. Submitted images may also be chosen for highlighting on the journal table of contents or JCB homepage carousel. Images should be uploaded as TIFF or EPS files and must be at least 300 dpi resolution.

It is JCB policy that if requested, original data images must be made available to the editors. Failure to provide original images upon request will result in unavoidable delays in publication. Please ensure that you have access to all original data images prior to final submission.

The license to publish form must be signed before your manuscript can be sent to production. A link to the license to publish form will be sent to the corresponding author only. Please take a moment to check your funder requirements before choosing the appropriate license.

Additionally, JCB encourages authors to submit a short video summary of their work. These videos are intended to convey the main messages of the study to a non-specialist, scientific audience. Think of them as an extended version of your abstract, or a short poster presentation. We encourage first authors to present the results to increase their visibility. The videos will be shared on social media to promote your work. For more detailed guidelines and tips on preparing your video, please visit https://rupress.org/jcb/pages/submission-guidelines#videoSummaries.

Thank you for your attention to these final processing requirements. Please revise and format the manuscript and upload materials within 14 days. If you need an extension for whatever reason, please let us know and we can work with you to determine a suitable revision period.

Please contact the journal office with any questions at cellbio@rockefeller.edu.

Thank you for this interesting contribution, we look forward to publishing your paper in Journal of Cell Biology.

Sincerely,

Matthew Welch
Monitoring Editor
Journal of Cell Biology

Gabriele Stephan
Scientific Editor
Journal of Cell Biology

Editorial statement:
Evaluation: I support an accept with revision decision. Overall, I agree with Reviewer #1 that the advance warrants publication. I do see Reviewer #3's point that the nature of the forces applied might not be the same as those experienced in cells. However, I feel that the effects of force are only part of what is new about the findings, and that the impact will come from adding to our understanding of how arp2/3 complex nucleotide state affects branch dynamics more generally.
Recommendation: I think the manuscript needs a bit of work.
The manuscript needs to be carefully edited, as there are many typos. Moreover, it would benefit from shortening. In some instances, a bit of reorganizing would be helpful in my view. I have three specific suggestions in this regard: (1) shorten the results by eliminating some of the discussion-type text or moving to the discussion; (2) move the model from Figure 5E to Figure 8, and contextualize and discuss the nucleotide data and the GMF/cortactin data more in the discussion than in the results; (3) move the introductory paragraphs about GMF and cortactin from their respective sections of the results to the introduction and shorten.
I also have some specific comments:
(1) Modify the last sentence of the abstract. It makes it seem like the major advance relates to a possible regulatory role for cytoplasmic Pi, which I think is too speculative. I would say to focus more on the advance of understanding the role of nucleotide state in branch dynamics.
(2) Get rid of most of the "remarkably" and "surprisingly" statements. The authors have convinced us already their work is interesting.

(3) Change "pulling" forces or "pull" in the text, as I don't think flow is really a pull, but rather a push. Maybe use "flow force" or something like that.
(4) Make the text in the figures bigger. It is tiny.
(5) References should be included when the authors refer specific ideas such as the "Bell-Evans model" or "Curtin-Hammett principle."

Antoine Jégou
Principal Investigator
'Regulation of Cytoskeleton Dynamics' Lab
antoine.jegou@ijm.fr / phone: +33.(0)1.57.27.80.13

Manuscript number: 202605018R
Corresponding author(s): Antoine, Jégou, Guillaume Romet-Lemonne

June 10th, 2026

Dear Editor,

We are submitting our revised manuscript to the Journal of Cell Biology. We have modified the manuscript according to the editorial recommendations as follows:

1. We have shortened the abstract and rephrased the last sentence to state the importance of the nucleotide state in Arp2/3 complexes;
2. We have removed all the 'remarkably' (1 occurrence) and 'surprisingly' (2 occurrences) statements;
3. We have substantially increased the size of the text in all figures;
4. We added references to the "Bell-Evans model" or "Curtin-Hammett principle" statements;
5. We have moved introductory elements on GMF and Cortactin initially in the Results section to the introduction part;
6. We have fused the 16 supplementary figures in order to have only 6.
7. We have moved Figure 5E to figure 8;
8. We have removed the following two sentences in the Results section:
    a. "Our measured Pi release rate from Arp2/3 complex at branch junction is also significantly much faster than Pi release from mammalian alpha- and beta-actin subunits within actin filaments (0.007 s-1 (Fujiwara et al., 2007; Jégou et al., 2011; Melki et al., 1996; Oosterheert et al., 2023; Schahl et al., 2025)) (see Discussion)."
    b. "Cortactin also reinforces the interface with the mother filament. Overall, we confirm that cortactin stabilizes branches, but our measurements provide new unanticipated molecular details of how cortactin does this (see Discussion)."

Regarding the point (3) raised by the editor ('Change "pulling" forces or "pull" in the text, as I don't think flow is really a pull, but rather a push. Maybe use "flow force" or something like that'), we think that what matters most is that the force being applied to the actin filaments or

the branch junctions is a tension (a tensile force, not a compressive force). Furthermore, we also believe it is important to use the same terminology as in all other articles that employ microfluidics to apply a tensile force to actin filaments (from our lab, but also from the labs of Tom Pollard (Courtemanche et al, PNAS 2013), Enrique De la Cruz (Pandit et al, PNAS 2020; Bibeau et al, PNAS 2023), or Shashank Shekhar (Reddy et al, PNAS 2025)), as well as in other biophysics fields (*eg* DNA stretching assays).

Best regards,
Antoine Jégou, on behalf of the authors

Antoine Jégou

INSTITUT JACQUES MONOD
Unité mixte de recherche du CNRS
et de l'université Paris Cité

Bâtiment Buffon
15 rue Hélène Brion
75013 Paris, France

Phone +33 1 57 27 81 42
ijm@ijm.fr
